# A review of the Madagascan pelican spiders of the genera *Eriauchenius* O. Pickard-Cambridge, 1881 and *Madagascarchaea* gen. n. (Araneae, Archaeidae)

**DOI:** 10.3897/zookeys.727.20222

**Published:** 2018-01-11

**Authors:** Hannah M. Wood, Nikolaj Scharff

**Affiliations:** 1 Smithsonian Institution, National Museum of Natural History, 10th St. and Constitution Ave. NW, Washington, DC 20560-0105, USA; 2 Biodiversity Section, Center for Macroecology, Evolution and Climate, Natural History Museum of Denmark, University of Copenhagen, Universitetsparken 15, DK-2100 Copenhagen, Denmark

**Keywords:** Afrarchaea, Palpimanoidea, new species, taxonomy

## Abstract

An endemic genus of Madagascan spiders (Araneae, Archaeidae, *Eriauchenius*) is revised. All 20 species of *Eriauchenius* are described and keyed, of which 14 are new species: *Eriauchenius andriamanelo* **sp. n.**, *Eriauchenius andrianampoinimerina* **sp. n.**, *Eriauchenius goodmani* **sp. n.**, *Eriauchenius harveyi* **sp. n.**, *Eriauchenius lukemacaulayi* **sp. n.**, *Eriauchenius milajaneae* **sp. n.**, *Eriauchenius milloti* **sp. n.**, *Eriauchenius rafohy* **sp. n.**, *Eriauchenius ranavalona* **sp. n.**, *Eriauchenius rangita* **sp. n.**, *Eriauchenius rixi* **sp. n.**, *Eriauchenius sama* **sp. n.**, *Eriauchenius wunderlichi* **sp. n.**, *Eriauchenius zirafy* **sp. n.** Additionally, six species of the new genus *Madagascarchaea* **gen. n.** are described and keyed, of which four are new species: *Madagascarchaea fohy* **sp. n.**, *Madagascarchaea lotzi* **sp. n.**, *Madagascarchaea moramora* **sp. n.**, *Madagascarchaea rabesahala* **sp. n.** Diagnostic characters for the Madagascan and African genera are described, and based on these characters and previous phylogenetic analyses the following species transfers are proposed: *Eriauchenius cornutus* (Lotz, 2003) to *Afrarchaea*; *Afrarchaea fisheri* (Lotz, 2003) and *Afrarchaea mahariraensis* (Lotz, 2003) to *Eriauchenius*. Finally, we propose that the distribution of *Afrarchaea* be restricted to South Africa. While several Madagascan specimens have previously been identified as *Afrarchaea godfreyi* (Hewitt, 1919), we argue that these are likely misidentifications that should instead be *Eriauchenius*.

## Introduction

Archaeid spiders, commonly called pelican or assassin spiders, are an ancient, paleoendemic group that has existed since Pangaean times (Wood et al. 2013). These spiders do not build a web to capture their prey. Instead, they are active hunters and they have an unusual modification to their cephalic area that relates to their predatory behaviors: the carapace is extended and tubular in structure, and encircles the cheliceral bases, giving archaeids the appearance of a ‘‘neck’’ and “head”; the chelicerae are also greatly elongated. While most spiders are predatory generalists (Foelix 2011), archaeids are highly specialized and will only prey on other spiders (Legendre 1961; Millot 1948; Wood 2008; Wood et al. 2012). The highly modified cephalic area in archaeids is used to employ a novel prey capture strategy that is unique among spiders (see fig. 1 in Wood et al. 2007): the modified carapace allows for highly maneuverable and elongated chelicerae that can be extended 90° away from the body to attack spider prey at a distance (Forster and Platnick 1984; Wood et al. 2012). There is a diversity of carapace shapes among archaeids, with ‘‘necks’’ of varying degrees of elongation, from long and constricted to short and stout. Reliance on the elevated cephalic area as a phylogenetically informative character has served as the basis for historical classifications of archaeid spiders and their closest relatives (Forster and Platnick 1984; Legendre 1970; Platnick 1991). However, it has been shown that different morphs with varying degrees of elevation in the cephalic area have evolved in parallel within the family (Wood et al. 2015; Wood et al. 2007).

The morphological interspecific diversity in the carapace and chelicerae shape seems to relate to their global distribution and diversification patterns. Archaeid spiders have distinct Northern Hemisphere lineages, now extinct and known only from fossils dated from the Eocene to the Jurassic (Koch and Berendt 1854; Penney 2003; Selden et al. 2008; Wood 2017; Wunderlich 2004, 2008, 2015). The extant lineages occur only in the Southern Hemisphere, restricted to Madagascar, mainland Australia, and South Africa (World Spider Catalog 2017). A divergence dating study that included both fossil and extant taxa concluded that the Southern Hemisphere lineages are monophyletic and that the split between the extinct northern and extant southern faunas likely relates to Pangaea breaking into Gondwana and Laurasia in the Jurassic (Wood et al. 2013). So, it appears that archaeids were once more widespread, but are currently restricted to relictual areas. Furthermore, there has been a shift in morphological features through time: the fossil archaeids in general had shorter carapace/chelicerae features, and occupied a unique region of archaeid morphospace, whereas the extant clades have more elongated features (see fig. 5 in Wood 2017). While the northern lineages went extinct, the southern lineages persisted, and within their present day relictual distributions the extant clades have diversified. Recent studies have recovered the timing of diversification in the extant Australian clade to be congruent with Miocene aridification (Rix and Harvey 2012c), and in the South African clade to be congruent with Miocene uplift of the Great Escarpment (Wood et al. 2015). Madagascan lineages have been exposed to more ancient and more turbulant geoclimatic events, and compared to the Australian and South African clades, the Madagascan clades show an increased rate of morphological evolution in carapace and chelicerae shape (Wood et al. 2015). Furthermore, there is a greater degree of sympatry among Madagascan species than is observed in the Australian and South African species. Morphological diversification in the Madagascan clade may be due to exposure to more ancient geoclimatic events that led to the build-up of sympatric species in montane rainforest areas (Wood et al. 2015).

**Figure 1.**
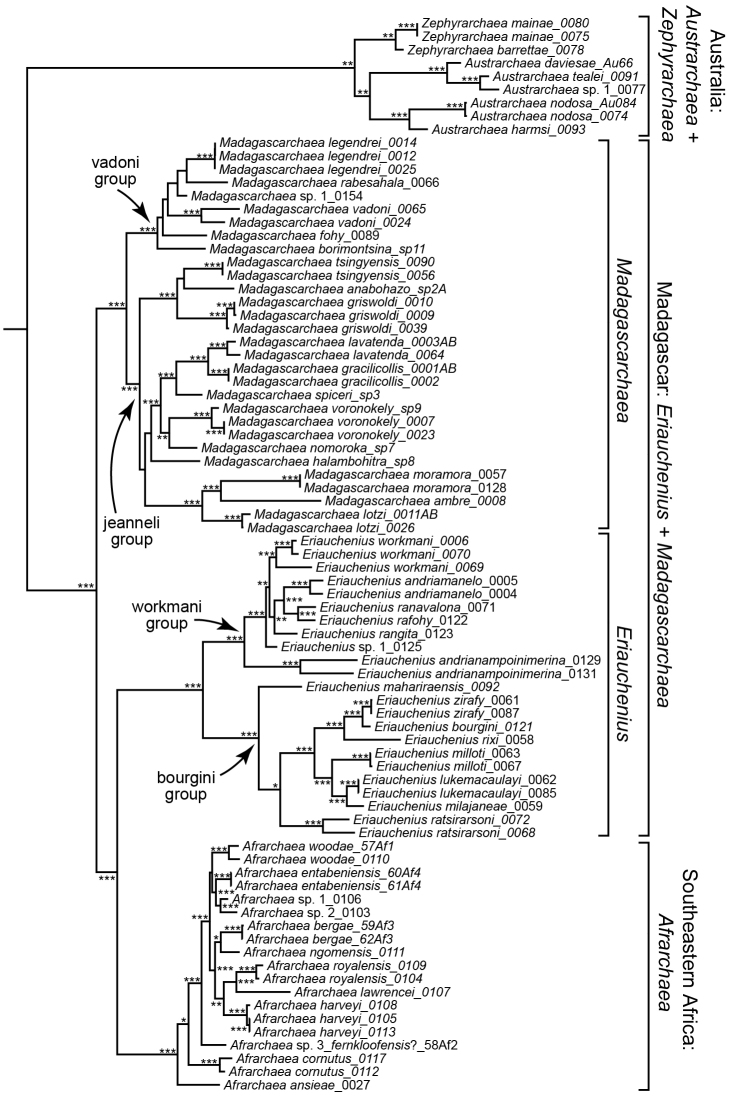
Total evidence phylogeny from Bayesian analysis of molecular and morphological data, from Wood et al. (2015). Outgroups not shown and with the morphospecies names changed to the new species names from the current study. Posterior probabilities are as follows, greater than or equal to: (*) 0.90; (**) 0.95; (***) 0.99.

Phylogenetic analysis of molecular and morphological data by Wood et al. (2012) placed archaeids in the superfamily Palpimanoidea along with four other families. However, recent phylogenetic analyses of molecular data failed to recover this same Palpimanoidea clade, although, the monophyly of Archaeidae is strongly supported and not disputed (Wheeler et al. 2016). Since Forster and Platnick (1984) delimited the family to 17 species, there have been subsequent revisions that have expanded our knowledge of archaeid species diversity: the Australian archaeids are nearly completely revised (Rix and Harvey 2011, 2012a, b) for a total of 38 species; 12 South African species have been described (Lotz 1996, 2003, 2006, 2015), although new species are still being discovered (Wood et al. 2015); the “gracilicollis group” from Madagascar was revised (Wood 2008) and contains 14 species; and here, we perform a revision of the Madagascan *Eriauchenius*. A recent phylogenetic analysis (Wood et al. 2015) resolved the relationships among the extant genera with strong branch support: the Australian lineages were found to be monophyletic and sister to the African + Madagascar species; there are two monophyletic groups on Madagascar, *Eriauchenius* and *Madagascarchaea* gen. n.; the South African clade *Afrarchaea* Forster & Platnick, 1984 is sister to *Eriauchenius*, and *Afrarchaea* + *Eriauchenius* is sister to *Madagascarchaea* gen. n. The phylogeny from Wood et al (2015) is reproduced here, but edited to include the names of the new genus and the newly described species (Fig. 1). Based on this phylogeny as well as the morphological examination of species, in the current study we propose several taxonomic changes: first, the creation of a new genus *Madagascarchaea* gen. n., which was previously revised as the “gracilicollis group” (Wood 2008); and second, the transfer of several species to different genera. The current paper also performs a taxonomic revision of *Eriauchenius*, describing 14 new species, and additionally 4 new species of *Madagascarchaea* gen. n.

## Methods

**Figure 2.**
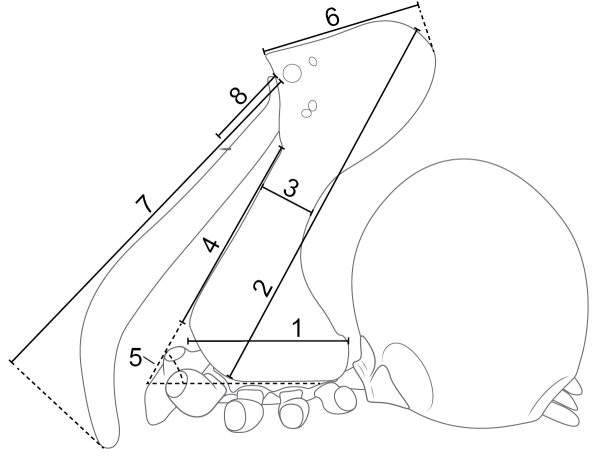
Diagram showing several measured morphological traits, lateral view of archaeid habitus. **1** carapace length **2** carapace tilt height (CtH) **3** carapace constriction **4** “neck” length **5** carapace tilt angle **6** “head” length **7** chelicerae length **8** cheliceral seta length.

Specimens examined in this study were primarily from the California Academy of Sciences collection. Additional material was borrowed from the museums referred to in Table 1, which lists the museum abbreviations used in the text. Scanning electron microscope (SEM) images were taken using a Leo 1450VP, a JEOL JSM-6335F, and a Zeiss EVO MA15 scanning electron microscope; prior to photographing, specimens were critically point dried and sputter coated. Specimens were also examined and interpretively drawn using a Leica MZ16 and M205C microscope that had a drawing tube attached. The vulva, after dissection, was placed overnight in Ultrazyme contact lens cleaner. Photographs were taken as a series of stacks using a Nikon DXM1200 or a Canon EOS T6i digital camera mounted on the Leica microscope or using a BK Plus Laboratory System from Visionary Digital (Palmyra, PA, USA) equipped with a Canon EOS 7D camera. Image stacks were assembled using stacking software Auto-Montage Pro (Syncroscopy, Digital Imaging Systems Ltd.), HeliconFocus (Helicon Soft Ltd.) or ZereneStacker (Zerene Systems, LLC). All measurements are in millimetres (mm). Morphological abbreviations used in the text are listed in Table 2. The pars cephalica in Archaeidae is elongated and the term ‘head’ or ‘cephalon’ represents the most distal portion of the elongation, and the term ‘neck’ represents the constricted portion of the elongation. The measurement CtH is the length from the lateral edge of the carapace between coxae I and II to the top of the cephalon, running parallel with the “neck” (Fig. 2). This measurement takes the tilt of the ‘neck’ into account. The carapace tilt angle is defined as the angle between the posterior edge of the lateral side of the carapace (the portion above coxae II and III) and the anterior edge of the “neck” in the lateral view (Fig. 2). The CtH/CL ratio quantifies the elongation of the cephalic area by dividing the CtH by the carapace length. The female genitalia have a sclerotic structure called the female sclerotized genital plate (FSGP) that is attached dorsally to the bursa, and of unknown use. The term ‘wings’ (W) describes the flat, fan-like projection extending to each lateral side of the FSGP, and the term ‘posterior bar’ refers to a curved sclerotized piece that sits posterior to the FSGP in some *Eriauchenius*. The male pedipalpal bulbs all have a conductor (C), and we believe what we are calling the conductor in this study is homologous to the conductor in Rix and Harvey (2011, 2012a, b), and to the bulb proapical process (BPAP) in Wood (2008). In some species there is an additional sclerite that we abbreviate SC that is likely an additional process of the conductor through a membranous connection. In some species we describe a median apophysis (MA) for convention, however we are not sure of the homology of this structure. We believe that the MA may be homologous to the ‘bulb dorsal sclerite’ (BDS) that was documented in *Madagascarchaea* gen. n. in Wood (2008). In *Madagascarchaea* gen. n. the sclerite that we call S1 was termed the ‘bulb lateral sclerite’ (BLS) in Wood (2008). The homology of archaeid pedipalpal parts is unclear and the terminology used here is not necessarily an argument for homology.

**Table 1.** List of instituition abbreviations used in the text.

**AMNH**	American Museum of Natural History, New York
**BMNH**	The Natural History Museum, London
**CAS**	California Academy of Sciences, San Francisco
**CASENT**	California Academy of Sciences, Entomology Department
**FM**	Field Museum, Chicago
**MNHN**	Muséum National D'Historie Naturelle, Paris
**USNM**	National Museum of Natural History, Smithsonian Institution, Washington DC
**USNMENT**	National Museum of Natural History, Entomology Department

**Table 2.** List of anatomical abbreviations used in the text and figures.

**a**	anterior portion of embolus
**AME**	anterior median eye
**C**	conductor
**CtH**	carapace tilt height
**E**	embolus
**FSGP**	female sclerotized genital plate
**LE**	lateral eye
**M**	membranous area on male pedipalpal bulb
**MA**	median apophysis
**p**	posterior portion of embolus
**PB**	posterior bar
**PP**	poreplates
**S1**	additional sclerite on male pedipalpal bulb in Madagascarchaea
**SC**	pedipalpal bulb sclerite, likely a part of the conductor
**W**	wings, lateral projections on FSGP

## Taxonomy

### Archaeidae

Taxon classificationAnimaliaAraneaeArchaeidae

Koch & Berendt, 1854

#### Diagnosis.

Ecribellate, haplogyne, araneomorph spiders, three claws, with peg teeth, with cheliceral and pedicel stridulatory systems; modified carapace that wraps around the base of the chelicerae forming a constricted neck; extant genera with set of anterior booklungs and pair of posterior spiracles (configuration unknown in fossil archaeids). For complete description see Forster & Platnick (1984).

#### Discussion.

While archaeids have a substantial fossil record (12 genera; Dunlop et al. 2017) from the Northern Hemisphere, the extant Southern Hemisphere genera form a well-supported monophyletic group based on morphology and molecular data (Wood et al. 2012; Wood et al. 2013). The extant clade can be distinguished from the fossil genera by the following combination of characters (Wood et al. 2012): presence of a tubercle on the sternum, which may be implicated in the pedicel stridulatory system that has been observed in the extant genera; the posterior edge of the carapace is truncated in the extant clades rather than tapering off as in the fossils; the distal portion of the chelicerae are curved towards the posterior; the presence of a brush of hairs on the prolateral side of the pedipalpal tarsi which interacts with cheliceral stridulatory file (Wood 2008), whereas fossils have only stridulatory picks (*Madagascarchaea gracilicollis* (Millot, 1948) is the exception, having both stridulatory picks and a brush of hairs). The extant archaeids are comprised of the following genera: *Austrarchaea* Forster & Platnick, 1984, and *Zephyrarchaea* Rix & Harvey, 2012a, from Australia; *Eriauchenius*, and *Madagascarchaea* gen. n., from Madagascar; and *Afrarchaea* Forster & Platnick, 1984, from South Africa. The Australian taxa (*Austrarchaea* and *Zephyrarchaea*) can be distinguished from the African and Madagascan taxa by the numerous spermathecae in females, the lack of a FSGP that sits immediately dorsal to the bursa, and the long wiry embolus on the male pedipalps (Forster and Platnick 1984; Rix and Harvey 2011, 2012a, b; Wood 2008). *Zephyrarchaea* can be distinguished from *Austrarchaea* by the carapace height to carapace length ratio being less than 2, and by the presence of accessory setae on or adjacent to the proximal cheliceral paturon bulge in males (Rix and Harvey 2012a). In females of the African and Madagascan genera there is a bursa with clusters of secretory pores, typically on the anterior and dorsal side of the bursa, and a FSGP is also present immediately adjacent to the dorsal side of the bursa.

### Genus Eriauchenius

Taxon classificationAnimaliaAraneaeArchaeidae

O. P.-Cambridge, 1881

Eriauchenius O. P.-Cambridge, 1881: 767. Simon 1895: 935. Wunderlich 2004: 778, 791.

#### Type species.

*E. workmani* O. P.-Cambridge, 1881, by original designation.

#### Diagnosis.

Distinguished from the Australian genera by lacking spermathecae in the female genitalia, and instead having a bursa with secretory poreplates and a FSGP, and, in males, lacking the long wiry embolus on the male pedipalps seen in the Australian species. Distinguished from *Afrarchaea* by lacking the perpendicular keel on the FSGP, and from *Madagascarchaea* gen. n. by having 2–4 spines on the apex of the cephalon instead of 6, and lacking the retrolateral apophysis on the male pedipalpal patella.

#### Description.

Total length 1.64–6.72. Carapace reddish-to-orangish brown with many white setae on small tubercules, organized in branching rows (see figs 5D, 6B in Wood 2008); pars cephalica elongated forming ‘head’ (distal portion of elongation) and ‘neck’ (constricted portion of elongation), with carapace tilt height divided by carapace length 1.44–2.85, with the angle of carapace tilt 54.7°–88.1°; with a pair of posterior, and often with an anterior pair, of pronounced-to-rudimentary protrusions on apex of cephalon or ‘head,’ each with a small-to-large spine; neck with fissure on anterior side running from chelicerae bases to labrum (see fig. 6B in Wood 2008). AME on a small-to-large bulge with a point or rounded at apex (see fig. 8C in Wood 2008). AME diameter larger than all other eyes; median ocular quadrangle (MOQ) wider in front than behind or than long; lateral eyes contiguous; sometimes with a short spine between median eyes and LE (see figs 8C and 18 in Wood 2008). Sternum reddish-to-orangish brown and longer than wide, hollowed out around coxae, with a border; setae with tuberculate bases; with expanded tubercle on posterior part of sternum, close to 4th coxae (see fig. 9F in Wood 2008), possibly implicated in the abdomen-petiole stridulatory system. Long sclerite between coxae and carapace (see fig. 7C in Wood 2008). Endites converging; serrula strongly pointed; labrum with two lateral projections on dorsal surface (see fig. 6A in Wood 2008). Small round chilum sclerite next to each cheliceral base. One triangular sclerite between and posteriad to the cheliceral bases, and an additional sclerite running along the length of the triangular sclerite base (see fig. 7A in Wood 2008). Chelicerae with a pronounced-to-rudimentary anterior protrusion with a downward or perpendicular pointing long-to-short spine, cheliceral spine/chelicerae length ratio 0.068–0.49; with stridulatory ridges on lateral side, and chelicerae curved to the posterior distal to the stridulatory ridges. The structure used in conjunction with the cheliceral stridulatory file appears to be a group of modified hairs on the prolateral side of the palp (Forster and Platnick 1984; Lotz 2003; Wood 2008); it is unknown whether sclerotized structures on the male palpal bulb are also used in conjunction with the cheliceral file, which has been observed in *Madagascarchaea* gen. n. (Wood 2008). Peg teeth in three rows; anterior row with two peg teeth and posterior row of two-three, both sitting opposite fang tip, median row of 18–30, strongest distally and gradually grading to normal setae. Teeth on retromargin 2–7, may have different numbers of teeth per chelicera on same individual.

**Figure 3.**
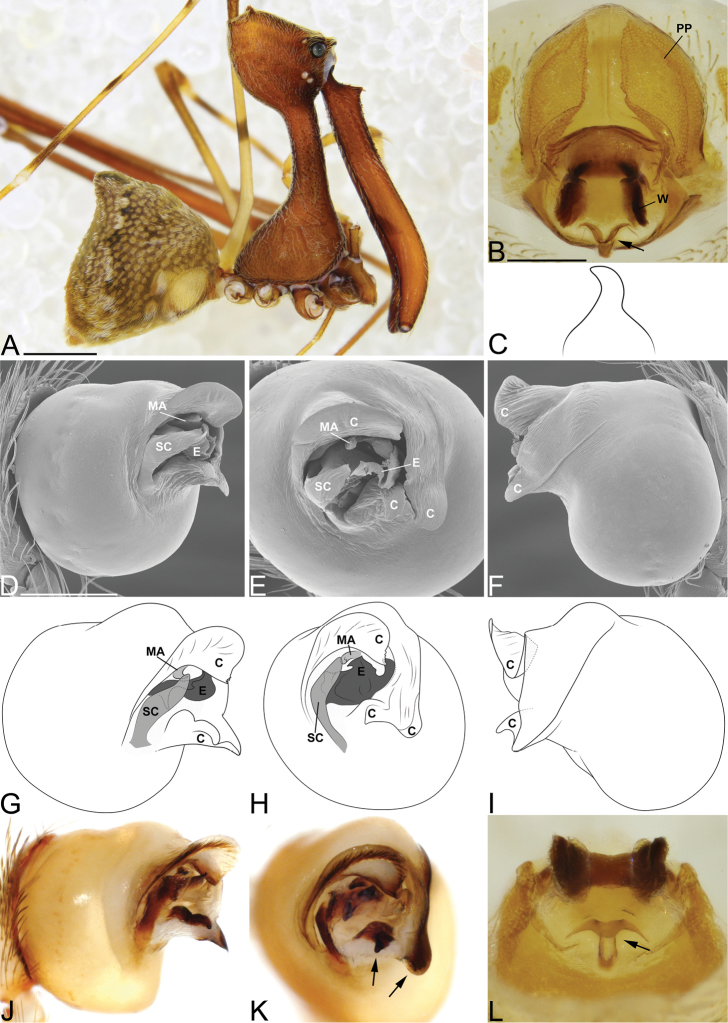
*Eriauchenius andriamanelo* sp. n. **A** male (CASENT9004508) habitus, lateral view. **B, L** female (CASENT9003265) internal genitalia, arrow showing sclerotized T-shaped projection: **B** dorsal view **L** posterior view **D–F** male right pedipalpal bulb (CASENT9010070), image reversed **C, G–I** left pedipalpal bulb (CASENT9017991) **J–K** left pedipalpal bulb (CASENT9002367) **C** median apophysis, apical view **D, G, J** prolateral view **E, H, K** ventral view **F, I** retrolateral view **K** arrows showing the two portions of the divided conductor tip. Scale bars: 1 mm (**A**); 0.25 mm (**B, D**).

**Figure 4.**
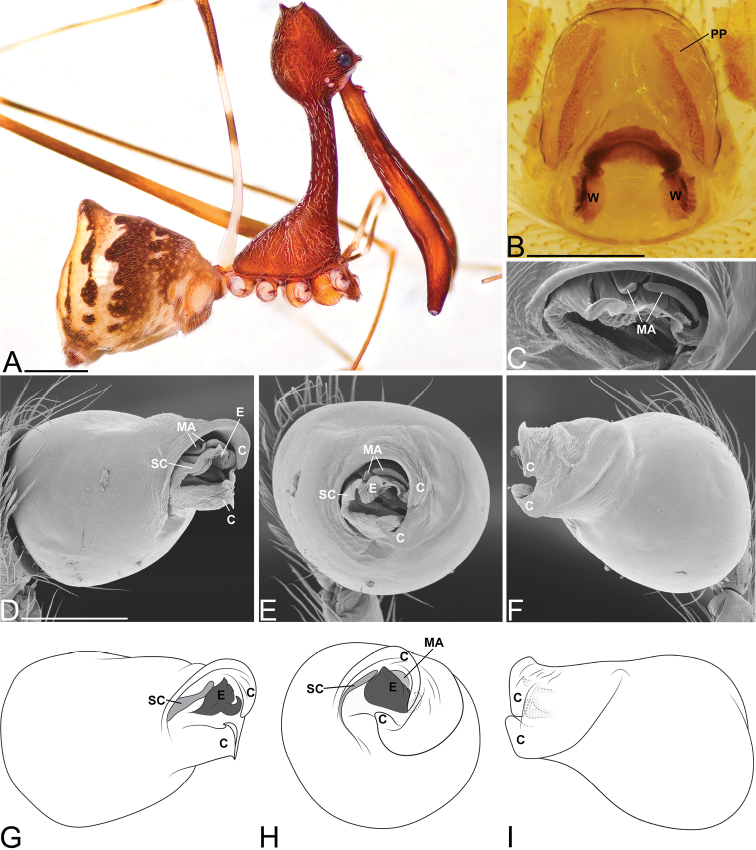
*Eriauchenius andrianampoinimerina* sp. n. **A** female (CASENT9018902) habitus, lateral view, image reversed **B** female (CASENT9015417) internal genitalia, dorsal view **C–F** male right pedipalpal bulb (CASENT9015371), image reversed **G–I** left pedipalpal bulb (CASENT9015518) **C** median apophysis close-up, apical view **D, G** prolateral view **H, K** ventral view **I, L** retrolateral view. Scale bars: 1 mm (**A**); 0.25 mm (**B, D**).

**Figure 5.**
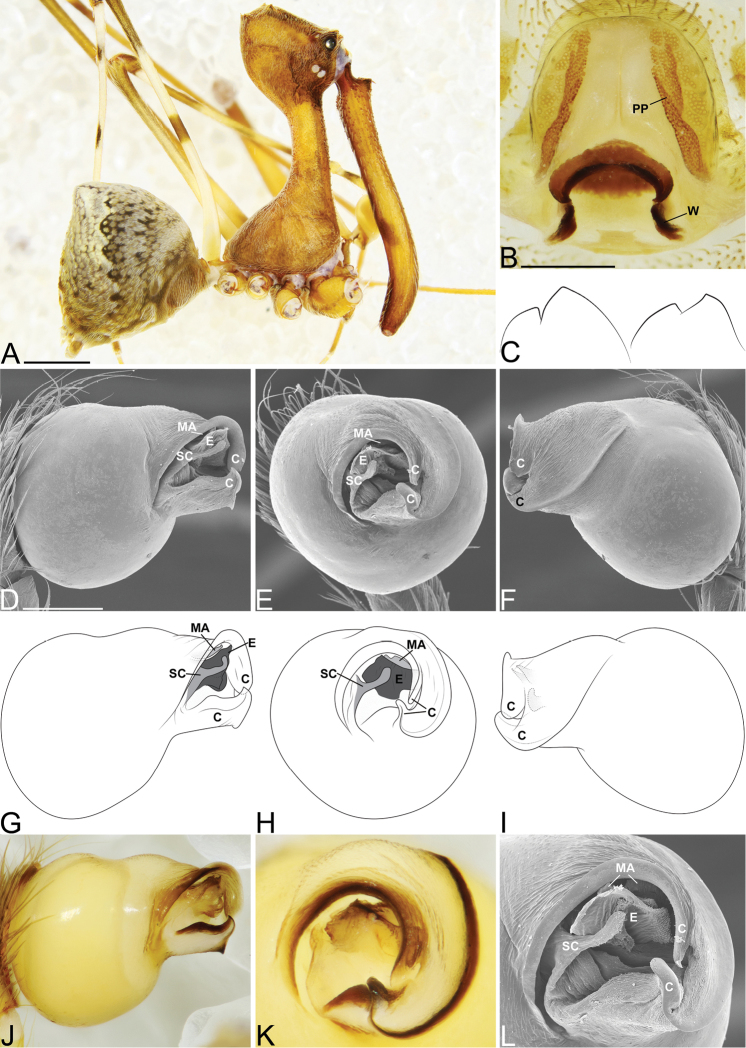
*Eriauchenius rafohy* sp. n. **A** male (CASENT9015039) habitus, lateral view, image reversed **B** female (CASENT9006503) internal genitalia, dorsal view **D–F, L** male right pedipalpal bulb (CASENT9004087), image reversed **G–K** left pedipalpal bulb (CASENT9015039) **C** median apophysis variation, apical view (CASENT9004087 & CASENT9015039) **D, G, J** prolateral view **E, H, K** ventral view **F, I** retrolateral view **L** close-up, ventral view. Scale bars: 1 mm (**A**); 0.25 mm (**B, D**).

**Figure 6.**
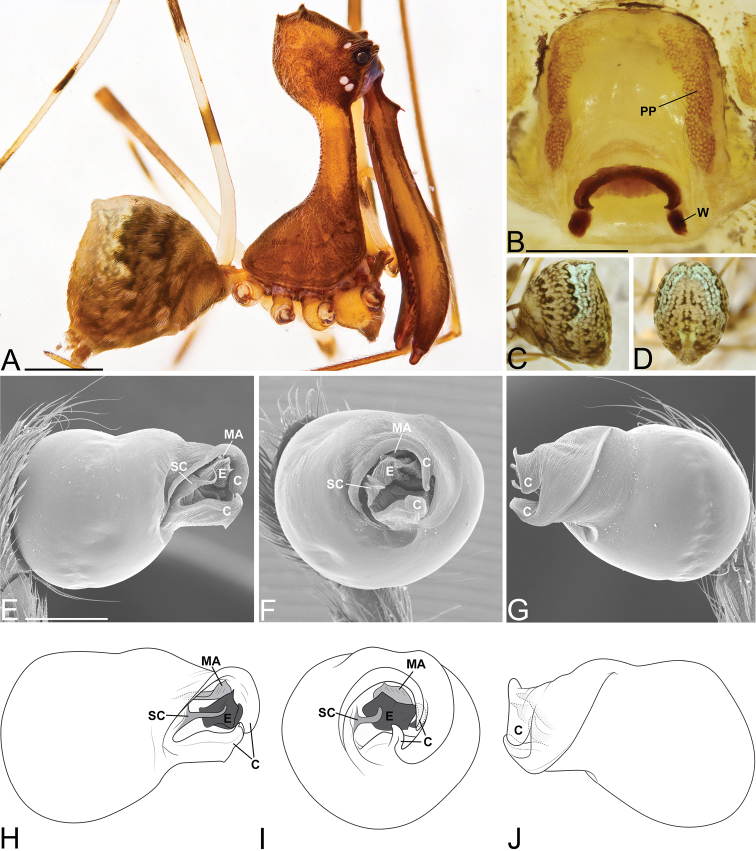
*Eriauchenius ranavalona* sp. n. **A** male (holotype, CASENT9010047) habitus, lateral view, image reversed **B** female (USNMENT01377248) internal genitalia, dorsal view **C–D** abdomen, showing color pattern (USNMENT01377246) **C** lateral view **D** posterior view **E–G** male right pedipalpal bulb (holotype, CASENT9010047), image reversed **H–J** left pedipalpal bulb (CASENT9010042) **E, H** prolateral view **F, I** ventral view **G, J** retrolateral view. Scale bars: 1 mm (**A**); 0.25 mm (**B, D**).

**Figure 7.**
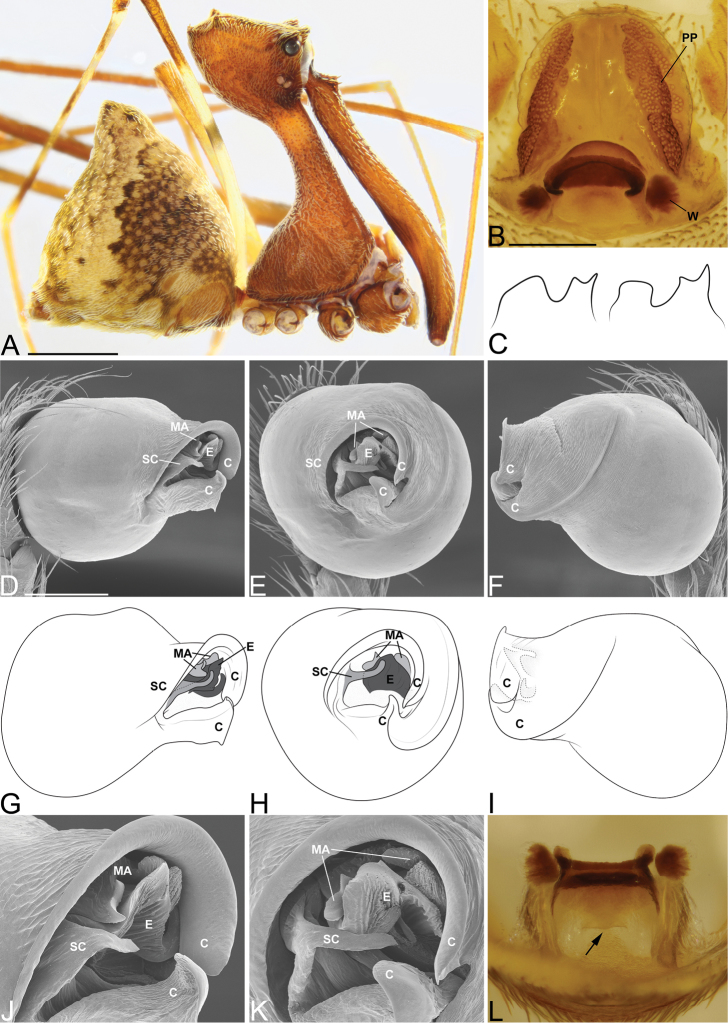
*Eriauchenius rangita* sp. n. **A** male (CASENT9018159) habitus, lateral view **B, L** female (CASENT9018159) internal genitalia **B** dorsal view **L** posterior view, arrow showing membranous projection on bursa **D–F, J–K** male right pedipalpal bulb (CASENT9018251) image reversed **G–I** left pedipalpal bulb (CASENT9018159) **C** median apophysis variation, apical view (CASENT9018159 & CASENT9010053, holotype) **D, G** prolateral view **E, H** ventral view **F, I** retrolateral view **J** close up, prolateral view **K** close-up, ventral view. Scale bars: 1 mm (**A**); 0.25 mm (**B, D**).

**Figure 8.**
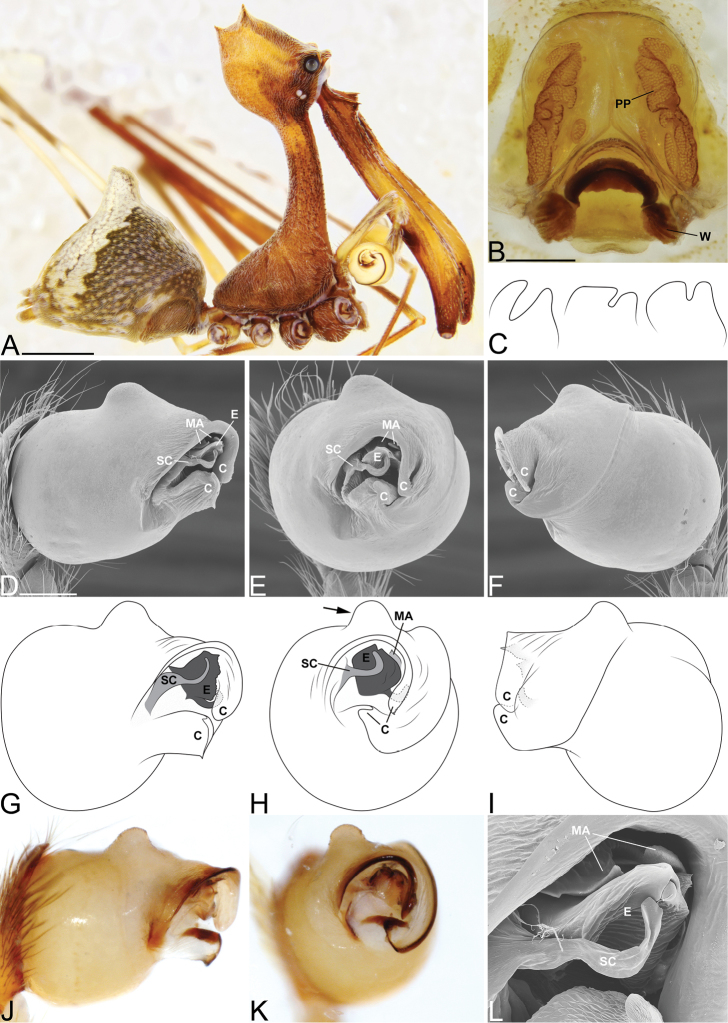
*Eriauchenius workmani* Pickard-Cambridge, 1881. **A** male (CASENT9010050) habitus, lateral view **B** female (CASENT9010045) internal genitalia, dorsal view **D–F, L** male right pedipalpal bulb (CASENT9010058) image reversed **G–I** left pedipalpal bulb (CASENT9003490) **J–K** left pedipalpal bulb (CASENT9010056) **C** median apophysis variation, apical view **D, G, J** prolateral view **E, H, K** ventral view, arrow showing the large bump on the anterior side **F, I** retrolateral view **K** close-up, ventral view. Scale bars: 1 mm (**A**); 0.25 mm (**B, D**).

**Figure 9.**
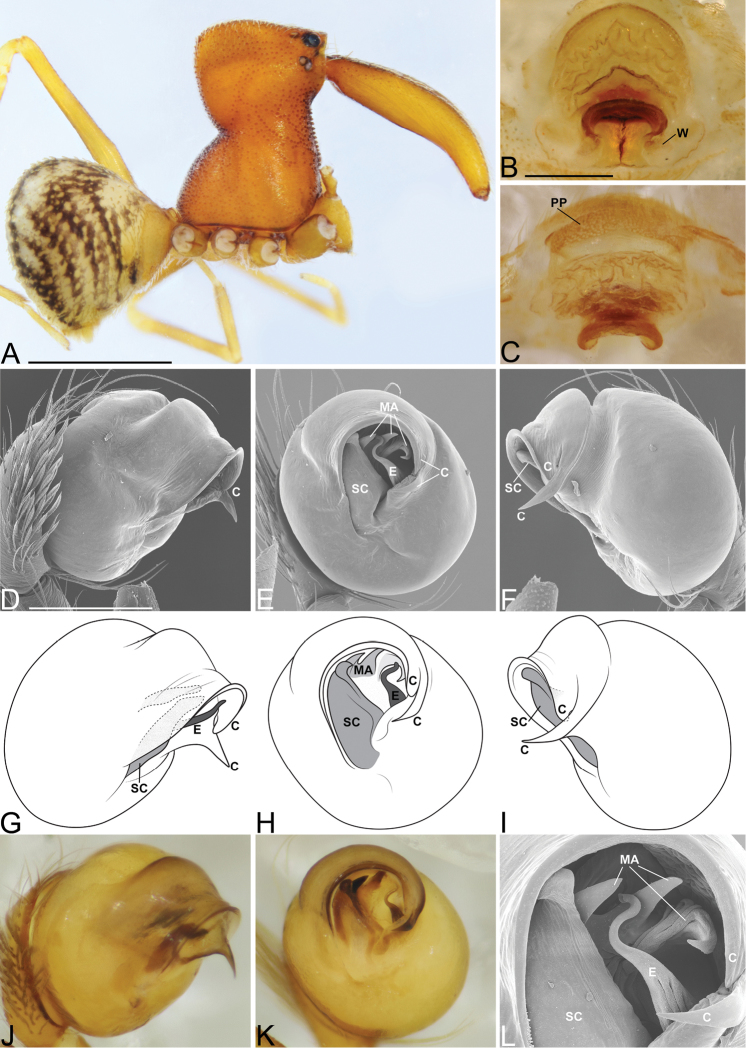
*Eriauchenius fisheri* (Lotz, 2003). **A** male (CASENT9018939) habitus, lateral view, image reversed **B–C** female (holotype, CASENT9012340) internal genitalia **B** dorsal view **C** anterior view **D–L** male pedipalpal bulbs (CASENT9018939) **D–F, L** right bulb, image reversed **G–K** left bulb **D, G, J** prolateral view **E, H, K** ventral view **F, I** retrolateral view **K** close-up, ventral view. Scale bars: 1 mm (**A**); 0.25 mm (**B, D**).

**Figure 10.**
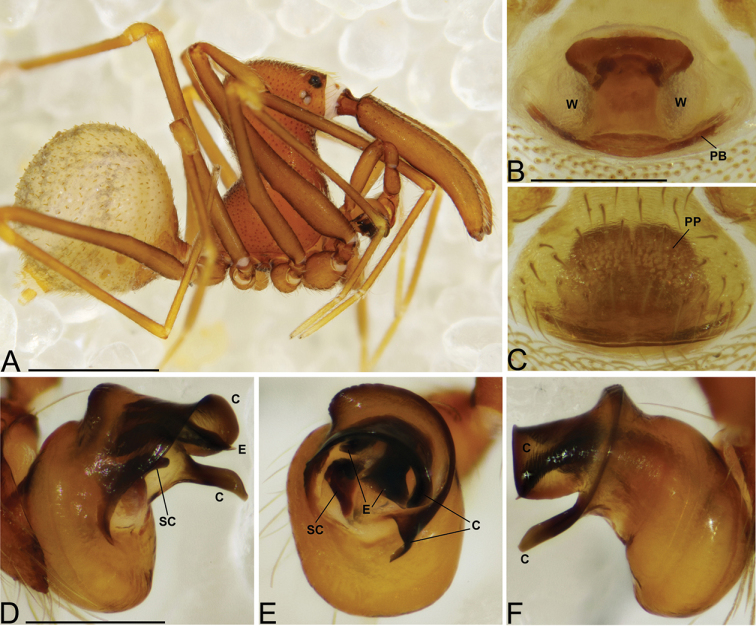
*Eriauchenius goodmani* sp. n. **A** male (holotype, Field Museum) habitus, lateral view **B–C** female (Field Museum) genitalia **B** dorsal view **C** ventral view **D–F** male left pedipalpal bulb (holotype, Field Museum) **D** prolateral view **E** ventral view **F** retrolateral view. Scale bars: 1 mm (**A**); 0.25 mm (**B, D**).

**Figure 11.**
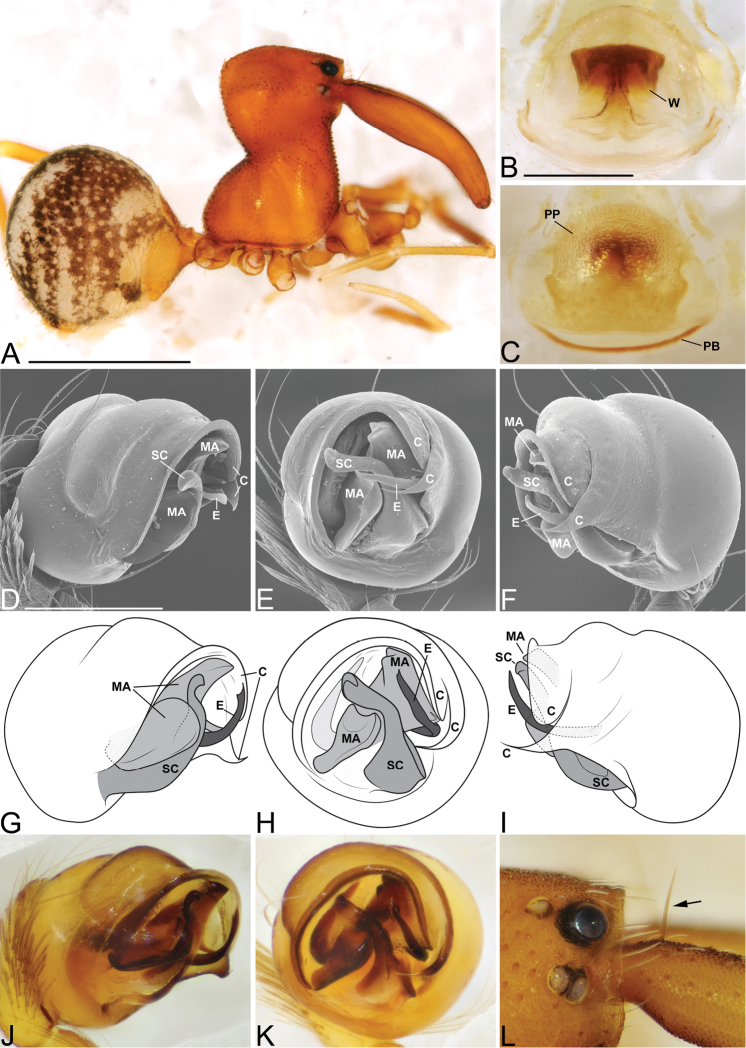
*Eriauchenius harveyi* sp. n. **A** male (holotype, CASENT9012110) habitus, lateral view, image reversed **B–C** female (CASENT9012342) genitalia **B** dorsal view **C** ventral view **D–K** male pedipalpal bulbs (CASENT9012109) **D–F** right bulb, image reversed **G–K** left bulb **D, G, J** prolateral view **E, H, K** ventral view **F, I** retrolateral view **L** male (CASENT9012109), arrow showing the long cheliceral seta that projects perpendicularly from the chelieral cuticle, lateral view. Scale bars: 1 mm (**A**); 0.25 mm (**B, D**).

**Figure 12.**
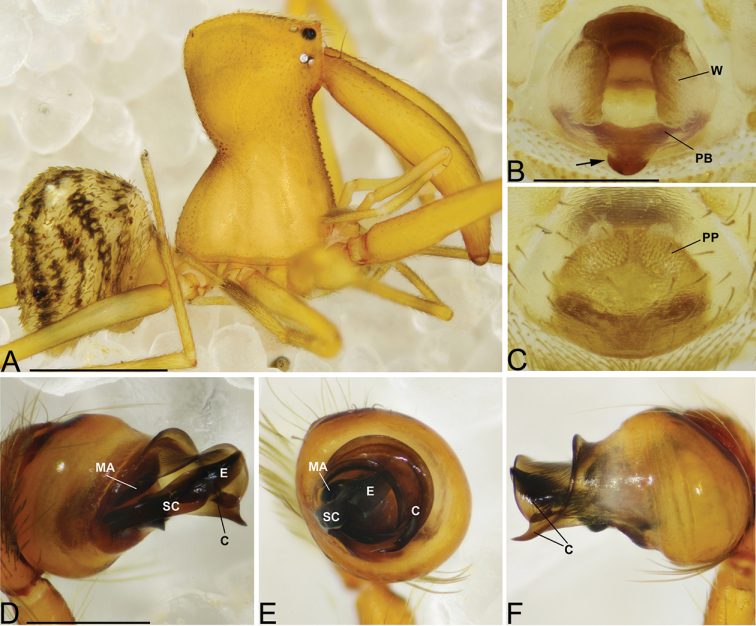
*Eriauchenius wunderlichi* sp. n. **A** female (CASENT9062648) habitus, lateral view, image reversed **B–C** female (CASENT9062648) genitalia **B** dorsal view, arrow showing the large bulge on the posterior bar **C** ventral view **D–F** male left pedipalpal bulb (holotype, CASENT9062702) **D** prolateral view **E** ventral view **F** retrolateral view. Scale bars: 1 mm (**A**); 0.25 mm (**B, D**).

**Figure 13.**
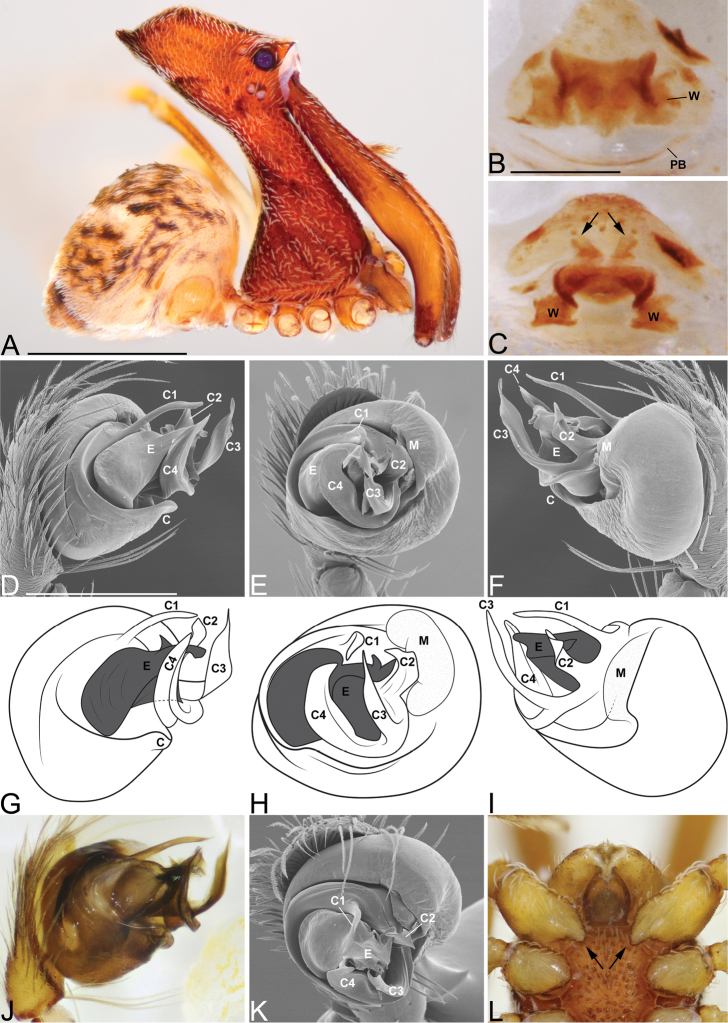
*Eriauchenius bourgini* (Millot, 1948). **A** female (CASENT9001207) habitus, lateral view, image reversed **B–C** female (CASENT9001207) internal genitalia **B** dorsal view **C** anterior view, arrows showing two sclerotized invaginations on the bursa **D–K** male pedipalpal bulbs (CASENT9001207), **D–F, K** right bulb, image reversed **G–J** left bulb **D, G, J** prolateral view **E, H** ventral view **F, I** retrolateral view **K** apical view **L** coxae **I** male (CASENT9001207), ventral view, arrows showing the pointed extensions on coxae I. Scale bars: 1 mm (**A**); 0.125 mm (**B**); 0.25 mm (**D**).

**Figure 14.**
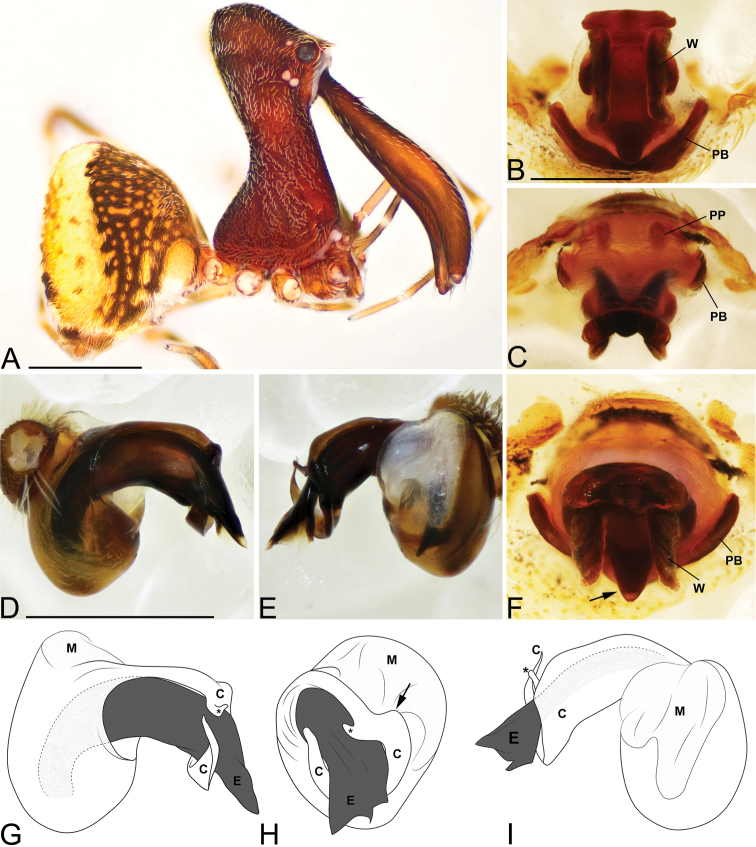
*Eriauchenius lukemacaulayi* sp. n. **A** female (USNMENT01377191) habitus, lateral view, image reversed **B–C, F** female internal genitalia (USNMENT01377192) **B** posterior view **C** anterior view **F** dorsal view, arrow showing the rounded posterior elongation on the FSGP that curves dorsally. **D–E, G–I** male pedipalpal bulbs (holotype, USNMENT01377191), right bulb, image reversed **D, G** prolateral view **E, I** retrolateral view **H** ventral view, arrow showing the large bulge that occurs after the constriction in the conductor. Asterisk (*) showing the narrow, lateral process on the conductor. Scale bars: 1 mm (**A**); 0.25 mm (**B**); 0.5 mm (**D**).

**Figure 15.**
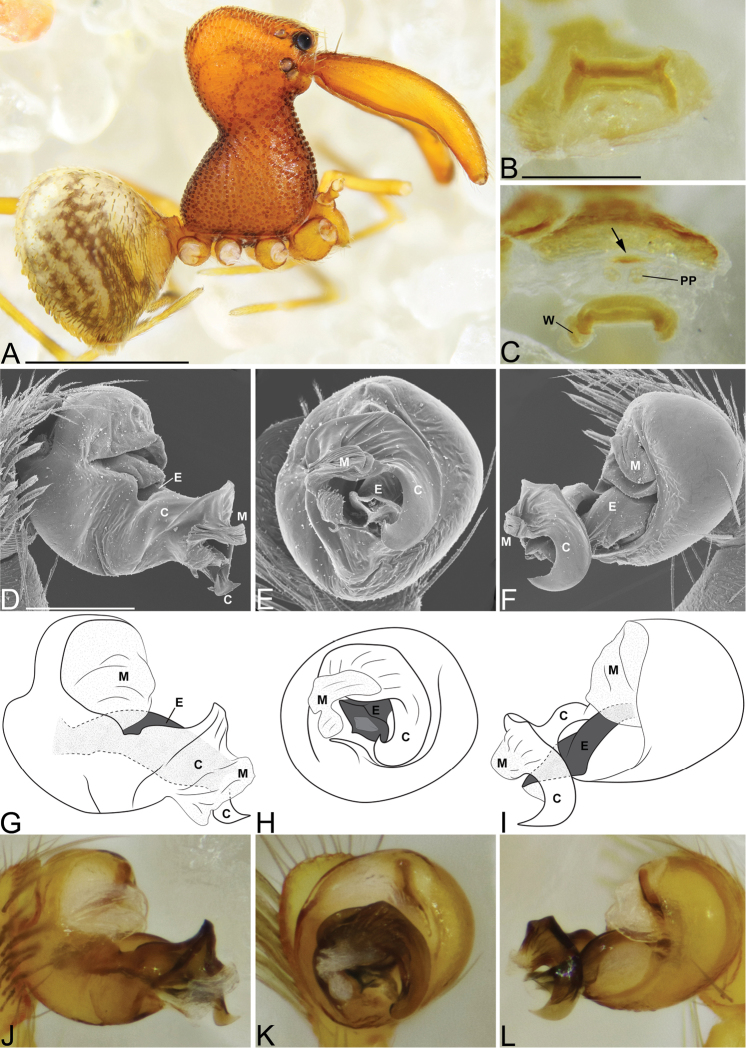
*Eriauchenius mahariraensis* (Lotz, 2003). **A** male (USNMENT01377173) habitus, lateral view, image reversed **B–C** female (holotype, MCZ) internal genitalia **B** dorsal view **C** anterior view, arrow showing the small sclerotized piece next to the poreplates **D–K** male pedipalpal bulbs (USNMENT01377173) **D–F** right bulb, image reversed **G–L** left bulb: **D, G, J** prolateral view **E, H, K** ventral view **F, I, L** retrolateral view. Scale bars: 1 mm (**A**); 0.125 mm (**B, D**)..

**Figure 16.**
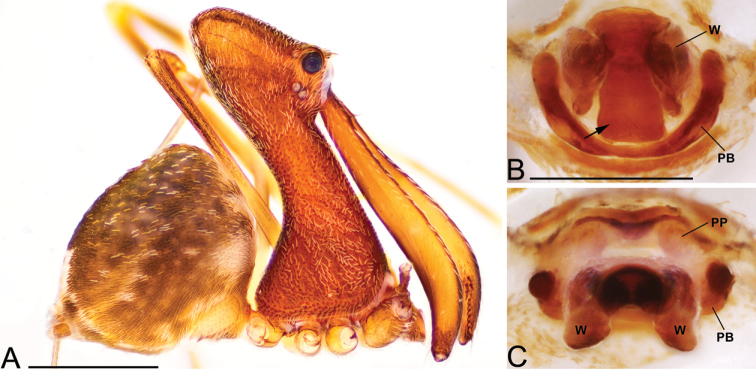
*Eriauchenius milajaneae* sp. n. **A** female (holotype, CASENT9009881) habitus, lateral view **B–C** female (holotype, CASENT9009881) internal genitalia: **B** dorsal view, arrow showing broad, rectangular, posterior elongation on the FSGP **C** anterior view. Scale bars: 1 mm (**A**); 0.25 mm (**B**).

**Figure 17.**
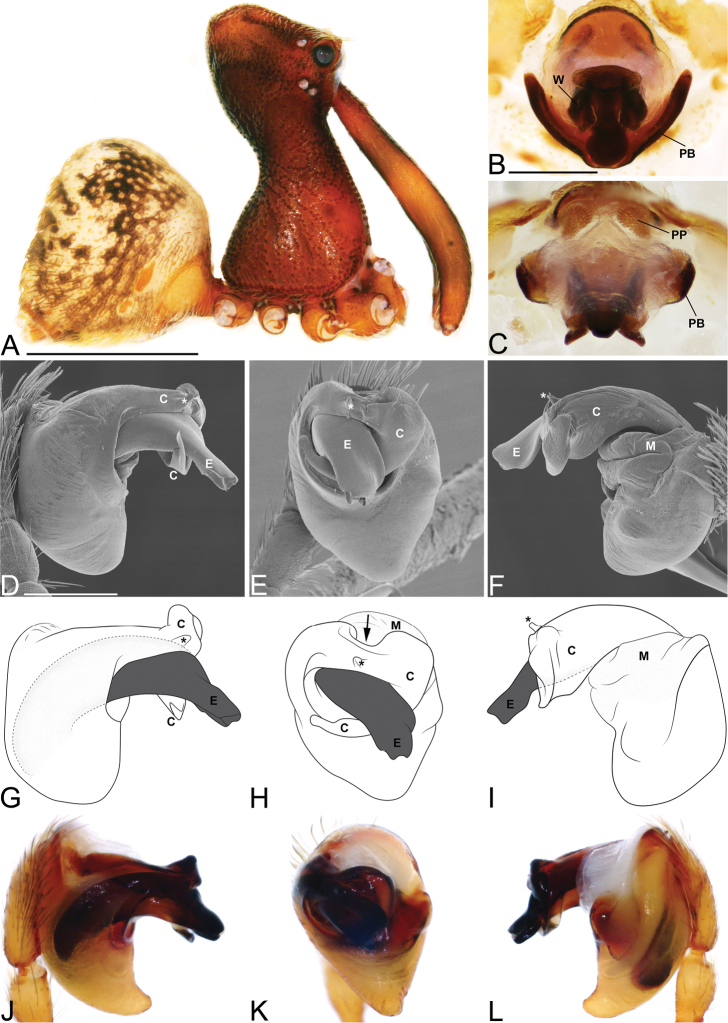
*Eriauchenius milloti* sp. n. **A** male (USNMENT01377189) habitus, lateral view **B–C** female (USNMENT01377189) internal genitalia **B** dorsal view **C** anterior view **D–I** male pedipalpal bulbs **D–F** right bulb, image reversed, (USNMENT01377189) **G–I** left bulb (USNMENT01377189) **J–K** left bulb (CASENT9015862) **D, G, J** prolateral view **E, H, K** ventral view **F, I, L** retrolateral view. Asterisk (*) showing the narrow, small process on the conductor. Arrow showing constriction in the conductor. Scale bars: 1 mm (**A**); 0.25 mm (**B, D**).

**Figure 18.**
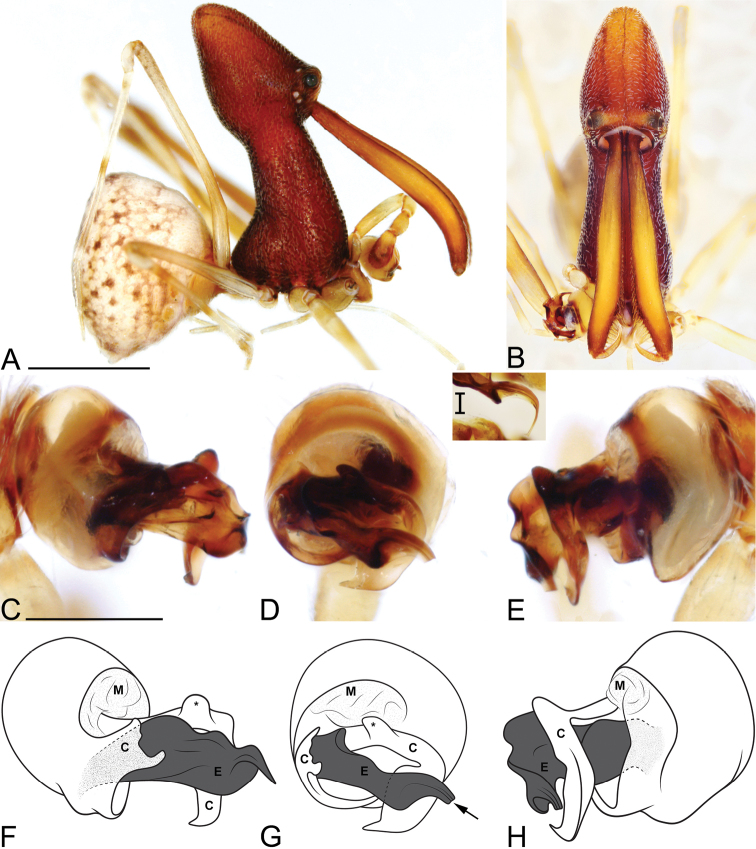
*Eriauchenius pauliani* (Legendre, 1970), holotype, MNHN. **A–B** male **A** habitus, lateral view, image reversed **B** anterior view **C–H** male left pedipalpal bulb **C, F** prolateral view **D, G** ventral view, arrow showing the broken tip of the embolus in the left pedipalp **E, H** retrolateral view **I** male right pedipalpal bulb, embolus tip close-up showing the unbroken embolus, image reversed. Scale bars: 1 mm (**A**); 0.25 mm (**C**).

**Figure 19.**
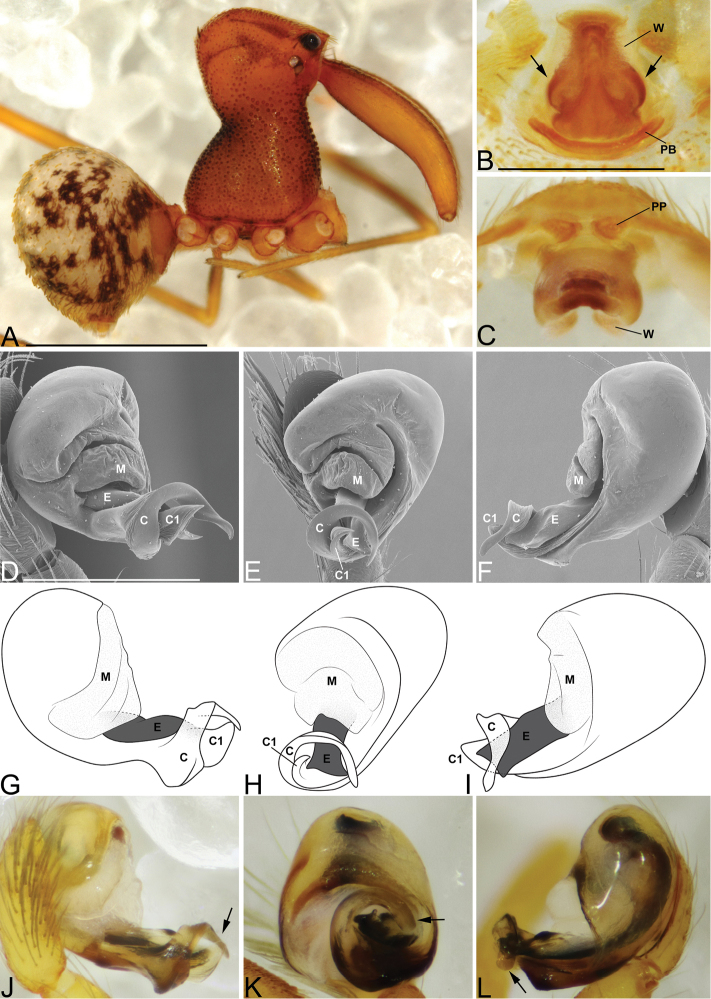
*Eriauchenius ratsirarsoni* Lotz, 2003. **A** male (USNMENT01377174) habitus, lateral view, image reversed **B–C** female (CASENT9028378) internal genitalia **B** posterior-dorsal view, arrows showing the lateral bulges on the FSGP **C** anterior view **D–L** male pedipalpal bulbs **D–F** right bulb (USNMENT01377174), image reversed **G–J** left bulb (USNMENT01377174) **K–L** left bulb (CASENT9062876) **D, G, J** prolateral view **E, H, K** ventral view **F, I, L** retrolateral view **J** arrow showing conductor variation, with a tapering tip **K, L** arrow showing conductor with a blunt tip. Scale bars: 1 mm (**A**); 0.25 mm (**B, D**).

**Figure 20.**
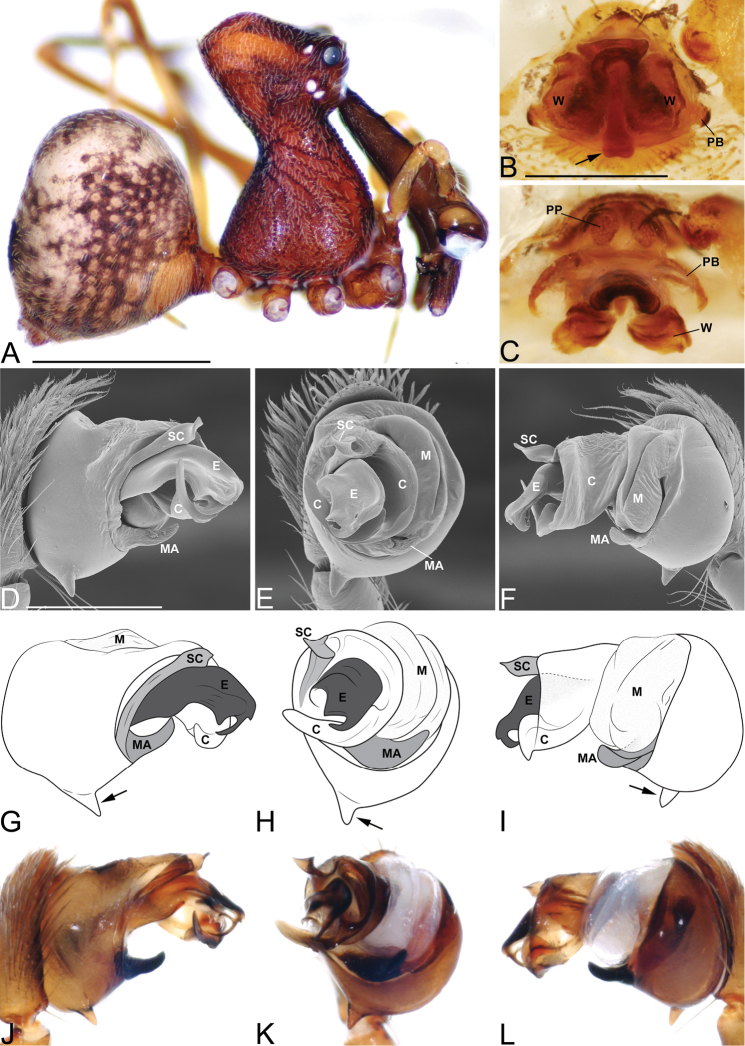
*Eriauchenius rixi* sp. n. **A** male (holotype, CASENT9012013) habitus, lateral view, image reversed **B–C** female (CASENT9012346) internal genitalia **B** dorsal view, arrow showing the FSGP narrow posterior elongation **C** anterior view **D–L** male pedipalpal bulbs (holotype, CASENT9012013) **D–F** right bulb, image reversed **G–L** left bulb, arrow showing the sharp process on the tegulum base **D, G, J** prolateral view **E, H, K** ventral view **F, I, L** retrolateral view. Scale bars: 1 mm (**A**); 0.25 mm (**B, D**).

**Figure 21.**
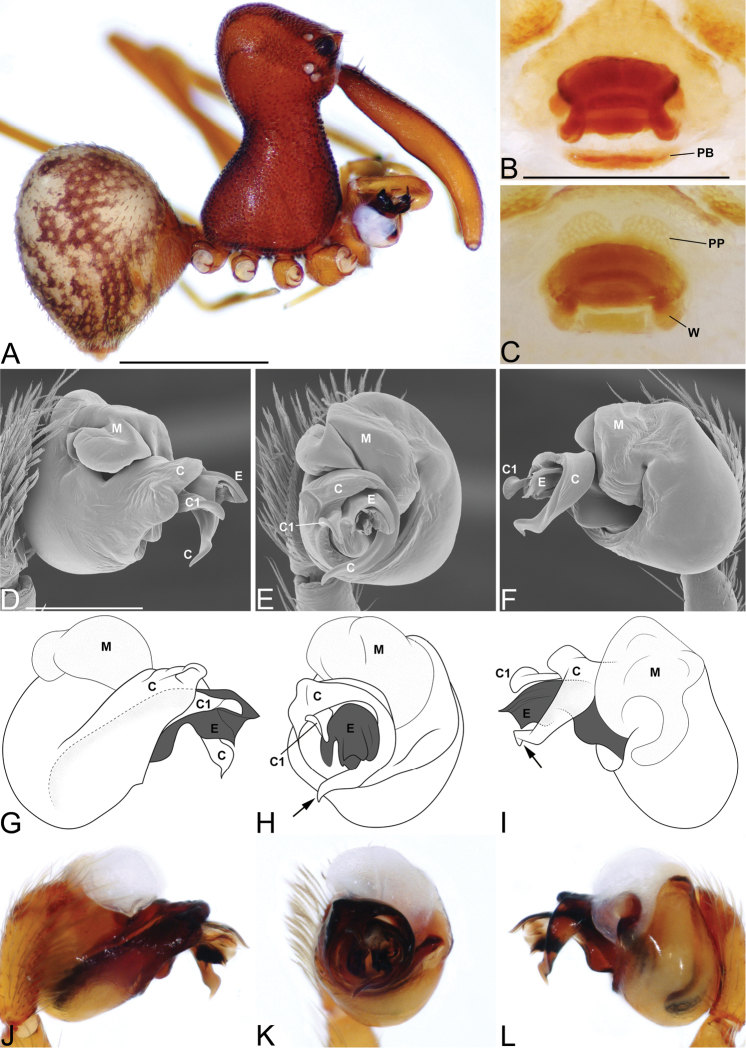
*Eriauchenius sama* sp. n. **A** male (holotype, CASENT9012014) habitus, lateral view, image reversed **B–C** female (CASENT9012341) internal genitalia: **B** dorsal view **C** anterior view **D–L** male pedipalpal bulbs (holotype, CASENT9012014), arrow showing the change in direction in the curve of the conductor tip **D–F** right bulb, image reversed **G–L** left bulb **D, G, J** prolateral view **E, H, K** ventral view **F, I, L** retrolateral view. Scale bars: 1 mm (**A**); 0.25 mm (**B, D**).

**Figure 22.**
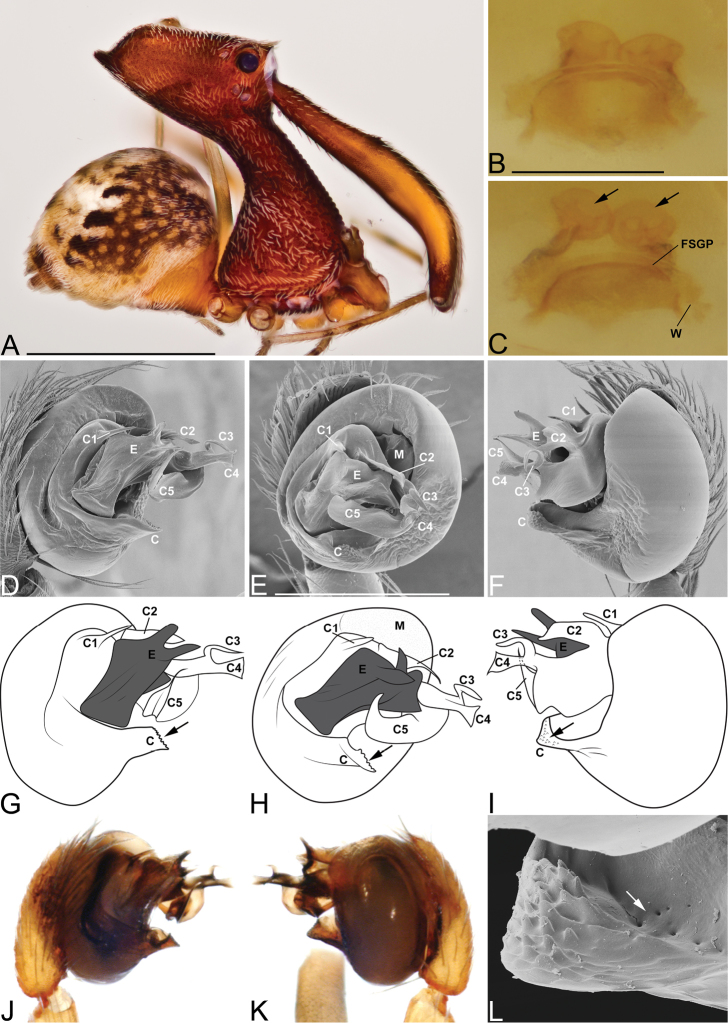
*Eriauchenius zirafy* sp. n. **A** male (CASENT9015571) habitus, lateral view **B–C** female (USNMENT01377190) internal genitalia **B** dorsal view **C** anterior view, arrows showing two sclerotized invaginations on the bursa **D–L** male pedipalpal bulbs (CASENT9015571), black arrow showing the small bumps on the conductor base **D–F, L** right bulb, image reversed **G–K** left bulb: **D, G, J** prolateral view **E, H** ventral view **F, I, K** retrolateral view **L** close-up of small bumps on conductor base, retrolateral view, white arrow showing small pores. Scale bars: 1 mm (**A**); 0.125 mm (**B**); 0.25 mm (**E**).

Abdomen rounded in the “bourgini” (Figs 9–22) or triangular in the “workmani group” (Figs 3–8) due to a single dorsal protuberance, which can be small-to-large; containing numerous small, round, pale indentations throughout and with dark brown to purplish pigment throughout; covered in white-to-brown, thick setae; epigynum and booklung covers flat, sclerotized plates, which are not fused in females, but form a single fused epigastric ventral plate in males; abdominal dorsal plate with ridges (see fig. 5B in Wood 2008), sometimes fused with epigastric plate only in males; dorsal plate sometime extends along the anterior face of the abdomen (scutum) in males only; dorsal plate is always confined to the abdominal petiole region in females. Posterior respiratory system with two spiracular openings (see fig. 5C in Wood 2008).

Spinnerets surrounded by ring; rudimentary-to-fleshy colulus present. The following spinneret description is taken from examining published images of *E. workmani* (Griswold et al. 2005: figs 21–22): anterior lateral spinneret (ALS) spinning field divided, with one large and one smaller major ampullate gland (MAP) spigot on the median side and with approximately 42 smaller piriform gland (PI) (fewer in male) spigots on the lateral side. Posterior median spinneret (PMS) of female with one large median minor ampullate gland spigot (mAP), one lateral medium-sized aciniform gland (AC) spigot, and two lateral cylindrical gland (CY) spigots; the male PMS is the same except in lacking the CY. Posterior lateral spinnerets (PLS) with a middle row of five AC spigots; in females only, this row is flanked on the anterior side by two CY spigots and on the posterior side by one CY spigot.

Legs reddish or orangish to light brown, often with dark brown bands throughout, but especially on the tibia; covered sparsely with setae; ratio 1-2-4-3 or 1-4-2-3, typically 1-2-4-3 for species found in vegetation and 1-4-2-3 for species found in forest litter; one or two anterior rows of scopulae present on leg I, sometimes also present on leg II, and sometimes with a posterior row as well; metatarsus III and IV with a ventral cluster of modified hairs; femur IV distinctly curved (see fig. 7D in Wood 2008); femur I length 1.48–6.48 times the length of the carapace. Female palp with single claw.

Male pedipalpal femur, patella, tibia and cymbium without apophyses, however a cluster of spine like setae with enlarged bases occurs on the distal retrolateral side of the femur in some “bourgini group” species. Palpal bulb very diverse in shape, forming an enclosed pit that the conductor wraps around in the “workmani group” (Fig. 8D–L) and the “bourgini group – enclosed embolus group” (Fig. 9D–L), and with the embolus exposed and encircled by the conductor in the “bourgini group – exposed embolus group” (Fig. 13D–K). Embolus is heavily sclerotized, unconcealed, and wide-to-thin in the “bourgini group” and is sclerotized only at the tip and recessed in the “workmani group”. Conductor either a small dark ridged spiral that goes around the apex of the bulb and terminates in a triangular point (Fig. 9J–K), or a larger, wider piece that circles around the embolus, often with processes (Fig. 13D–I). In the “workmani group” the MA is present and is hidden behind the embolus in unexpanded bulbs (Fig. 8D–E, H); in most “bourgini group” species the male pedipalpal bulb has only a conductor and embolus, however, in some species a MA is also present (Figs 9E, H, 11D–I, 12D–F, 20D–I); in all “workmani group” species and in some “bourgini group” species there is an additional sclerite (SC) present on the male pedipalpal bulbs that may be an additional process on the conductor instead of a separate sclerite (Figs 8D–E, G–H, 9E–I).

Female genitalic bursa height in “workmani group” greater than bursa width (Fig. 8B), and in “bourgini group” less than or equal to bursa width (Fig. 11B); bursa with secretory poreplates with pores distributed in a small continuous group (Fig. 17C) or in a large discontinuous clumpy group on each side (Fig. 8B), although poreplates are absent in *E. bourgini* (Millot, 1948) and *E. zirafy* sp. n. that instead have a sclerotized invagination on either side of the bursa (Fig. 13C); with a dorsal sclerotized plate (FSGP) that can be either a simple arched piece that is wider than long (Fig. 15C) or a more elaborate piece that can have a posterior extension, or points at the top, and that can be longer than wide (Fig. 14B); FSGP in most species with wing-like projections extending to each lateral side that can be reduced-to-large, and that can be heavily sclerotized-to-translucent (Fig. 8B). All species of the “bourgini group” have a large-to-small curved piece that sits posterior to the epigastric furrow, termed the “posterior bar” (Figs 13B, 16B), not present in the “workmani group”. Legendre (1967) suggested that the male palp might come into contact with the FSGP during copulation and that the FSGP may offer tactile information to the male or female. Alternatively, the FSGP may serve as an anchor for muscle attachment (Griswold et al. 2005).

Included species: 6 described species *E. bourgini* (Millot, 1948), *E. fisheri* (Lotz, 2003), *E. mahariraensis* (Lotz, 2003), *E. pauliani* (Legendre, 1970), *E. ratsirarsoni* (Lotz, 2003), *E. workmani* O. Pickard-Cambridge, 1881, and 14 new species described here: *E. andriamanelo* sp. n., *E. andrianampoinimerina* sp. n., *E. goodmani* sp. n., *E. harveyi* sp. n., *E. lukemacaulayi* sp. n., *E. milajaneae* sp. n., *E. milloti* sp. n., *E. rafohy* sp. n., *E. ranavalona* sp. n., *E. rangita* sp. n., *E. rixi* sp. n., *E. sama* sp. n., *E. wunderlichi* sp. n., *E. zirafy* sp. n. One species originally described as *Archaea*, with the extant members later revived as *Eriauchenius*, has been transferred to *Afrarchaea*: *A. cornutus* (Lotz, 2003) (new combination).

#### Distribution.

Madagascar.

#### Discussion.

*Eriauchenius* contains two main clades, the “workmani group” and the “bourgini group” (Fig. 1). The “workmani group” is distinctive: the abdomen has a single tubercle making it triangular in shape (Fig. 8A), whereas the “bourgini group” has a rounded abdomen (Fig. 9A); the “workmani group” also has a highly elongated, constricted “neck” (Fig. 8A). Furthermore, in the “workmani group” the bursa height is greater than the bursa width (Fig. 8B), whereas in the “bourgini group” the bursa height is less than or equal to the bursa width and there is a posterior bar on the FSGP (Fig. 11B–C). The “workmani group” species are bigger in general than other Madagascan archaeid species, ranging in body length from 3.24–6.72.

The “bourgini group” is further broken up into two groups, the “enclosed embolus group” and the “exposed embolus group.” Unfortunately, specimens from the “enclosed embolus group” were not included in the phylogeny of Wood et al (2015), so it is currently unknown whether this group is monophyletic and what their phylogenetic relationship is to other archaeids. The “embolus enclosed group” contains four species (*E. fisheri*, *E. goodmani* sp. n., *E. harveyi* sp. n., *E. sama* sp. n.) that share the following traits: the male pedipalpal bulb is enclosed (Figs 9D–L, 10D–F, 11D–K, 12D–F), the female bursa is sclerotized and covered in pores on the ventral side (Figs 9C, 10C, 11C, 12C) as opposed to the anterior or dorsal side in other *Eriauchenius*, and the AME are virtually flush with the cuticle (Fig. 11L), rather than being on bulges. In the “exposed embolus group” the male pedipalpal bulbs have a more open form, with the embolus exposed and encircled by the elongated conductor, and there is also a membraneous sac on the bulb that is adjacent to the embolus base (Fig. 17D–L). Typical “bourgini group” pedipalpal bulbs have only an embolus and a conductor, and no other sclerites, the exceptions are *E. fisheri*, *E. harveyi* sp. n., *E. rixi* sp. n., and *E. wunderlichi* sp. n., which also have a MA and SC, and *E. goodmani* sp. n., which also has a SC.

*E. fisheri* and *E. mahariraensis* were transferred to *Eriauchenius* from *Afrarchaea* because they lack the FSGP keel observed in *Afrarchaea* and because the FSGP has a posterior bar. *E. mahariraensis* was included in the phylogenetic analysis of Wood et al (2015) and fell inside *Eriauchenius* with a branch support posterior probability value of 1.0. Although, *E. fisheri* was not including in this phylogenetic analysis, the morphological evidence supports this transfer.

### Key for *Eriauchenius*

**Table:** 

1	In males and females, abdomen triangular, with a single tubercule on the dorsal side (Fig. 8A); in females, genitalia with bursa height greater than bursa length, posterior bar absent (Fig. 8B)	**2, “workmani group**”
–	Abdomen rounded in both sexes, without a tubercle (Fig. 11A); in females, bursa height less than or equal to bursa width, posterior bar present (Fig. 11B–C)	**11, “bourgini group**”
“**Workmani-group**”		
2	Male	**3**
–	Female: females are indistinguishable except for *E. andriamanelo* sp. n., *E. andrianampoinimerina* sp. n., and *E. ranavalona* sp. n.	**8**
3	Conductor split (Fig. 3K)	***E. andriamanelo***
–	Conductor triangular (Fig. 8H)	**4**
4	Anterior side of the pedipalpal bulb with a large bump (Fig. 8D–K)	***E. workmani***
–	Anterior side of pedipalpal bulb smooth or only slightly rounded (Fig. 3D–K)	**5**
5	Abdomen with green patches on the lateral and posterior sides (sometimes faded in preserved specimens) (Fig. 6C–D), median apophysis without bi- or trifurcation (Fig. 6E–F, H–I)	***E. ranavalona***
–	Abdomen lacking the green patches (Fig. 7A), MA with bi- or trifurcation (Figs 7C–E, G–H, 5C, H, L)	**6**
6	Median apophysis with trifurcation: with one deep bifurcation, and the prolateral piece of that bifurcation with an additional shallow bifurcation (Fig. 7C–E, G–H)	***E. rangita***
–	MA with bifurcation in median apophysis (Figs 4C–E, 5C, H, L)	**7**
7	Abdomen pattern typical (Fig. 5A), carapace usually tilted less than 80° (Fig. 5A)	***E. rafohy***
–	Abdomen pattern distinctive with undulating brown ‘rings’ (Fig. 4A), carapace more upright, tilted at least 80° (Fig. 4A)	***E. andrianampoinimerina***
8	Bursa in posterior view with sclerotized projection that forms a “T” shape (Fig. 3B, L)	***E. andriamanelo***
–	Bursa in posterior view with a membranous projection that is not “T” shaped (Fig. 7L)	**9**
9	Abdomen with green patches on the lateral and posterior sides (sometimes faded in preserved specimens) (Fig. 6C–D)	***E. ranavalona***
–	Abdomen pattern usually distinctive with undulating brown ‘rings’ (Fig. 4A), carapace more upright, tilted at least 80° (Fig. 4A)	***E. andrianampoinimerina***
“**Bourgini-group**”		
11	Male pedipalpal bulbs with apical conductor encircling a pit-like cavity (Figs 9D–K, 12D–F); conductor terminates in two pieces on the retrolateral side, one piece rounded and the other piece tapering off to a point (except blunt in *E. goodmani* sp. n.) (Figs 9D–I, 12F); in females, the ventral side of the bursa is sclerotized and covered in pores (Figs 9C, 10C, 11C, 12C) as opposed to the anterior side	**12, “enclosed embolus group**”		
–	Male pedipalpal bulbs with a large embolus, encircled by conductor, with a white membraneous sac of cuticle that sits close to the base of the embolus (Figs 13D–J, 17D–L, 19D–L, 20D–L); conductor is large and may have projections (Figs 13D–K, 19D–I); in females, the secretory pores are in two groups on the anterior side of the bursa (Figs 17C, 19C) or there are two sclerotized invaginations on the anterior side of the bursa (Figs 13C, 22B–C)	**15, “exposed embolus group**”		
“**Enclosed embolus group**”		
12	In males, embolus is long and narrow, wire-like (Figs 9E, G–H, L, 11D–I); in females, “wings” on FSGP are reduced (Figs 9B, 11B)	**13**
–	In males, embolus is wide (Figs 10D–E, 12D–E); in females, “wings” on FSGP not reduced, but nearly translucent (Figs 10B, 12B)	**14**
13	In males, embolus broadly curved (Fig. 11E–K); females of *E. harveyi* and *E. fisheri* are indistinguishable	***E. harveyi***
–	In males, embolus tip with two curves making an “s” shape (Fig. 9E, H, K–L)	***E. fisheri***
14	In male pedipalpal bulb, the apical portion of the tegulum, where the conductor swirls around, is elongated so that the bulb is almost twice as long as it is wide (Fig. 12D, F); SC sclerite as wide and nearly as long as embolus (Fig. 12D–E); in females, posterior bar on genitalia has a large bulge in the center (Fig. 12B)	***E. wunderlichi***
–	In males, apical portion of the tegulum not as elongated (Fig. 10D, F); embolus wider and longer than SC sclerite (Fig. 10D–E); in females, posterior bar on genitalia lacking large bulge in the center (Fig. 10B)	***E. goodmani***
“**Exposed embolus group**”		
15	In both males and females, posterior pair of spines on the apex of the cephalon on large protrusions (Figs 13A, 22A)	**16**
–	Posterior spines on cephalon not on large protrusions (Fig. 17A)	**17**
16	In males and females, coxa I with pointed protrusions (Fig. 13L); in male pedipalpal bulbs, basal triangular piece of conductor smooth, lacking numerous small bumps and pores	***E. bourgini***
–	Coxa I rounded and without pointed protrusions in both sexes; in males, basal triangular piece of conductor with numerous small bumps and pores (Fig. 22G–I,L)	***E. zirafy***
17	Sternum completely fused to carapace, cephalon triangular in shape (Fig. 18A; we only examined the male holotype so it is unknown whether this trait is only in males or is in both sexes)	***E. pauliani***
–	In males and females, sternum not fused to carapace; cephalon not triangular in shape (Figs 17A, 20A)	**18**
18	In males, sharp point on posterior side of pedipalpal tegulum (Fig. 20D–L), and median apophysis dark and shaped like a horn (Fig. 20G–J, L)); in female genitalia, posterior bar curves dorsally when observed in anterior view (Fig. 20C)	***E. rixi***
–	Posterior side of male pedipalpal tegulum rounded (Fig. 21D–L) or forming a half-moon shape (Fig. 17J–L), but lacking a sharp point on the pedipalpal tegulum, and MA absent (Fig. 21D–L); in female genitalia, posterior bar curves towards the anterior when observed in the dorsal view (Fig. 17B), sometimes only slightly (Fig. 21B)	**19**
19	On male pedipalpal bulb, conductor with distal membranous region (Fig. 15D–L); in females, FSGP very short and wide (Fig. 15B), with reduced wings (Fig. 15C)	***E. mahariraensis***
–	Conductor lacking distal membranous region (Figs 14G–I, 17D–L, 19D–L, 21D–L); FSGP taller (Figs 14B, 16B, 17B, 21B), with “wings” reduced or not reduced	**20**
20	In males and females, cheliceral seta projecting perpendicular to the cheliceral cuticle (Fig. 21A); in males, conductor with large process on prolateral side (Figs 19D–L, 21D–L)	**21**
–	In both sexes, cheliceral seta downward pointing (Figs 16A, 17A); in males, conductor lacking large process on prolateral side (Figs 17D–L)	**22**
21	In males, pedipalpal bulb shape with the embolus and conductor originating from the posterior portion of the bulb (Fig. 19D–L); in females, FSGP with two lateral bulges (Fig. 19B)	***E. ratsirarsoni***
–	Male pedipalpal bulb shape with the embolus and conductor originating from the center of the bulb (Fig. 21D–L); FSGP lacking two lateral bulges (Fig. 21B)	***E. sama***
22	Male pedipalpal bulb posterior half-moon shaped, although there is variation in degree (compare tegulum shape in Fig. 17J–L with Fig. 17D–I), and constriction on conductor with a ridge (Fig. 17H); in females, posterior bar reaches the anterior edge of the FSGP (Fig. 17B)	***E. milloti***
–	Posterior edge of male pedipalpal bulb more rounded (Fig. 14D–E, G–I) and lacking a ridge on the conductor constriction (Fig. 14H); in females, posterior bar less than half of the length of the FSGP (Fig. 14B)	***E. lukemacaulayi***
–	Males unknown; in females, FSGP with a broad, rectangular posterior elongation, and with posterior bar more than half, but less than the full length of the FSGP (Fig. 16B)	***E. milajaneae***

### The “workmani group” species

#### Eriauchenius andriamanelo sp. n.

Taxon classificationAnimaliaAraneaeArchaeidae

http://zoobank.org/BB78D667-7AA2-4773-A7E8-39E2453EA9A0

Figs 3 , 29

##### Type material.

Male holotype: Madagascar, Antsiranana Prov., Parc National Montagne d’Ambre, 3.6 km 235° SW of Joffreville, 12°32'4"S, 49°10'46"E, 925 m, 20–26 Jan 2001, montane rainforest, general collection, day. Coll. J.J. Rafanomezantsoa et al. (deposited in CAS; CASENT9002806).

##### Other material examined.

MADAGASCAR: Female paratype, same data as holotype, but collected by L.J. Boutin (CASENT9000729); 1M,1F,1Juv, same data as paratype (CASENT9000728); 1M,1Juv, same data as paratype, but collected by beating and sweeping forest understory; 1M, same data as holotype, but by beating low vegetation, EB17 (CASENT9006679); 8M,2F,2Juv Antsiranana, Parc National Montagne d’Ambre, 2.79 air km NE park entrance, 12°32'S, 49°10'E, 1000 m, 21–30 Nov 1993, forest, J. Coddington, C. Griswold, N. Scharff, S. Larcher, R. Andriamasimanana (CASENT9010067, CASENT9010069, CASENT9046597, CASENT9046588, CASENT9046591, CASENT9046580, CASENT9010070); 1M, Antsiranana, Réserve Spéciale d’Ambre, 3.5 km 235° SW Sakaramy, 12°28'8"S, 49°14'32"E, 325m, 26–31 Jan 2001, L.J. Boutin (CASENT9000786); 1M, Antsiranana, Montagne d’Ambre, 12°30'57"S, 49°11'4"E, 12 Aug 1992, V. & B. Roth (CASENT9010068); 1M,1F,1Juv, Park National Montagne d’Ambre, 1.2 km 184° S Joffreville, 12°31'53.5"S, 49°10'36.8"E, 1000–1200 m, 14–20 Dec 2005, montane rainforest, general collecting day and night, H. Wood, H. Raholiarisendra, J. Rabemahafaly (USNMENT01377255, USNMENT01377256, USNMENT01377257); 1M,1Juv, Antsiranana, Parc National Montagne d’Ambre, 12.2 km 211° SSW Joffreville, 12°35'47"S, 49°9'34"E, 1300 m, 2–7 Feb 2001, montane rainforest, general collecting day, J.J. Rafanomezantsoa et al. (CASENT9004508); 2M,2Juv, Antsiranana, Nosy Be, Réserve Naturelle Intégrale de Lokobe, 6.3 km 112° ESE Hellville, 13°25'10"S, 48°19'52"E, 30 m, 19–24 Mar 2001, rainforest, general collecting night, J.J. Rafanomezantsoa et al. (CASENT9003299); 1F, Antsiranana, Nosy Be, Réserve Naturelle Intégrale de Lokobe, 6.3 km 112° ESE Hellville, 13°25'10"S, 48°19'52"E, 30 m, 19–24 Mar 2001, rainforest, general collecting day, J.J. Rafanomezantsoa et al. (CASENT9003265); 1F,4Juv, Antsiranana, Nosy Be, Réserve Naturelle Intégrale de Lokobe, 6.3 km 112° ESE Hellville, 13°25'10"S, 48°19'52"E, 30 m, 19–24 Mar 2001, rainforest, EC30 beating low vegetation, J.J. Rafanomezantsoa et al. (CASENT9003228); 1M, Antsiranana, Nosy Be, Lokobe Forest, 13°24'58.8"S, 48°18'26.5"E, 11–14 Aug 1992, V & B Roth (CASENT9010071); 1F, Antsiranana, Nosy Be, Reserve Naturelle Integrale de Lokobe, 3.61 km ESE Hellville, 13°24'31.4"S, 48°18'9.8"E, 0–50 m, 9–11 Dec 2005, rainforest, general collecting day and night, two feet off ground on vegetation, H. Wood, H. Raholiarisendra (USNMENT01377258); 1M,3F, Antsiranana, Réserve Spéciale de l’Ankarana, 22.9 km 224° SW Anivorano Nord, 12°54'32"S, 49°6'35"E, 80 m, 10–16 Feb 2001, tropical dry forest, EF28, beating low vegetation, Fisher, Griswold et al. (CASENT9007025, CASENT9007026); 1M,2Juv, Antsiranana, Ampasindava, Forêt d’Ambilanivy, 3.9 km 181°S Ambaliha, 13°47'55"S, 48°9'42"E, 600 m, 4–9 Mar 2001, rainforest, general collecting night, J.J. Rafanomezantsoa et al. (CASENT9002367, CASENT9002369); 1F, Antsiranana, Ampasindava, Forêt d’Ambilanivy, 3.9 km 181°S Ambaliha, 13°47'55"S, 48°9'42"E, 600 m, 4–9 Mar 2001, rainforest, EC30 beating low vegetation, Fisher, Griswold et al. (CASENT9007370); 2M,3F, Antsiranana, Forêt d’Anabohazo, 21.6 km 247° WSW of Maromandia, 14°18'32"S, 47°54'52"E, 120 m, 11–16 Mar 2001, tropical dry forest, EF28, beating low vegetation, Fisher, Griswold et al. (CASENT9007495, CASENT9007494, CASENT9007496); 2F, Antsiranana, Forêt d’Anabohazo, 21.6 km 247° WSW of Maromandia, 14°18'32"S, 47°54'52"E, 120 m, 11–16 Mar 2001, tropical dry forest, general collecting night, J.J. Rafanomezantsoa et al. (CASENT9002561, CASENT9002562); 2M,1F,3Juv, Antsiranana, Forêt d’Anabohazo, 21.6 km 247° WSW of Maromandia, 14°18'32"S, 47°54'52"E, 120 m, 11–16 Mar 2001, tropical dry forest, EF28, beating low vegetation, J.J. Rafanomezantsoa et al. (CASENT9003125); 2M,1F, Antsiranana, Réserve Spéciale de l’Ankarana, 13.6 km 192° SSW Anivorana Nord, 12°51'49"S, 49°13'33"E, 210 m, 16–20 Feb 2001, tropical dry forest, beating low vegetation, Fisher, Griswold et al. (CASENT9001532); 2M,1F, Mahajanga, Parc National d’Ankarafantsika, Forêt de Tsimaloto, 18.3 km 46° NE de Tsaramandroso, 16°13'41"S, 46°8'37"E, 135m, 2– 8 Apr 2001, tropical dry forest, EF28, beating low vegetation, Fisher, Griswold, J.J. Rafanomezantsoa et al. (CASENT9007694, CASENT9002864); 1F, Mahajanga, Parc National d’Ankarafantsika, Ampijoroa Station Forestière, 40 km 306° NW Andranofasika, 16°19'15"S, 46°48'38"E, 130 m, 26 Mar - 1 Apr 2001, tropical dry forest, EF28, beating low vegetation, Fisher, Griswold et al. (CASENT9007562); 1M, Mahajanga, Réserve Spéciale de Bemarivo, 23.8 km 223° SW Besalampy, 16°55'30"S, 44°22'06"E, 30 m, 19–23 Nov 2002, tropical dry forest, general collecting day, Fisher, Griswold et al. (CASENT9017961); 1M,2F,1Juv, same as previous except EF28, beating low vegetation, (CASENT9017958); 3M,2F, same as previous except general collecting night (CASENT9017991); 2Juv, same as previous except general collecting, beating and puffing spiders (CASENT9018002).

##### Etymology.

The specific name is a noun in apposition and commemorates King Andriamanelo, the founder of the Merina Kingdom.

##### Diagnosis.

Males and females are considered part of the “workmani group” based on having a single dorsal protuberance on the abdomen (a triangular shaped abdomen). Males are distinguished from all other species in the “workmani group” by the tip of the conductor, which is divided into two separate sclerotized processes (Fig. 3E, H, K), and by the hook shaped MA (Fig. 3C). Females are distinguished from all other species in the “workmani group” by the presence of a heavily sclerotized “T” shaped structure on the posterior of the bursa (Fig. 3B, L).

##### Description.

Male holotype (CASENT9002806). Total length 4.57, carapace 1.69 long, 1.52 wide. Abdomen 2.61 long, 2.50 high, with a dorsal hump. Carapace tilt angle 81.5°, tilt height (CtH) 4.20, constriction 0.55, head length 1.88, neck length 2.29. CtH divided by carapace length 2.49. Cephalon with AME on a large bulge and 4 post-ocular protrusions on the apex of the cephalon (Fig. 3A), each provided with a short modified spine at the tip. Chelicerae 4.25 long, and with spine 0.72 from base of chelicerae (Fig. 3A). Femur I 10.0 long. Sternum 1.20 long, 0.64 wide. Carapace, chelicerae, sternum and femora I & II reddish dark brown with many white setae. All coxae yellowish brown and legs III & IV yellowish brown, with darker annulations on tibiae and metatarsi. Abdomen mottled brown and beige, with tufts of white setae and white book-lung covers (Fig. 3A). Pedipalpal tegulum of the “workmani group” form, with apical conductor encircling a pit-like cavity (Fig. 3C–K). Conductor tip divided into two separate sclerotized processes (Fig. 3K, arrows). MA relatively short, and hook-shaped (Fig. 3C). Embolus similar to other “workmani group” species, being broad and complex with the sperm duct opening in the middle and sclerotization only at the tip (Fig. 3D–K).

Female paratype (CASENT9003265). Total length 4.61, carapace 1.55 long, 1.37 wide. Abdomen 2.83 long, 3.52 wide, with dorsal hump. Carapace tilt angle 79.8°, tilt height (CtH) 4.21, constriction 0.47, head length 1.52, neck length 2.32. CtH divided by carapace length 2.72. Cephalon as in male. Chelicerae 4.09 long, and with spine 0.57 from base of chelicerae. Femur I 6.59 long. Sternum 1.02 long, 0.55 wide. Colours as in male. Female genitalia with sclerotized plate and bursa pores similar to other “workmani group” species, however, having a “T” shaped sclerotized structure on the posterior of the bursa (Fig. 3B, L).

##### Variation.

Total length 3.98–5.17 (males; n=5), 4.47–5.31 (females; n=5); Carapace length 1.58–1.97 (males; n=5), 1.55–1.90 (females; n=5); Femur I 5.38–6.48 times the length of carapace in males (n=5) and 3.80–4.25 times the length of carapace in females (n=5). CtH divided by carapace length 2.35–2.85 in males (n=5) and 2.13–2.72 in females (n=5). Average femur I length 10.44 (males; n=5), 6.89 (females; n=5).

##### Natural history.

Specimen were collected in montane rainforest, rainforest and tropical dry forest, by beating low vegetation, by beating and sweeping forest understory, and by general collecting day and night. Specimens occur from 30–1300 m in elevation.

##### Distribution.

Northern to central western Madagascar (Fig. 29).

#### Eriauchenius andrianampoinimerina sp. n.

Taxon classificationAnimaliaAraneaeArchaeidae

http://zoobank.org/CD83607C-6BE6-40A4-9314-88E60215B214

Figs 4 , 30

##### Type material.

Male holotype: Madagascar, Toamasina, Montagne d’Anjanaharibe, 18 km 21° NNE Ambinanitelo, 15°11'18"S, 49°36'54"E, el. 470 m., rainforest, EC30 beating low vegetation, 8–12 Mar 2003, C. Griswold, B. Fisher et al. (deposited in CAS; CASENT9018910).

##### Other material examined.

MADAGASCAR: 1M, together with holotype (CASENT9018910); 1F paratype, Toamasina, Montagne d’Anjanaharibe, 18 km21° NNE Ambinanitelo, 15°11'18"S, 49°36'54"E, el. 470 m., rainforest, general collecting night, 8–12 Mar 2003, C. Griswold, B. Fisher et al. (CASENT9018902); 3F, Toamasina, Montagne d’Anjanaharibe, 19.5 km 27° NNE Ambinanitelo, 15°10'42"S, 49°38'06"E, el. 1100 m, 12–16 Mar 2003, montane rainforest, general collecting night, Griswold, Fisher et al. (CASENT9018889); 1M, Taomasina, Parc National Masoala, Ambohitsitondroina, Mt. Ambanizana, 15°34'9.9"S, 50°00'12.3"E, elev. 600–650 m., rainforest, 28 Feb 2003, general coll. night, D. Andriamalala, D. Silva et al. (CASENT 9015518); 1M,1Juv, Taomasina, Parc National Masoala, Ambohitsitondroina Mt., Ambanizana, 15°34'9.9"S, 50°00'12.3"E, 750–800 m., 1 Mar 2003, rainforest, Ludd/raking , D. Andriamalala, D. Silva, et al. (CASENT9015244); 1M, Toamasina, Parc National Masoala, Ambohitsitondroina Mt., Ambanizana, 15°34'9.9"S, 50°00'12.3"E, 700–750 m, 28 Feb 2003, rainforest, beating vegetation, D. Andriamalala, D. Silva, et al. (CASENT9015315); 1F, Toamasina, Parc National Masoala, Ambohitsitondroina Mt., Ambanizana, 15°34'9.9"S, 50°00'12.3"E, 750–800 m, 1 Mar 2003, rainforest, sweeping, D. Andriamalala, D. Silva, et al. (CASENT9015417); 1M, Toamasina, Parc National Masoala, Ambohitsitondroina Mt., Ambanizana, 15°34'9.9"S, 50°00'12.3"E, 600–650 m, 1–2 Mar 2003, rainforest, general collecting night, D. Andriamalala, D. Silva, et al. (CASENT9015371); 1F,1Juv, Toamasina, Parc National Masoala, 2 hour hike from Tompolo, 39 km SE Maroantsetra, 15°41'35.5"S, 49°58'22.5"E, 450 m, primary montane rainforest, beating vegetation, 15–17 Dec 2008, F. Alvarez-Padilla, H. Wood (USNMENT01377253); 1M,1F, Toamasina, Montagne d’Akirindro, 7.6 km 341° NNW Ambinanitelo, 15°17'18"S, 49°32'54"E, 600 m., 17–21 Mar 2003, rainforest, general collecting night, Fisher, Griswold et al. (CASENT 9018892); 1M, Toamasina, Mikira forest, 2.5 hour hike from Andaparaty, 29 km N Maroantsetra, 15°12'2.95"S, 49°36'55.0"E, 195m, 10–12 Dec 2008, primary montane rainforest, beating vegetation, F. Alvarez-Padilla & H. Wood (USNMENT01377259); 2Juv, Toamasina, Mikira forest, 2.5 hour hike from Andaparaty, 29 km N Maroantsetra, 15°12'2.95"S, 49°36'55.0"E, 195m, 10–12 Dec 2008, primary montane rainforest, general collecting, F. Alvarez-Padilla & H. Wood (USNMENT01377252); 1F,1Juv, Toamasina, Mikira forest, 2.5 hour hike from Andaparaty, 29 km N Maroantsetra, 15°12'2.95"S, 49°36'55.0"E, 195m, 10–12 Dec 2008, primary montane rainforest, beating vegetation 5–10 feet above ground, F. Alvarez-Padilla & H. Wood (USNMENT01377250, USNMENT01377251); 1M,3F,3Juv, Antsiranana, Parc National de Marojejy, 25.4 km 30° NNE Andapa, 10.9 km 311° NW Manantenina, 14°26'42"S, 49°44'06"E, 1575 m., 21 Nov 2003, montane rainforest, EB26 general collecting night, B.L. Fisher et al. (CASENT 9018952); 1M,1Juv, Parc National de Marojejy, 25.4 km 30° NNE Andapa, 10.9 km 311° NW Manantenina, 14°26'42"S, 49°44'06"E, 2000 m, 23 Feb 2003, montane shrubland, general collecting night, B.L. Fisher et al. (CASENT9018942); 1M, 1F,5Juv, Antsiranana Prov., Marojejy Res., 8.4 km NNW of Manantenina, 14°26’S, 49°45’E, 700 m., 10–16 Nov 1993, C. Griswold, J. Coddington, N. Scharff, S. Larcher, B. Andriamasimanana (CASENT9010046, CASENT9010056); 1M,2Juv, Antsiranana, Parc National de Marojejy, Manantenina River, 28.0 km 38° NE Andapa, 8.2 km 333° NNW Manantenina, 14°26'12"S, 49°46'30"E, 450 m, 12–15 Nov 2003, rainforest, general collecting day, B.L. Fisher et al. (CASENT9034282).

##### Etymology.

The specific name is a noun in apposition and commemorates King Andrianampoinimerina, who unified the Merina Kingdom.

##### Diagnosis.

Males and females are considered part of the “workmani group" based on having a single dorsal protuberance on the abdomen (a triangular shaped abdomen) (Fig. 4A). Males and females are distinguished from all other species in the “workmani group" by the strong abdomen markings and by the “neck” being very upright, having a tilt angle that is greater than 80° (Fig. 4A). Males are distinguished from all other “workmani group” species except *E. rafohy* sp. n. by having the MA with a bifurcation (Fig. 4C).

##### Description.

Male holotype (CASENT9018910, from Montagne d’Anjanaharibe, Madagascar). Total length 4.32, carapace 1.52 long, 1.29 wide. Abdomen 2.40 long, 1.73 high, with a dorsal hump. Carapace tilt angle 88.1°, tilt height (CtH) 3.94, constriction 0.35, head length 1.32, neck length 2.34. CtH divided by carapace length 2.59. Cephalon with AME on a large bulge and 4 post-ocular protrusions on the apex of the cephalon (Fig. 4A), each provided with a short modified spine at the tip. Chelicerae 3.71 long, and with spine 0.33 from base of chelicerae (Fig. 4A). Femur I 9.58 long. Sternum 1.08 long, 0.50 wide. Carapace, chelicerae, sternum and femora I & II reddish dark brown with many white setae. Legs I & II with lighter annulations close to leg joints. All coxae yellowish brown and legs III & IV yellowish brown, with darker annulations on femora, tibiae and metatarsi. Abdomen with characteristic color pattern of undulating brown ‘rings’, with tufts of white setae and white book-lung covers (Fig. 4A). Pedipalpal tegulum of the “workmani group” form, with apical conductor encircling a pit-like cavity (Fig. 4D–I). Conductor tip is a broad triangular point (Fig. 4D–I) similar to other “workmani group” species except *E. andriamanelo*. MA with a bifurcation (Fig. 4C). Embolus similar to other “workmani group” species, being broad and complex with the sperm duct opening in the middle and sclerotization only at the tip (Fig. 4D–E, G–H).

Female paratype (CASENT9018902). Total length 4.17, carapace 1.56 long, 1.21 wide. Abdomen 2.41 long, 1.74 wide, with dorsal hump. Carapace tilt angle 84.2°, tilt height (CtH) 3.90, constriction 0.36, head length 1.27, neck length 2.27. CtH divided by carapace length 2.50. Cephalon as in male. Chelicerae 3.64 long, and with spine 0.38 from base of chelicerae. Femur I 6.81 long. Sternum 0.95 long, 0.47 wide. Colors as in male. Female internal genitalia indistinguishable from other “workmani group” species (Fig. 4B).

##### Variation.

Total length 3.24–4.32 (males; n=5), 3.50–4.67 (females; n=5); Carapace length 1.16–1.46 (males; n=5), 1.36–1.86 (females; n=5); Femur I 6.17–6.39 times the length of carapace in males (n=5) and 3.71–4.35 times the length of carapace in females (n=5). CtH divided by carapace length 2.36–2.59 in males (n=5), 2.13–2.49 in females (n=5). Average femur I length 8.70 (males; n=5), 6.27 (females; n=5).

##### Natural history.

Specimens were collected in montane rainforest, rainforest, and montane shrubland, by beating low vegetation, beating vegetation, sweeping, raking, and by general collecting day and night. Specimens were collected from 195–2000 m in elevation.

##### Distribution.

Northeastern Madagascar (Fig. 30).

#### Eriauchenius rafohy sp. n.

Taxon classificationAnimaliaAraneaeArchaeidae

http://zoobank.org/2456E491-D12F-4355-91EA-E100B16D9D5E

Figs 5 , 30

##### Type material.

Male holotype: Madagascar, Antananarivo, Réserve Spéciale d’Ambohitantely, Forêt d’Ambohitantely, 20.9 km 72° NE Ankazobe, 18°13'31"S, 47°17'13"E, 1410 m., 17–22 Apr 2001, montane rainforest, general collecting night, J.J. Rafanomezantsoa et al. (deposited in CAS; CASENT9006503).

##### Other material examined.

MADAGASCAR: 3F, together with the holotype (CASENT 9006503); Paratype female,1M, 1 hatched eggcase, Antananarivo, Réserve Spéciale d’Ambohitantely, Forêt d’Ambohitantely, 20.9 km 72° NE Ankazobe, 18°13'30.3"S, 47°16'44"E, 1574 m., 19 Mar 2003, primary montane rainforest, Ldd fallen logs/litter (among fallen logs and litter), D. Andriamalala, D. Silva, et al. (CASENT 9015039); 1M,1Juv, Antananarivo, Réserve Spéciale d’Ambohitantely, Forêt d’Ambohitantely, 20.9 km 72° NE Ankazobe, 18°13'30.3"S, 47°16'44"E, 1574 m, 20 Mar 2003, primary montane rainforest, Ludd/raking, D. Andriamalala, D. Silva, et al. (CASENT9015019); 3M, 3F, >70Juvs, Antananarivo, 3 km 41° NE Andranomay, 11.5 km 147° SSE Anjozorobe, 18°28'24"S, 47°57'36"E, 1300 m., 5–13 Dec 2000, montane rainforest, general collecting, Fisher, Griswold et al. (CASENT9004087, CASENT9004076); 2F,6Juv, Antananarivo, 3 km 41° NE Andranomay, 11.5 km 147° SSE Anjozorobe, 18°28'24"S, 47°57'36"E, 1300 m., 5–13 Dec 2000, montane rainforest, beating low vegetation, Fisher, Griswold et al. (CASENT9003844); 1F,8Juv, Antananarivo, 3 km 41° NE Andranomay, 11.5 km 147° SSE Anjozorobe, 18°28'24"S, 47°57'36"E, 1300 m., 5–13 Dec 2000, montane rainforest, beating and sweeping, Fisher, Griswold et al. (CASENT9004010); 1F,1Juv, Antananarivo, 3 km 41° NE Andranomay, 11.5 km 147° SSE Anjozorobe, 18°28'24"S, 47°57'36"E, 1300 m., 5–13 Dec 2000, montane rainforest, cryptic searching, Fisher, Griswold et al. (CASENT9008673); 1F, 1Juv, Antananarivo, Réserve Spéciale d’Ambohitantely, Forêt d’Ambohitantely, 20.9 km 72° NE Ankazobe, 18°13'31"S, 47°17'13"E, 1410 m., 17–22 Apr 2001, montane rainforest, EB17 beating low vegetation, Fisher, Griswold et al. (CASENT9001208).

##### Etymology.

The specific name is a noun in apposition and commemorates Queen Rafohy.

##### Diagnosis.

Males and females are considered part of the “workmani group” based on having a single dorsal protuberance on the abdomen (a triangular shaped abdomen) (Fig. 5A). Males are distinguished from the “workmani group” species *E. andriamanelo* sp. n., *E. ranavalona* sp. n., and *E. rangita* sp. n. by having a bifurcating MA (Fig. 5C), from *E. andrianampoinimerina* by lacking the distinctive abdomen markings (Fig. 5A), and *E. workmani* by lacking the large bump on the pedipalpal bulbs (Fig. 5D–K). Females are distinguished from *E. andriamanelo* by lacking the heavily sclerotized “T” shaped structure on the posterior of the bursa, from *E. andrianampoinimerina* by lacking the strong abdomen markings and by the “neck” having a tilt angle that is less than 80°, and from *E. ranavalona* by lacking the lime-green abdomen markings. Females are indistinguishable from the remaining “workmani group” species.

##### Description.

Male holotype (CASENT9006503, from Réserve Spéciale d’Ambohitantely, Madagascar). Total length 4.41, carapace 1.75 long, 1.29 wide. Abdomen 2.57 long, 1.29 wide, 2.91high, with a prominent dorsal hump. Carapace tilt angle 74.82°, tilt height (CtH) 3.93, constriction 0.56, head length 1.38, neck length 2.29. CtH divided by carapace length 2.25. Cephalon with AME on a large bulge and 4 post-ocular protrusions on the apex of the cephalon (Fig. 5A), each provided with a short modified spine at the tip. Chelicerae 4.02 long, and with spine 0.39 from base of chelicerae (Fig. 5A). Femur I 9.02 long. Sternum 1.12 long, 0.67 wide. Carapace, chelicerae, sternum and femora I & II reddish dark brown with many white setae. All coxae and legs III & IV yellowish brown. The latter with darker annulations on femora, tibiae and metatarsi. Abdomen yellowish brown, mottled with brown, and light brown book-lung covers, all covered with many white setae (Fig. 5A). Pedipalpal tegulum of the “workmani group” form, with apical conductor encircling a pit-like cavity (Fig. 5C–L). Conductor tip is a broad triangular point similar to other “workmani group” species except *E. andriamanelo* sp. n. (Fig. 5D–L). MA with a bifurcation (Fig. 5C, H, L). Embolus similar to other “workmani group” species, being broad and complex with the sperm duct opening in the middle and sclerotization only at the tip (Fig. 5D–E, G–H, J–K).

Female paratype (CASENT9015039). Total length 4.60, carapace 1.69 long, 1.51 wide. Abdomen 2.65 long, 2.39 wide, 3.91 high, with dorsal hump. Carapace tilt angle 73.3°, tilt height (CtH) 4.00, constriction 0.59, head length 1.54, neck length 2.26. CtH divided by carapace length 2.37. Cephalon as in male. Chelicerae 3.99 long, and with spine 0.44 from base of chelicerae. Tarsus of pedipalps with ventral patch of long thick setae. Femur I 6.30 long. Sternum 1.10 long, 0.66 wide. Colors as in male, but generally darker. Female internal genitalia indistinguishable from other “workmani group” species (Fig. 5B).

##### Variation.

Total length 3.39–4.73 (males; n=4), 4.32–4.86 (females; n=6); Carapace length 1.50–1.80 (males; n=4), 1.57–1.75 (females; n=6); Femur I 5.10–5.72 times the length of carapace in males (n=4) and 3.48–3.88 times the length of carapace in females (n=6). CtH divided by carapace length 2.17–2.35 in males (n=4), 2.20–2.39 in females (n=6). Average femur I length 8.70 (males; n=4), 6.16 (females; n=6).

##### Natural history.

Specimens have been collected in montane rainforest through general collecting, beating vegetation, sweeping, raking, cryptic searching, and among fallen logs and litter in altitudes from 1300–1638 m above sea level. One specimen was collected with a hatched eggsac.

##### Distribution.

Known only from Antananarivo Province in central Madagascar (Fig. 30).

#### Eriauchenius ranavalona sp. n.

Taxon classificationAnimaliaAraneaeArchaeidae

http://zoobank.org/DC825D0D-2431-4E45-9898-A37D53542479

Figs 6 , 30

##### Type material.

Male holotype: Madagascar, Fianarantsoa, Parc National Ranomafana, Vohiparara, Piste Touristique, 21°13.6'S, 47°24.0'E, 1000 m., 26–27 Apr 1998, C. Griswold, D. Kavanaugh, N. Penny, M. Raherilalao, E. Rajeriarison, J. Ranorianarisoa, J. Schweikert, D. Ubick (deposited in CAS; CASENT9010047).

##### Other material examined.

MADAGASCAR, Fianarantsoa, Parc National Ranomafana: Female paratype, Vatoharanana River, 4.1 km 231° SW Ranomafana, 21°17'24"S, 47°26'00"E, 1100 m., 27–31 Mar 2003, Griswold, Fisher et al., montane rainforest, EB17 beating low vegetation (CASENT9018917); 2M,1F,1Juv, 2.3 km N Vohiparara village, 21°12.8'S, 47°23.0'E, 1100 m., 24–25 Apr 1998, C. Griswold, D. Kavanaugh, N. Penny, M. Raherilalao, E. Rajeriarison, J. Ranorianarisoa, J. Schweikert, D. Ubick (CASENT9010042); 5M,1Juv, Vohiparara, 3.6 km W Ranomafana, 21°14.243’S, 47°23.842’E, 1150 m., 13–14 Jan 2009, primary montane rainforest, general collecting day and night, C. Griswold, A. Saucedo and H. Wood (USNMENT01377245, USNMENT01377246, USNMENT01377247, USNMENT01377254); 1F, Vatoharanana, 4 km S Ranomafana, 21°17'15.0"S, 47°25'38.5"E, 1100 m, 12 Jan 2009, primary montane rainforest, general collecting day, beating vegetation, A. Saucedo & H. Wood (USNMENT01377248); 2M,3F,2Juv, Vohiparara, Sahamalaotra forest, 41.1 km 54° NE Fianarantsoa, 21°14'19.9"S, 47°23'39.2"E, 1200 m, 26 Dec 2005-14 Jan 2006, montane rainforest, beating vegetation: in clumps of dead dry foliage, H. Wood, J. Miller, J.J. Rafonomezantsoa, E. Rajeriarison, V. Andriamananony (USNMENT01377249, USNMENT01377240); 1M, Vohiparara, 3.62 km ENE Ranomafana, 21°14'28.7"S, 47°23'65.3"E, 1137 m, 13 Jan 2009, evergreen secondary rainforest, general collecting, D. Andriamalala, C. Griswold, G. Hormiga, A. Saucedo, N. Scharff and H. Wood (CASENT9048515); 1F, 7 km SW Ranomafana, 1200 m, 23 Oct 1988, W. Steiner, C. Kremen, R. Van Epps (USNMENT00879986).

##### Etymology.

The specific name is a noun in apposition and commemorates Queen Ranavalona III, the last sovereign of the Kingdom of Madagascar before it became a French colony.

##### Diagnosis.

Males and females are considered part of the “workmani group” based on having a single dorsal protuberance on the abdomen (a triangular shaped abdomen) (Fig. 6A). Males and females are distinguished from all other species in the “workmani group” by the lime-green abdomen markings in living specimens (Fig. 6C–D), which is sometimes faded in alcohol preserved specimens, and in males, by the non-bifurcating MA that is broad and tapers to a point (Fig. 6H–I), rather than being hook shaped as seen in *E. andriamanelo* (Fig. 3C).

##### Description.

Male holotype (CALENT9010047, from Parc Nationale Ranomafana, Madagascar). Total length 3.53, carapace 1.48 long, 1.28 wide. Abdomen 1.93 long, 2.23 high, with a dorsal hump. Carapace tilt angle 75.1°, tilt height (CtH) 3.25, constriction 0.44, head length 1.03, neck length 1.83 (Fig. 2). CtH divided by carapace length 2.20. Cephalon with AME on a large bulge and 4 post-ocular protrusions on the apex of the cephalon (Fig. 6A), each provided with a short modified spine at the tip. Chelicerae 3.28 long, and with spine 0.42 from base of chelicerae (Fig. 6A). Femur I 5.76 long. Sternum 0.92 long, 0.55 wide. Carapace, chelicerae, sternum and femora I & II reddish dark brown with many white setae, and lighter brown areas on head, neck and chelicerae. All coxae yellowish brown and legs III & IV yellowish brown, with darker annulations on femora, tibiae and metatarsi. Abdomen mostly mottled brown with tufts of white setae, white book-lungs, and characteristic lime-green posterior-dorsal area in living species (Fig. 6C–D; often faded to yellowish-white in ethanol preserved material). Pedipalpal tegulum of the “workmani group” form, with apical conductor encircling a pit-like cavity (Fig. 6E–J). Conductor tip is a broad triangular point similar to other “workmani group” species except *E. andriamanelo* (Fig. 3K), where the conductor is divided. MA without a bifurcation, broad, and tapering toward the tip (Fig. 6E–F, H–I). Embolus similar to other “workmani group” species, being broad and complex with the sperm duct opening in the middle and sclerotization only at the tip.

Female paratype (CASENT9018917). Total length 3.86, carapace 1.24 long, 1.15 wide. Abdomen 2.20 long, 2.24 wide, with dorsal hump. Carapace tilt angle 67.6°, tilt height (CtH) 2.71, constriction 0.42, head length 1.05, neck length 1.39. CtH divided by carapace length 2.19. Cephalon as in male. Chelicerae 2.62 long, and with spine 0.48 from base of chelicerae. Femur I 4.52 long. Sternum 0.79 long, 0.53 wide. Colors as in male, but abdomen mottled brown and beige instead of just mottled brown. Female abdomen also with posterior-dorsal lemon-green area. Female internal genitalia indistinguishable from other “workmani group” species (Fig. 6B).

##### Variation.

Total length 3.49–3.68 (males; n=5), 3.34–3.86 (females; n=4); Carapace length 1.35–1.43 (males; n=5), 1.24–1.49 (females; n=4); Femur I 5.25–5.65 times the length of carapace in males (n=5) and 3.53–4.10 times the length of carapace in females (n=4). CtH divided by carapace length 2.16–2.26 in males (n=5), 2.01–2.28 in females (n=4). Average femur I length 7.65 (males; n=5), 5.23 (females; n=4).

##### Natural history.

Specimens have been collected in montane rainforest and evergreen secondary rainforest through beating vegetation, including clumps of dead, dry foliage, by beating low vegetation, and general collecting day and night. Specimens were collected from 900–1200 m above sea level.

##### Distribution.

Known only from Ranomafana National Park in southwestern Madagascar (Fig. 30).

#### Eriauchenius rangita sp. n.

Taxon classificationAnimaliaAraneaeArchaeidae

http://zoobank.org/1F79B5E8-2421-44C8-9981-F98BA51D4F7E

Figs 7 , 30

##### Type material.

Male holotype: Madagascar, Antananarivo, NE outskirts Antananarivo, Ambohimanga Village, 27 Jul 1992, cryptic searching, V. & B. Roth (deposited in CAS; CASENT9010053).

##### Other material examined.

MADAGASCAR: Female paratype, Fianarantsoa, Forêt d’Atsirakambiaty, 7.6 km 285° WNW Itremo, 20°35'36"S, 46°33'48"E, 1550 m., 22–26 Jan 2003, montane rainforest, general collecting day, includes one hatched eggcase, Fisher, Griswold et al. (CASENT9005939); 1F, Fianarantsoa, Forêt d’Atsirakambiaty, 7.6 km 285° WNW Itremo, 20°35'36"S, 46°33'48"E, 1550 m., 22–26 Jan 2003, montane rainforest, general collecting day, includes one hatched eggcase, Fisher, Griswold et al. (CASENT9005772); 1F, Fianarantsoa, Forêt d’Atsirakambiaty, 7.6 km 285° WNW Itremo, 20°35'36"S, 46°33'48"E, 1550 m., 22–26 Jan 2003, montane rainforest, general collecting night, includes one hatched eggcase, Fisher, Griswold et al. (CASENT9016773); 1F,1Juv, Fianarantsoa, Forêt d’Atsirakambiaty, 7.6 km 285° WNW Itremo, 20°35'36"S, 46°33'48"E, 1550 m., 22–26 Jan 2003, montane rainforest, general collecting, beating and puffing spiders, Fisher, Griswold et al. (CASENT 9018883); 1F, Fianarantsoa, Forêt d’Atsirakambiaty, 7.6 km 285° WNW Itremo, 20°35'36"S, 46°33'48"E, 1550 m., 24 Jan 2003, montane rainforest, within fallen palm frond, includes one eggcase, Fisher, Griswold et al. (CALENT9005822); 1M,2F, Toliara, Réserve Spéciale d’Ambohijanahary, Forêt d’Ankazotsihitafototra, 35.2 km 312° NW Ambaravaranala, 18°16'00"S, 45°24'24"E, 1050 m., 13–17 Jan 2003, montane rainforest, general collecting night, Fisher, Griswold et al. (CASENT9018159); 1M, Toliara, Réserve Spéciale d’Ambohijanahary, Forêt d’Ankazotsihitafototra, 35.2 km 312° NW Ambaravaranala, 18°16'00"S, 45°24'24"E, 1050 m., 13–17 Jan 2003, montane rainforest, general collecting day, Fisher, Griswold et al. (CASENT9018251).

##### Etymology.

The specific name is a noun in apposition and commemorates Queen Rangita.

##### Diagnosis.

Males and females are considered part of the “workmani group” based on having a single dorsal protuberance on the abdomen (a triangular shaped abdomen) (Fig. 7A). Males are distinguished from all other species in the “workmani group” by the shape of the MA that has two bifurcations, one deep and one shallow (Fig. 7C). Females are distinguished from *E. andriamanelo* by lacking the heavily sclerotized “T” shaped structure on the posterior of the bursa, from *E. andrianampoinimerina* by lacking the strong abdomen markings and by the “neck” having a tilt angle that is less than 80°, and from *E. ranavalona* by lacking the lime-green abdomen markings. Females are indistinguishable from the remaining “workmani group” species.

##### Description.

Male holotype (CASENT9010053, from Ambohimanga Village, Madagascar). Total length 4.15, carapace 1.58 long, 1.37 wide. Abdomen 2.44 long, 2.74 high, with a prominent dorsal hump. Carapace tilt angle 71.52°, tilt height (CtH) 3.64, constriction 0.50, head length 1.50, neck length 2.09. CtH divided by carapace length 2.30. Cephalon with AME on a large bulge and 4 post-ocular protrusions on the apex of the cephalon (Fig. 7A), each provided with a short modified spine at the tip. Chelicerae 3.69 long, with seta 0.52 from base of chelicerae (Fig. 7A). Femur I 6.93 long. Sternum 1.11 long, 0.59 wide. Carapace, chelicerae, sternum, coxae and femora I & II reddish dark brown with many white setae. Legs III & IV yellowish brown, with darker annulations on femora, tibiae and metatarsi. Abdomen yellowish brown, mottled with grayish brown, and light brown book-lung covers, all covered with many white setae (Fig. 7A). Pedipalpal tegulum of the “workmani group” form, with apical conductor encircling a pit-like cavity (Fig. 7D–K). Conductor tip is a broad triangular point similar to other “workmani group” species except *E. andriamanelo* (Fig. 3H), where the conductor is divided. MA with a broad unsclerotized transparent base and two apical sclerotized processes, one forked and the other un-forked, so the MA looks tri-forked in the unexpanded palp (Fig. 7C, G, J–K). Embolus similar to other “workmani group” species, being broad and complex with the sperm duct opening in the middle and sclerotization only at the tip (Fig. 7E, H, K).

Female paratype (CASENT9005939). Total length 4.17, carapace 1.66 long, 1.36 wide. Abdomen 2.32 long, 1.94 wide, 2.52 high, with dorsal hump. Carapace tilt angle 75.0°, tilt height (CtH) 3.55, constriction 0.46, head length 1.43, neck length 2.06 (Fig. 2). CtH divided by carapace length 2.14. Cephalon as in male. Chelicerae 3.52 long, and with spine 0.41 from base of chelicerae. Femur I 7.21 long. Sternum 1.05 long, 0.55 wide. Colors as in male, but abdomen generally darker and without clear pattern. Female internal genitalia indistinguishable from other “workmani group” species (Fig. 7B, L).

##### Variation.

Total length 3.70–4.17 (males; n=3), 4.30–4.84 (females; n=5); Carapace length 1.47–1.66 (males; n=3), 1.63–1.94 (females; n=5); Femur I 4.3–4.39 times the length of carapace in males (n=3) and 3.22–3.55 times the length of carapace in females (n=5). CtH divided by carapace length 2.13–2.31 in males (n=3), 2.19–2.31 in females (n=5). Average femur I length 6.82 (males; n=3), 6.04 (females; n=5).

##### Natural history.

Specimens have been collected in montane rainforest through general collecting day and night, ‘cryptic searching’, and within a fallen palm frond, at altitudes of 1050–1550 m above sea level. Several specimens have been collected with separate eggsacs that are almost the size of the spider and contains 30–40 relatively large white eggsacs wrapped in thin transparent silk. A couple of hatched eggsacs have also been collected.

##### Distribution.

Known only from central Madagascar (Fig. 30).

#### Eriauchenius workmani

Taxon classificationAnimaliaAraneaeArchaeidae

O. Pickard-Cambridge, 1881

Figs 8 , 29

Eriauchenius workmanni O. P.-Cambridge, 1881: 768, pl. 66, fig. 2.
Archaea workmani (O. P.-Cambridge): Simon 1895: 935–937, figs 32, 1005–1006.
Eriauchenius workmanni O. P.-Cambridge: Perez-Gonzalez et al. 2016: 37, fig. 3D.

##### Taxonomical note.

Originally named *Eriauchenius workmanni* by Pickard-Cambridge in 1881, but subsequently referred to as *Eriauchenius workmani*, except for a very recent reference to the original spelling (World Spider Catalogue 2017). Article 33.3.1 of the International Code of Zoological Nomenclature states that “when an incorrect subsequent spelling is in prevailing usage and is attributed to the publication of the original spelling, the subsequent spelling and attribution are to be preserved and the spelling is deemed to be a correct original spelling”. We here follow the World Spider Catalogue and consider the spelling “*workmani*” as “prevailing use” and therefore the correct spelling of this species name.

##### Type material.

Juvenile holotype: Madagascar, T. Workman (examined, deposited in BMNH).

##### Material described.

1M,1F, MADAGASCAR: Antsiranana, Parc National de Marojejy, Manantenina River, 28.0 km 38° NE Andapa, 14°26.2'S, 49°46.5'E, 450 m, 12–15 Nov 2003, rainforest, general collecting night, B.L. Fisher et al. (CASENT9018947).

##### Other material examined.

MADAGASCAR: 2F, Fianarantsoa, Parc National Ranomafana, Vohiparara, 3.6 km W Ranomafana, 21°14.243’S, 47°23.842’E, 1150 m, 13–14 Jan 2009, primary montane rainforest, with prey, general collecting day and night, C. Griswold, A. Saucedo and H. Wood (USNMENT01377241, USNMENT01377242); 3M,2Juv, Toamasina, Station Forestier, Analamazaotra, administered by Mitsinjo, 0.75 km N Andasibe, 18°55.783’S, 48°24.696’E, 964m, 31 Jan - 03 Feb 2009, primary montane rainforest, hand collected at night in vegetation, C. Griswold, A. Saucedo and H. Wood (USNMENT01377243, USNMENT01377244, USNMENT01377235, USNMENT01377236); 8M,1F,1Juv, Antsiranana, Marojejy Reserve, 8.4 km NNW Manantenina, 14°26'S, 49°45'E, 700 m, 10–16 Nov 1993, C. Griswold, J. Coddington, N. Scharff, S. Larcher, R. Andriamasimanana (CASENT9046573, CASENT9046587, CASENT9046604, CASENT9046581, CASENT9046583, CASENT9010048); 2M,6F,5Juv, Toamasina, Parc National Perinet, nr Andasibe, 18°56'S, 48°24'E, 1000 m, 4–5 Nov 1993, J. Coddington, S. Larcher, C. Griswold, R. Andriamasimanana, and N. Scharff (CASENT9010061, CASENT9046574, CASENT9048501); 1M,1F, Toamasina, Ivoloina Parque Zoologique, 12 km from Tamatave, 18°03'21.6"S, 49°21'32.5"E, 26m, 19 Feb 2003, disturbed rainforest, general collecting night, D. Andriamalala, D. Silva, et al. (CASENT9015765); 2M,1F, Toamasina Res., Analamazaotra, Parc National Andasibe, 23 road km E Moramanga, 18°56'38.2"S, 48°25'03.2"E, 960 m, 16–18 Jan 2003, rainforest, general collecting night, C. Griswold, D. Silva, and D. Andriamalala (CASENT9005234); 2M,2F,1Juv, same as previous except beating vegetation (CASENT9005155); 1M, Antsiranana, Forêt de Binara, 91 km 233° SW Daraina, 13°15'48"S, 49°36'12"E, 650–800 m, 3 Dec 2003, rainforest, general collecting day B.L. Fisher et al. (CASENT9018941); 1M,2F,1Juv, Toamasina, Montagne d’Anjanaharibe, 18.0 km 21° NNE Ambinanitelo, 15°11'18"S, 49°36'54"E, 470 m, 8–12 Mar 2003, EC30 beating low vegetation, rainforest, C. Griswold, B.L. Fisher et al. (CASENT9018909); 3M,2Juvs, same as previous except general collecting, beating and puffing spiders (CASENT9018914); 1F, same as previous except general collecting night (CASENT9018903); 1M,1F, Toamasina, Parc National Masoala, Ambohitsitondroina Mt., Ambanizana, 15°34'9.9"S, 50°00'12.3"E, 650 m, 26 Feb-03 Mar 2003, rainforest, general collecting, camp, D. Andriamalala, D. Silva, et al. (CASENT9015605); 1F, Toamasina, Parc National Masoala, Ambohitsitondroina Mt., Ambanizana, 15°34'9.9"S, 50°00'12.3"E, 600–650 m, 28 Feb 2003, rainforest, general collecting night, D. Andriamalala, D. Silva, et al. (CASENT9015519); 2F,1Juv, Toamasina, Parc National Masoala, Ambohitsitondroina Mt., Ambanizana, 15°34'9.9"S, 50°00'12.3"E, 600–650 m, 01–02 Mar 2003, rainforest, general collecting night, D. Andriamalala, D. Silva, et al. (CASENT9015370, CASENT9015132); 1F, Toamasina, Sahafina forest, 11.4 km W Brickaville, 18°48'52"S, 48°57'43"E, 140 m, 13–14 Dec 2007, rainforest, sifted litter, B.L. Fisher et al. (CASENT9028392); 1M,2Juvs, Toamasina, Ambatovy, 18°51'03"S, 48°19'17"E, 1075 m, 23 Mar 2004, montane rainforest, general collecting, B.L. Fisher et al. (CASENT9018977); 1M,1F, Fianarantsoa, Parc National Ranomafana, Vatoharanana River, 4.1 km 231° SW Ranomafana, 21°17'24"S, 47°26'00"E, 1100 m, 27–31 Mar 2003, montane rainforest, EB17 beating low vegetation, C. Griswold, B.L. Fisher et al. (CASENT9018919); 1M, Fianarantsoa, Parc National Ranomafana, Vohiparara, 3.6 km W Ranomafana, 21°14.243’S, 47°23.842’E, 1150 m, 13–14 Jan 2009, primary montane rainforest, hand collected at night in vegetation, C. Griswold, A. Saucedo and H. Wood (USNMENT01377237); 1F, same as previous except general collecting day and night (USNMENT01377238); 1M, Toamasina, Station Forestier Analamazaotra, administered by Mitsinjo, 0.75 km N Andasibe, 18°55.783’S, 48°24.696’E, 964m, 31 Jan-3 Feb 2009, primary montane rainforest, general collecting day and night, C. Griswold, A. Saucedo and H. Wood (USNMENT01377239); 1M,5Juvs, Fianarantsoa, Parc National Ranomafana, Vatoharanana River, 4.1 km 231° SW Ranomafana, 21°17'24"S, 47°26'00"E, 1100 m, 27–31 Mar 2003, general collecting night, montane rainforest, C. Griswold, B.L. Fisher et al. (CASENT9018968); 1F,2Juvs, same as previous except general collecting, beating and puffing spiders (CASENT9018922); 1M,1F,3Juvs, Fianarantsoa, Parc National Ranomafana, Vohiparara, 21°14'S, 47°24'E, 900 m, 5–7 Dec 1993, N. Scharff, S. Larcher, C. Griswold, and R. Andriamasimanana (CASENT9048502, CASENT9010051); 1M,2F, Fianarantsoa, Parc National Ranomafana, Talatakely, 21°14.9'S, 47°25.6'E, 15–18 Apr 1998, C. Griswold, D. Kavanaugh, N. Penny, M. Raherilalao, J. Ranorianarisoa, J. Schweikert, D. Ubick (CASENT9010050); 1M,2F,2Juvs, Fianarantsoa Parc National Ranomafana, Talatakely, 21°15'S, 47°25'E, 5–7 Dec 1993, N. Scharff, S. Larcher, C. Griswold, and R. Andriamasimanana (CASENT9048504); 1M,1F,5Juvs, same as previous except night collecting (CASENT9012326); 1M,1Juv, Toamasina, Montagne d’Akirindro, 7.6 km 341° NNW Ambinanitelo, 15°17'18"S, 49°32'54"E, 600 m, 17–21 Mar 2003, rainforest, EC30 beating low vegetation, B.L. Fisher, C. Griswold et al. (CASENT9018887); 10 Juvs, Fianarantsoa, Parc National Ranomafana, ca. 21°14.3'S, ca. 47°26.0'E, 800 m, 22 Apr 1998, roadside vegetation near park entrance, C. Griswold, D. Kavanaugh, N. Penny, M. Raherilalao, J. Ranorianarisoa, J. Schweikert, D. Ubick (CASENT9048506); 2Juvs, Fianarantsoa, Vohiparara, broken bridge, 21°13.57'S, 47°22.19'E, 3640 ft, 8–15 Nov 2001, rainforest, high altitude, malaise, R. Harin’Hala (CASENT9010479); 1F, Fianarantsoa, Parc National Ranomafana, 2.3 km N Vohiparara village, 21°12.8'S, 47°23.0'E, 1100 m, 24–25 Apr 1998, C. Griswold, D. Kavanaugh, N. Penny, M. Raherilalao, E. Rajeriarison, J. Ranorianarisoa, J. Schweikert, D. Ubick (CASENT9010066); 1Juv, Fianarantsoa, Parc National Ranomafana, 2.3 km N Vohiparara village, 21°12.8'S, 47°23.0'E, 1100 m, 28 Apr 1998, C. Griswold, D. Kavanaugh, N. Penny, M. Raherilalao, E. Rajeriarison, J. Ranorianarisoa, J. Schweikert, D. Ubick (CASENT9048508); 3F, 2 eggcases, Fianarantsoa Parc National Ranomafana, Talatakely, 21°14.9'S, 47°25.6'E, 19–30 Apr 1998, C. Griswold, D. Kavanaugh, N. Penny, M. Raherilalao, J. Ranorianarisoa, J. Schweikert, D. Ubick (CASENT9010063, CASENT9010049, CASENT9010041); 1M, Fianarantsoa, Parc National Ranomafana, Talatakely, near Cascade Riana, 21°14.9'S, 47°25.6'E, 19–30 Apr 1998, C. Griswold, D. Kavanaugh, N. Penny, M. Raherilalao, J. Ranorianarisoa, J. Schweikert, D. Ubick (CASENT9010059); 2F,6Juvs, 1 eggcase, Fianarantsoa, Parc National Ranomafana, Talatakely, 21°14.9'S, 47°25.6'E, 5–18 Apr 1998, C. Griswold, D. Kavanaugh, N. Penny, M. Raherilalao, J. Ranorianarisoa, J. Schweikert, D. Ubick (CASENT9010062, CASENT9010055); 2M, same as previous except collected at night (CASENT9010043); 1M,1F, Fianarantsoa, Parc National Ranomafana, Talatakely, 21°15'S, 47°25'E, 900 m, 5–7 Dec 1993, N. Scharff, S. Larcher, C. Griswold, and R. Andriamasimanana (CASENT9010064); 1F, Fianarantsoa, Parc National Ranomafana, nr. Talatakely, 21°15'S, 47°25'E, 900 m, 7 Dec 1993, C.E. Griswold (CASENT9010045); 1F, Fianarantsoa, 7 km W Ranomafana, 1100 m, 8–21 Oct 1998, montane rainforest, malaise trap in small clearing, W.E. Steiner (CASENT9010054); 1Juv, Fianarantsoa, Parc National Ranomafana, Talatakely, Sakaroa Falls, 21°15'S, 47°26'E, 1050 m, 31 Nov 1998, V.F. lee & K.J. Ribardo (CASENT9048509); 1F, Fianarantsoa, Parc National Ranomafana, Talatakely, Belle Vue Trail, 1000 m, 13–22 Apr 1998, E.I. Schlinger (CASENT9012335); 1M, Fianarantsoa, 7 km W Ranomafana, 1100 m, 22–31 Oct 1998, montane rainforest, pyrethrin fogging of dead leaves on fallen trees, W.E. Steiner (CASENT9010057); 5M, Fianarantsoa, Parc National Ranomafana, Talatakely, 21.25041°S, 47.41945°E, 900 m, 2–22 Jan 2001, mixed tropical forest, D.H. & K.M. Kavanaugh, R.L. Brett, E. Elsom, F. Vargas (CASENT9003490); 1F,2Juvs, Fianarantsoa, Parc National Ranomafana, Vohiparara, 21.24032°S, 47.39399°E, 1150 m, 2–22 Jan 2001, mixed tropical forest, D.H. & K.M. Kavanaugh, R.L. Brett, E. Elsom, F. Vargas (CASENT9003438); 3M,2F, Toamasina, Station Forestier Analamazaotra, administered by Mitsinjo, 0.75 km N Andasibe, 18°55.783’S, 48°24.696’E, 964m, 31 Jan-3 Feb 2009, primary montane rainforest, general collecting day and night, C. Griswold, A. Saucedo and H. Wood (USNMENT01377230, USNMENT01377231, USNMENT01377232); 3M,1F,4Juvs, Fianarantsoa, Parc National Ranomafana, Talatakely, 21°14.9'S, 47°25.6'E, 19–30 Apr 1998, C. Griswold, D. Kavanaugh, N. Penny, M. Raherilalao, J. Ranorianarisoa, J. Schweikert, D. Ubick (CASENT9012324); 2M,1F,2Juvs, Fianarantsoa, Parc National Ranomafana, Talatakely, 21°15'S, 47°25'E, 900 m, 5–7 Dec 1993, N. Scharff, S. Larcher, C. Griswold, and R. Andriamasimanana (CASENT9012325); 1M, Fianarantsoa, Parc National Ranomafana, Vohiparara, Piste Touristique, 21°13.6'S, 47°24.0'E, 1000 m, 16–27 Apr 1998, C. Griswold, D. Kavanaugh, N. Penny, M. Raherilalao, E. Rajeriarison, J. Ranorianarisoa, J. Schweikert, D. Ubick (CASENT9010058); 2M,8Juvs, Fianarantsoa, Parc National Ranomafana, 2.3 km N Vohiparara village, 21°12.8'S, 47°23.0'E, 1100 m, 18 Apr 1998, C. Griswold, D. Kavanaugh, N. Penny, M. Raherilalao, E. Rajeriarison, J. Ranorianarisoa, J. Schweikert, D. Ubick (CASENT9012323); 1M,1Juv, Toamasina, Res. Analamazaotra, Parc National Andasibe, 23 road km E Moramanga, 18°56'38.2"S, 48°25'03.2"E, 960 m, 16–18 Jan 2003, rainforest, general collecting night, C. Griswold, D. Silva, and D. Andriamalala (CASENT9005571); 1M, Fianarantsoa, Parc National Ranomafana, Talatekely forest, 42.3 km 58° NE Fianarantsoa, 21°15'28.0"S, 47°25'21.8"E, 1050 m, 24 Dec 2005–14 Jan 2006, montane rainforest, general collecting, H. Wood, J. Miller, J.J. Rafonomezantsoa, E. Rajeriarison, V. Andriamananony (USNMENT01377233); 1M,2Juvs, Fianarantsoa, Parc National Ranomafana, Vatoharanana, 4 km S Ranomafana, 21°17'15.0"S, 47°25'38.5"E, 1100 m, 12 Jan 2009, primary montane rainforest, general collecting day, beating vegetation, A. Saucedo & H. Wood (USNMENT01377234); 1M, Toamasina, Parc National Masoala, 2 hour hike from Tompolo, 39 km SE Maroantsetra, 15°41'35.5"S, 49°58'22.5"E, 450 m, 15–17 Dec 2008, primary montane rainforest, general collecting day, F. Alvarez-Padilla & H. Wood (USNMENT01377225); 1F, Toliara, Parc National Andohahela, Parcelle I, Manangotry, off Rte. 118, 34 km N Taolagnaro, 24°44'35.0"S, 46°51'23.3"E, 670 m, 23 Dec 2008–3 Jan 2009, primary montane rainforest, general collecting day and night, F. Alvarez-Padilla & H. Wood (USNMENT01377226); 1M, Toamasina, Station Forestier Analamazaotra, administered by Mitsinjo, 0.75 km N Andasibe, 18°55.783'S, 48°24.696'E, 964m, 31 Jan–3 Feb 2009, primary montane rainforest, hand collected at night in vegetation, C. Griswold, A. Saucedo & H. Wood (USNMENT01377227); 1Juv, Fianarantsoa, Parc National Ranomafana, Vohiparara, 3.62 km ENE Ranomafana, 21°14'28.7"S, 47°23'65.3"E, 1137 m, 13 Jan 2009, Evergreen secondary rainforest, general collecting, D. Andriamalala, C. Griswold, G. Hormiga, A. Saucedo, N. Scharff and H. Wood (CASENT9048517); 1M,2F, Toliara, Parc National Andohahela, Parcelle I, Manangotry, off Rte. 118, 34 km N Taolagnaro, 24°44'35.0"S, 46°51'23.3"E, 670 m, 23 Dec 2008–3 Jan 2009, primary montane rainforest, general collecting day and night, F. Alvarez-Padilla & H. Wood (USNMENT01377228); 1M,2F,1 eggcase, same as previous except one female with eggcase found on ground under palm frond, and one specimen caught by beating (USNMENT01377229); 9M,4F,3Juvs, Fianarantsoa, Parc National Ranomafana, Talatekely forest, 42.3 km 58° NE Fianarantsoa, 21°15'28.0"S, 47°25'21.8"E, 1050 m, 24 Dec 2005-14 Jan 2006, montane rainforest, general collecting day and night, H. Wood, J. Miller, J.J. Rafonomezantsoa, E. Rajeriarison & V. Andriamananony (USNMENT01377220, USNMENT01377221, USNMENT01377222, USNMENT01377223, USNMENT01377224); 1F, 1 eggcase hatched in captivity to ~20 juvs, Fianarantsoa, Parc National Ranomafana, Vohiparara, Sahamalaotra forest, 41.1 km 54° NE Fianarantsoa, 21°14'19.9"S, 47°23'39.2"E, 1200 m, 26 Dec 2005-14 Jan 2006, montane rainforest, general collecting day, beating vegetation: in clumps of dead dry foliage, H. Wood, J. Miller, J.J. Rafonomezantsoa, E. Rajeriarison & V. Andriamananony (USNMENT01377215); 1F, 1 eggcase hatched in captivity to ~30 juvs, same as previous (USNMENT01377216); 1F, Fianarantsoa, 7 km W Ranomafana, 1100 m, 8–21 Oct 1988, montane rainforest, W.E. Steiner (USNMENT00879974); 1M,2F,2Juvs, Fianarantsoa, 7 km W Ranomafana, 1100 m, 1–7 Nov 1988, W.E. Steiner (USNMENT00879981); 1M,1Juv, Fianarantsoa, 7 km W Ranomafana, 21°12’S, 47°27’E, 1100 m, 1–9 Feb 1990, montane rainforest, W.E. Steiner (USNMENT00879978); 1F, Fianarantsoa, 7 km W Ranomafana, 900 m, 14–19 Mar 1990, W.E. Steiner (USNMENT00879963); 1F, Fianarantsoa, 7 km W Ranomafana, 21°12’S, 47°27’E, 1100 m, 1–7 Nov 1988, W.E. Steiner (USNMENT00879964); 1F, Fianarantsoa, 7 km W Ranomafana, 1100 m, 22–31 Oct 1988, W.E. Steiner (USNMENT00879979); 1F, Fianarantsoa, 7 km W Ranomafana, 21°12’S, 47°27’E, 900 m, 23–28 Feb 1990, W.E. Steiner (USNMENT00879966); 1F, Fianarantsoa, 7 km W Ranomafana, 900 m, 5 Mar 1990, W.E. Steiner (USNMENT00879989); 1F, Fianarantsoa, 7 km W Ranomafana, 900 m, 1–9 Feb 1990, montane rainforest, W.E. Steiner (USNMENT00879972); 1F, Fianarantsoa, 7 km W Ranomafana, 21°12’S, 47°27’E, 900 m, 1–7 Mar 1990, W.E. Steiner (USNMENT00879970); 1F, feeding on Oxyopidae spider, Fianarantsoa, 7 km W Ranomafana, 900 m, 3 Feb 1990, W.E. Steiner (USNMENT00879982).

##### Diagnosis.

Males and females are considered part of the “workmani group” based on having a single dorsal protuberance on the abdomen (a triangular shaped abdomen). Males of *E. workmani* are distinguished from all other species by the large bump on the pedipalpal bulbs (see arrow in Fig. 8H). Females are distinguished from *E. andriamanelo* by lacking the heavily sclerotized “T” shaped structure on the posterior of the bursa, from *E. andrianampoinimerina* by lacking the strong abdomen markings and by the “neck” having a tilt angle that is less than 80°, and from *E. ranavalona* by lacking the lime-green abdomen markings. Females are indistinguishable from the remaining “workmani group” species.

##### Description.

Male (based on CASENT9018947, from Antsiranana, Parc National de Marojejy, Madagascar). Total length 4.27, carapace 1.64 long, 1.36 wide. Abdomen 2.49 long, 1.92 wide, 2.67 high, with a prominent dorsal hump. Carapace tilt angle 75.56°, tilt height (CtH) 4.19, constriction 0.46, head length 1.57, neck length 2.38. CtH divided by carapace length 2.55. Cephalon with AME on a large bulge and 4 post-ocular protrusions on the apex of the cephalon (Fig. 8A), each provided with a short modified spine at the tip. Chelicerae 4.02 long, and with spine (Fig. 8A) 0.30 from base of chelicerae. Femur I 9.29 long. Sternum 1.11 long, 0.57 wide. Carapace, chelicerae, sternum and femora I & II reddish dark brown with many white setae (Fig. 8A). All coxae and legs III & IV yellowish brown. Legs III & IV with darker annulations on femora, tibiae and metatarsi. Abdomen yellowish brown, mottled with brown, and light brown book-lung covers, all covered with many white setae (Fig. 8A). Pedipalpal tegulum of the “workmani group” form), with apical conductor encircling a pit-like cavity (Fig. 8D–K). Conductor tip is a broad triangular point similar to other “workmani group” species except *E. andriamanelo* (Fig. 3H). MA with a bifurcation, variably shaped (Fig. 8C, L). Embolus similar to other “workmani group” species, being broad and complex with the sperm duct opening in the middle and sclerotization only at the tip (Fig. 8E, K–L).

Female (based on CASENT9018947, from Antsiranana, Parc National de Marojejy, Madagascar). Total length 4.53, carapace 1.62 long, 1.36 wide. Abdomen 2.88 long, 2.65 wide, 3.59 high, with dorsal hump. Carapace tilt angle 74.48°, tilt height (CtH) 3.90, constriction 0.46, head length 1.47, neck length 2.25. CtH divided by carapace length 2.41. Cephalon as in male. Chelicerae 3.77 long, and with spine 0.35 from base of chelicerae. Tarsus of pedipalps with ventral patch of long thick setae. Femur I 6.15 long. Sternum 1.04 long, 0.60 wide. Colors as in male, but generally darker. Female internal genitalia indistinguishable from other “workmani group” species (Fig. 8B).

##### Variation.

Total length 3.45–5.27 (males; n=5), 3.89–6.72 (females; n=5); Carapace length 1.37–2.17 (males; n=5), 1.45–2.40 (females; n=5); Femur I 5.09–6.10 times the length of carapace in males (n=5) and 3.75–4.04 times the length of carapace in females (n=5). CtH divided by carapace length 2.19–2.67 in males (n=5), 2.12–2.48 in females (n=5). Average femur I length 9.55 (males; n=5), 6.81 (females; n=5).

##### Natural history.

Specimens have been collected in montane and lowland rainforest through general collecting, beating vegetation, sweeping, raking, cryptic searching, and among fallen logs and litter at altitudes of 26–1200 m above sea level. Several specimens were collected with eggcases that look similar to eggcases from other “workmani group” species.

##### Distribution.

Widely distributed in the eastern rainforests of Madagascar from north to south (Fig. 29).

##### Nomenclatural remarks.

The holotype is a juvenile. We assume it to be the species we are describing because this species is the most widespread common species of the “workmani group”. Millot (1948) assumed the same thing.

### “Workmani group” spp. – unidentified specimens

There were many "workmani group" specimens that could not be confidently identified to a known species, listed below, due to the absence of adult male specimens and molecular data. Additionally, there was one penultimale male specimen that died while molting in captivity that was included in the molecular phylogeny of Wood et al. (2015). This specimen is likely a new species based on the phylogenetic relationships (shown as *Eriauchenius* sp. 1 in Fig. 1). However, since there are no known adult males, and only one adult female specimen, we chose not to describe this species at this time: 1 penultimate male, MADAGASCAR, Fianarantsoa, Parc National Andrigitra, 34 km S Ambalavao, 22°09'24.9"S, 46°57'08.7"E, 1830 m, 8–9 Jan 2009, primary montane rainforest, general collecting day, beating vegetation, H. Wood (USNMENTO01377166); 1F, Madagascar, Fianarantsoa, Parc National Andrigitra, 34 km S Ambalavao, 22°09'24.9"S, 46°57'08.7"E, 1830 m., 8–9 Jan 2009, primary montane rainforest, general collecting day, beating vegetation, H. Wood (USNMENT01377165).

**Additional unknown material examined.** MADAGASCAR: 1F, Antsiranana, Parc National de Marojejy, Manantenina River, 27.6 km 35° NE Andapa, 9.6 km 327° NNW Manantenina, 14°26'06"S, 49°45'36"E, 775m, 15–18 Nov 2003, rainforest, general collecting night, B.L. Fisher et al. (CASENT9018971); 1juv, Fianarantsoa, Forêt d’Analalava, 29.6 km 280° W Ranohira, 22°35'30"S, 45°7'42"E, 700 m, 1–5 Feb 2003, dry forest on sandy soil, general collecting, beating and puffing spiders, Fisher, Griswold et al. (CASENT9017402); 14juv, Toliara, Réserve Spéciale d’Ambohijanahary, Forêt d’Ankazotsihitafototra, 35.2 km 312° NW Ambaravaranala, 18°16'00"S, 45°24'24"E, 1050 m, 13-17 Jan 2003, montane rainforest, EB17 beating low vegetation, Fisher, Griswold et al. (CASENT9018107, CASENT9018226); 1F, Parc National Andohahela, Col du Sedro, 3.8 km 113° ESE Mahamavo, 37.6 km 341° NNW Tolagnaro, 24°45'50"S, 46°45'6"E, 900 m, 21–25 Jan 2002, montane rainforest, general collecting, beating and puffing spiders, B.L. Fisher et al. (CASENT9009553); 1juv, Toliara, Réserve Spéciale Kalambatritra, Betanana, 23°24'52"S, 46°27'32"E, 1360 m, 8 Feb 2009, montane rainforest, beating low vegetation, B.L. Fisher et al. (CASENT9034283); 1F, Antananarivo, Manjakatompo, 17 km W Ambatolampy, 18°58’S, 47°17’E, 1500 m, 11 Feb 2003, disturbed montane rainforest, EBD17 general collecting night, D. Silva, D. Andriamalala et al. (CASENT9005722); 3F,13juv,1 egg case, Antananarivo, Ambohimanga, 18°44’S, 47°34’E, 1400 m, 1 Nov 1993, J. Coddington, N. Scharff, S. Larcher, C. Griswold, and R. Andriamasimanana (CASENT 9010052, CASENT 9010065, CASENT 9048510); 1F,3juv, Antananarivo, Réserve Spéciale d’Ambohitantely, Forêt d’Ambohitantely, Jardin Botanique, 24.1 km 59° NE Ankazobe, 18°10'17"S, 47°16'55"E, 1620 m, 19 Mar 2003, montane rainforest, general collecting, D. Andriamalala, D. Silva, et al. (CASENT 9015898); 2F,1juv, Antananarivo, Réserve Spéciale d’Ambohitantely, Forêt d’Ambohitantely, 20 km NE Ankazobe, 18°12'29.6"S, 47°17'8.3"E, 1638m, 20 Mar 2003, forest fragment, montane rainforest, general collecting night, D. Andriamalala, D. Silva, et al. (CASENT9014893); 1juv, Toamasina, Mikira forest, 2.5 hour hike from Andaparaty, 29 km N Maroantsetra, 15°12'2.95"S, 49°36'55.0"E, 195m, 10–12 Dec 2008, primary montane rainforest, general collecting day, F. Alvarez-Padilla & H. Wood (USNMENT01377167); 1juv, Toliara, Parc National Andohahela, Parcelle I, Manangotry, off Rte. 118, 34 km N Taolagnaro, 24°44'35.0"S, 46°51'23.3"E, 670 m, 23 Dec 2008 – 3 Jan 2009, primary montane rainforest, general collecting day and night, F. Alvarez-Padilla & H. Wood (USNMENT01377168).

### The “bourgini group” species

#### The “enclosed embolus group”

##### Eriauchenius fisheri

Taxon classificationAnimaliaAraneaeArchaeidae

(Lotz, 2003) comb. n.

Figs 9 , 31

Afrarchaea fisheri Lotz, 2003: 234, fig. 6A–C.

###### Type material.

Female holotype: *Afrarchaea fisheri* Lotz, 2003, from Fianarantsoa, Reserve Andringitra, 8.5 km SE Antanitotsy, 22°10'S, 46°58'E, 1990 m, 6 Mar 1997, rainforest, sifted litter, B.L. Fisher (examined, deposited in CAS, CASENT9012340).

Other material examined: MADAGASCAR: 1M, Fianarantsoa, Reserve Andringitra, 38 km S Ambalavo, 22°12'S, 46°58'E, 1680 m, 23 Oct 1993, rainforest, sifted litter, B.L. Fisher (CASENT9018939); 1F, Fianarantsoa, Res. Special Ivohibe, 8.0 km E Ivohibe, 22°29.0'S, 46°58.1'E, 1200 m, 15–21 Oct 1997, forest, leaf litter, B.L. Fisher (CASENT9012344).

###### Diagnosis.

Males are distinguished from other “bourgini group” species except *E. harveyi* sp. n., *E. goodmani* sp. n., and *E. wunderlichi* sp. n., by having the pedipalpal tegulum of the “workmani group” form, with the apical conductor encircling a pit-like cavity (Fig. 9D–L). Males are distinguished from *E. goodmani* sp. n., *E. harveyi* sp. n. and *E. wunderlichi* sp. n. by having an s-shaped embolus (Fig. 9E, H, K–L). Females are distinguished from the “bourgini group” except for *E. goodmani* sp. n. and *E. wunderlichi* sp. n. by having a bursa with two large groups of poreplates on a sclerotized plate that covers the ventral side of the bursa (Fig. 9C). In contrast, in other “bourgini group” species the poreplates are in smaller clusters on the anterior side of the bursa. Females are distinguished from *E. goodmani* sp. n. by having a thinner posterior bar (Fig. 11C), and from *E. wunderlichi* sp. n. by lacking the large bulge in the center of the posterior bar (Fig. 12B). Females are indistinguishable from *E. harveyi* sp. n.

###### Description.

Female holotype (CASENT9012340, from Reserve Andringitra, Madagascar). This specimen was damaged during shipping so that the “head” is broken off from the “neck,” rendering some measurements impossible. Total length 2.38, carapace 1.11 long, 0.98 wide. Abdomen 1.16 long, 1.17 high. Carapace tilt height (CtH) 1.61, head length 0.98, carapace tilt angle, carapace constriction, and neck length unknown due to damage. CtH divided by carapace length 1.45. Cephalon with AME virtually flush with surrounding cuticle, and with a single pair of short modified spines at the apex (Fig. 9A). Chelicerae 1.86 long, and with a long spine 0.21 from base of chelicerae and projecting perpendicular to the cheliceral cuticle. Femur I 1.73 long. Sternum 0.72 long, 0.47 wide. Carapace, chelicerae, sternum and legs reddish brown with white setae, but reduced numbers compared to other *Eriauchenius*. All patella lighter in color, being more yellowish white. Abdomen mottled brown and beige, with tufts of white setae, although reduced in number compared to other *Eriauchenius* (Fig. 9A). Genitalia with a noncomplex FSGP, with posterior bar (not visible in Fig. 9B, but similar to Fig. 11B–C), and with “wings” reduced and nearly transparent, with poreplates in two large groups, divided down the middle and on a sclerotized plate that covers the ventral side of the bursa (Fig. 9C, similar to Fig. 11C).

Male paratype (CASENT9018939). Total length 2.18, carapace 1.01 long, 0.92 wide. Abdomen 1.13 long, 1.16 high. Carapace tilt angle 79.9°, tilt height (CtH) 1.53, constriction 0.65, head length 0.91, neck length 0.72 . CtH divided by carapace length 1.51. Cephalon as in female. Chelicerae 1.58 long, and with long spine 0.20 from base of chelicerae and projecting perpendicular. Femur I 1.64 long. Sternum 0.65 long, 0.44 wide. Colors as in female. Pedipalpal tegulum of the “workmani group” form, with apical conductor encircling a pit-like cavity (Fig. 9D–L). Conductor tip tapering off into a sharp point (Fig. 9F,I,L). MA trifurcating into three prongs (Fig. 9E, H, L), and with a sclerite (SC) that may be part of the conductor, thicker and more sclerotized than the transluscent thin structure seen in the “workmani group” (Fig. 9E, H). Embolus dark, wire-like and with two curves making an “s” shape (Fig. 9E, H, K–L).

###### Variation.

Total length 2.69–2.38 (females; n=2); Carapace length 1.11–1.17 (females; n=2); Femur I 1.48–1.55 times the length of carapace in females (n=2). CtH divided by carapace length 1.45–1.52 in females (n=2). Average femur I length 1.69 (females; n=2). For female CASENT9012344, carapace tilt angle 80.4, carapace constriction 0.83, and neck length 0.87.

###### Natural history.

Specimens were collected in rainforest in the leaf litter from 1200–1990 m in elevation.

###### Distribution.

Known only from around Andringitra Massif in southeast Madagascar (Fig. 31).

###### Nomenclatural remarks.

Distributions of *E. fisheri* and *E. harveyi* sp. n. are in close proximity, however, *E. fisheri* seems to occur at higher elevations than *E. harveyi* sp. n. Furthermore, *E. fisheri* is slightly larger than *E. harveyi* sp. n. Male and female conspecifics were associated based on these factors, but due to the small number of specimens available, this association may be incorrect. Future molecular work as well as additional collection of specimens from more localities can help resolve this issue.

##### Eriauchenius goodmani sp. n.

Taxon classificationAnimaliaAraneaeArchaeidae

http://zoobank.org/7D8FDDA9-F05D-42E9-BA6B-352AABDF70F2

Figs 10 , 31

###### Type material.

Male holotype: MADAGASCAR, Toliara, Réserve Naturelle Intégrale d’Andohahela, parcel 1, 15.0 km NW Eminiminy, camp 4, 24°34.2’S, 46°43.9’E, 1500 m, 17–27 Nov 1995, Steve Goodman (deposited in FM).

###### Other material examined.

Female paratype, same data as holotype, except 20.0 km SE Andranondambo, camp 5, 24°33.7’S, 46°43.3’E, 1875 m, 27 Nov – 5 Dec 1995 (FM).

###### Etymology.

The specific name is a patronym to honor Dr. Steven Goodman, who collected the specimens and for his extensive work on Madagascar’s biodiversity.

###### Diagnosis.

Males are distinguished from other “bourgini-group” species except *E. harveyi* sp. n., *E. fisheri*, and *E. wunderlichi* sp. n., by having the pedipalpal tegulum of the “workmani group” form (Fig. 10E), with the apical conductor encircling a pit-like cavity (Fig. 10D–E). Males are distinguished from *E. harveyi* sp. n., *E. fisheri*, and *E. wunderlichi* sp. n., by the conductor being greatly elongated and blunt at the tip (Fig. 10F). Females are distinguished from “bourgini group” species, except *E. harveyi* sp. n., *E. fisheri*, and *E. wunderlichi* sp. n., by having a bursa with two large groups of poreplates on a sclerotized plate that covers the ventral side of the bursa (Fig. 10C), whereas in other “bourgini group” species the poreplates are in smaller cluster on the anterior side of the bursa. Females are distinguished from *E. harveyi* sp. n. and *E. fisheri*, by having a thicker posterior bar on the internal genitalia (Fig. 10B), and distinguished from *E. wunderlichi* sp. n. females by lacking the large bulge in the center of the posterior bar (Fig. 12B).

###### Description.

Male holotype (FM, from Réserve Naturelle Intégrale d’Andohahela, Madagascar). Total length 2.62, carapace 1.01 long, 0.87 wide. Abdomen 1.34 long, 1.40 high. Carapace tilt angle 72.2°, tilt height (CtH) 1.62, constriction 0.64, head length 0.81, neck length 0.80. CtH divided by carapace length 1.60. Cephalon with AME virtually flush with surrounding cuticle, and with a single pair of short modified spines at the apex (Fig. 12A). Chelicerae 1.65 long, and with long spine 0.23 from base of chelicerae and projecting perpendicular to the cheliceral cuticle (Fig. 10A). Femur I 2.10 long. Sternum 0.73 long, 0.45 wide. Carapace, chelicerae, sternum and legs reddish brown with white setae. Abdomen mostly yellowish white, but mottled with light brown, interspersed with brown setae (Fig. 10A). Pedipalpal tegulum of the “workmani group” form, with apical conductor encircling a pit-like cavity (Fig. 10D–F). Conductor tip elongated with a blunt tip (Fig. 10F). MA not present, but SC present and heavily sclerotized and thicker than the transluscent thin structure seen in the “workmani group” (Fig. 10E). Embolus dark, thick, and notched at tip where the opening occurs (Fig. 10D).

Female paratype (FM, from Réserve Naturelle Intégrale d’Andohahela, Madagascar). The cephalon is damaged in this specimen making the head length measurement and cephalon spine count impossible. Total length 3.42, carapace 1.57 long, 1.38 wide. Abdomen 1.70 long, 1.88 high. Carapace tilt angle 65.6°, tilt height (CtH) 2.41, constriction 0.91, neck length 1.34. CtH divided by carapace length 1.54. Cephalon as in male. Chelicerae 2.87 long, and with long spine 0.20 from base of chelicerae and projecting perpendicular. Femur I 2.84 long. Sternum 1.00 long, 0.59 wide. Carapace, chelicerae, sternum and legs reddish brown with white setae. Abdomen darker than the male, mottled with dark brown areas and lighter yellowish areas, interspersed with brown setae. Genitalia with a noncomplex FSGP, with posterior bar, “wings” present but translucent, with poreplates in two large groups, divided down the middle and on a sclerotized plate that covers the ventral side of the bursa (Fig. 10B–C).

###### Variation.

no other known material.

###### Natural history.

Specimens were collected in rainforest from 1500–1875 m in elevation.

###### Distribution.

Known only from Réserve Naturelle Intégrale d’Andohahela in southeast Madagascar (Fig. 31).

##### Eriauchenius harveyi sp. n.

Taxon classificationAnimaliaAraneaeArchaeidae

http://zoobank.org/DE41E9A4-A2F5-46CF-AB18-38849BEC93FD

Figs 11 , 32

###### Type material.

Male holotype: Madagascar, Fianarantsoa, Massif Andringitra, 43 km S Ambalavo, 22°14'S, 47°00'E, 825 m, 4 Oct 1993, rainforest, sifted litter B.L. Fisher (deposited in CAS; CASENT9012110)

###### Other material examined.

MADAGASCAR: Female paratype, same data as holotype, (CASENT9012342); 1M, Fianarantsoa, 9.0 km NE Ivohibe, 22°25.6'S, 46°56.3'E, 900 m, 12–17 Nov 1997, forest, leaf litter, B.L. Fisher (CASENT9012109)

###### Etymology.

The specific name is a patronym to honor Dr. Mark Harvey for his work on the Australian archaeids.

###### Diagnosis.

Males are distinguished from other “bourgini group” species except *E. fisheri*, *E. goodmani* sp. n. and *E. wunderlichi* sp. n. by having the pedipalpal tegulum of the “workmani group” form, with the apical conductor encircling a pit-like cavity (Fig. 11D–K). Males are distinguished from *E. fisheri* by having a comparably straighter embolus, with only a broad curve (Fig. 11E, H, J–K), rather than s-shaped, from *E. goodmani* sp. n. by lacking the elongated conductor that is broad and blunt at the tip (Fig. 10F), rather than tapering off to a fine point (Fig. 11F, I), and from *E. wunderlichi* sp. n. by lacking an elongation in the apical portion of the tegulum where the conductor swirls around making the bulb almost twice as long as it is wide (Fig. 12F). Females are distinguished from the “bourgini group” except for *E. goodmani* sp. n. and *E. wunderlichi* sp. n. by having two large groups of poreplates on a sclerotized plate that covers the ventral side of the bursa (Fig. 11C), whereas in other “bourgini group” species the poreplates are in smaller clusters on the anterior side of the bursa. Females are distinguished from *E. goodmani* sp. n. by having a thinner posterior bar (Fig. 11B), and from *E. wunderlichi* sp. n. by lacking the large bulge in the center of the posterior bar (Fig. 12B). Females are indistinguishable from *E. fisheri*.

###### Description.

Male holotype (CASENT9012110, from Massif Andringitra, Madagascar). Total length 2.05, carapace 0.87 long, 0.75 wide. Abdomen 1.08 long, 1.15 high. Carapace tilt angle 76.1°, tilt height (CtH) 1.29, constriction 0.55, head length 0.73, neck length 0.61. CtH divided by carapace length 1.48. Cephalon with AME virtually flush with surrounding cuticle, and with 2 short modified spines at the apex (Fig. 11A). Chelicerae 1.30 long, and with a long spine 0.15 from base of chelicerae and projecting perpendicular to the cheliceral cuticle (Fig. 11L, arrow). Femur I 1.36 long. Sternum 0.56 long, 0.37 wide. Carapace, chelicerae, sternum and legs reddish brown with white setae, but reduced numbers compared to other *Eriauchenius*. All patella lighter in color, being more yellowish white. Abdomen mottled brown and beige, with tufts of white setae, although reduced in number compared to other *Eriauchenius* (Fig. 11A). Pedipalpal (Fig. 11D–K) tegulum of the “workmani group” form (Fig. 11D–E), with apical conductor encircling a pit-like cavity. Conductor tip tapering off into a sharp point (Fig. 11I). MA broad, thick and triangular (Fig. 11H, K), and sclerite SC heavily sclerotized and thicker than the transluscent thin structure seen in the “workmani group”. Embolus dark, broadly curved, and wire-like (Fig. 11G–K).

Female paratype (CASENT9012342). Total length 2.31, carapace 0.97 long, 0.86 wide. Abdomen 1.23 long, 1.24 high. Carapace tilt angle 76.3°, tilt height (CtH) 1.45, constriction 0.65, head length 0.82, neck length 0.70. CtH divided by carapace length 1.49. Cephalon as in male. Chelicerae 1.44 long, and with long spine 0.17 from base of chelicerae and projecting perpendicular. Femur I 1.50 long. Sternum 0.63 long, 0.42 wide. Colours as in male. Genitalia with a noncomplex sclerotized plate, with posterior bar, and with “wings” reduced, with poreplates in two large groups, divided down the middle of the bursa and on a sclerotized plate that covers the ventral side of the bursa (Fig. 11B–C).

###### Variation.

Total length 2.05–2.12 (males; n=2); Carapace length 0.87–0.92 (males; n=2); Femur I 1.57–1.64 times the length of carapace in males (n=2). CtH divided by carapace length 1.47–1.48 in males (n=2). Average femur I length 1.43 (males; n=2).

###### Natural history.

Specimens were collected in rainforest in the leaf litter from 825–900 m in elevation.

###### Distribution.

Known only from around Andringitra Massif in southeast Madagascar (Fig. 32).

##### Eriauchenius wunderlichi sp. n.

Taxon classificationAnimaliaAraneaeArchaeidae

http://zoobank.org/0CAFB94F-3D86-4E11-825D-76E83E5D22CC

Figs 12 , 31

###### Type material.

Male holotype: MADAGASCAR, Fianarantsoa, Parc National Befotaka-Midongy, Papango, 28.5 km S Midongy-Sud, Mount Papango, 23°50'27"S, 46°57'27"E, 1250 m, 17–19 Nov 2006, montane rainforest, sifted litter, B.L. Fisher et al. (deposited in CAS; CASENT9062702).

###### Other material examined.

Female paratype, MADAGASCAR: Toliara, Réserve Spéciale Kalambatritra, Ampanihy, 23°27'49"S, 46°27'47"E, 1270 m, 9–10 Feb 2009, montane rainforest, sifted litter, B.L. Fisher et al. (CASENT9062648).

###### Etymology.

The specific name is a patronym to honor Jörg Wunderlich, for his work documenting fossil archaeids.

###### Diagnosis.

Males are distinguished from other “bourgini group” species except *E. harveyi* sp. n., *E. fisheri*, and *E. goodmani* sp. n., by having the pedipalpal tegulum of the “workmani group” form, with the apical conductor encircling a pit-like cavity (Fig. 12D–F). Males are distinguished from *E. harveyi* sp. n., *E. fisheri*, and *E. goodmani* sp. n., by having an elongated apical portion of the tegulum, so that the bulb is almost twice as long as it is wide (Fig. 12F). Females are distinguished from the “bourgini group,” except from *E. harveyi* sp. n., *E. fisheri*, and *E. goodmani* sp. n., by having a bursa with two large groups of poreplates on a sclerotized plate that covers the ventral side of the bursa (Fig. 12B–C), whereas in other “bourgini group” species the poreplates are in smaller clusters on the anterior side of the bursa. Females are further distinguished from the “bourgini group” by having a large bulge on the center of the posterior bar (Fig. 12B, arrow).

###### Description.

Male holotype (CASENT9062702, from Parc National Befotaka-Midongy, Madagascar). The abdomen of this specimen is missing making some measurements impossible. Carapace 1.11 long, 0.89 wide. Carapace tilt angle 76.7°, tilt height (CtH) 1.80, constriction 0.76, head length 1.02, neck length 0.87. CtH divided by carapace length 1.62. Cephalon with AME virtually flush with surrounding cuticle, and with 2 short modified spines at the apex. Chelicerae 1.78 long, and with long spine 0.19 from base of chelicerae and projecting perpendicular to the cheliceral cuticle (Fig. 12A). Femur I 1.98 long. Sternum 0.71 long, 0.42 wide. Carapace, chelicerae, sternum and legs reddish brown with white setae. Legs with darker annulations on tibiae and metatarsi. Abdomen color unknown. Pedipalpal tegulum of the “workmani group” form, with apical conductor encircling a pit-like cavity, however the apical portion of the tegulum is elongated so that the bulb is almost twice as long and wide (Fig. 12D–F). Conductor tip tapering off into a sharp point (Fig. 12F). MA rounded and small, and nearly hidden behind sclerite SC and embolus. Sclerite SC long and heavily sclerotized and in close proximity and running parallel to the embolus. Embolus dark, thick, and with a swirl at the base and blunt at the tip (Fig. 12E–D).

Female paratype (CASENT9062648). Total length 2.59, carapace 1.25 long, 1.04 wide. Abdomen 1.26 long, 1.36 high. Carapace tilt angle 73.5°, tilt height (CtH) 1.99, constriction 0.78, head length 1.02, neck length 1.02. CtH divided by carapace length 1.59. Cephalon as in male. Chelicerae 2.18 long, and with long spine 0.24 from base of chelicerae and projecting perpendicular (Fig. 12A). Femur I 2.02 long. Sternum 0.82 long, 0.51 wide. Carapace, chelicerae, sternum and legs orangish brown with brown setae. Abdomen mottled with dark brown areas and lighter yellowish areas, interspersed with brown setae (Fig. 12A). Genitalia with a noncomplex sclerotized plate with “wings” translucent at the tips. Posterior bar with a large bulge in the center (Fig. 12B, arrow). Bursa with poreplates in two large groups, divided down the middle and positioned on a sclerotized plate that covers the ventral side of the bursa (Fig. 12C).

###### Variation.

no other known material.

###### Natural history.

Specimens were collected in montane rainforest from 1250–1270 m in elevation by sifting litter.

###### Distribution.

Known only from southeast Madagascar (Fig. 31).

###### Nomenclatural remarks.

The male and female of *E. wunderlichi* sp. n. occur in rainforest areas in southwestern Madagascar that are not contiguous. The male and female may not be conspecifics, however based on morphology they likely belong in the “enclosed embolus group.” Future molecular work as well as additional collection of specimens from more localities can help resolve these issues.

#### The “exposed embolus group”

##### Eriauchenius bourgini

Taxon classificationAnimaliaAraneaeArchaeidae

(Millot, 1948)

Figs 13 , 31

Archaea bourgini Millot, 1948: 10, figs 1C,2C,3B,3E.
Archaea bourgini Legendre, 1970: 26, figs 10–11,14C.

###### Type material.

As *Archaea bourgini* Millot, 1948: 6M, 3F, Madagascar, La Mandraka (examined, deposited in MNHN; MNHN 13/1970).

Material described and other material examined: 3M,5F,4Juvs, MADAGASCAR: Antananarivo, Réserve Spéciale d’Ambohitantely, Forêt d’Ambohitantely, 20.9 km 72° NE Ankazobe, 18°13'31"S, 47°17'13"E, 1410 m, 17–22 Apr 2001, montane rainforest, EB17 beating low vegetation, Fisher, Griswold et al. (CASENT9001207)

###### Diagnosis.

Males and females are distinguished from other *Eriauchenius* by having pointed extensions on coxae I (Fig. 13L, arrows). Males and females of *E. bourgini* and *E. zirafy* sp. n. are also distinguished by having two large protrusions on the crown of the cephalon (Fig. 13A). Females of *E. bourgini* and *E. zirafy* sp. n. are indistinguishable but are different from all other *Eriauchenius* by having two sclerotized invaginations on the bursa (Figs 13B, 22B, arrows). In *E. bourgini* males the conductor has four elaborate processes (Fig. 13D–K), and in and *E. zirafy* sp. n. males, five elaborate processes (Fig. 22D–K).

###### Description.

Male (based on CASENT9001207, from Réserve Spéciale d’Ambohitantely, Madagascar). Total length 1.94, carapace 0.79 long, 0.74 wide. Abdomen 1.09 long, 0.97 high. Carapace tilt angle 72.1°, tilt height (CtH) 1.80, constriction 0.33, head length 0.89, neck length 0.93. CtH divided by carapace length 2.28. Cephalon with AME on a large bulge and 4 post-ocular spines on the crown of the cephalon, with the posterior pair on large protrusions and the anterior pair not on protrusions, and with 1 small spine between the LE and median eyes (on each side, for a total of 2). Chelicerae 1.68 long, and with a short spine 0.70 from base of chelicerae, projecting downward. Femur I 2.13 long. Sternum 0.51 long, 0.36 wide. Carapace, chelicerae, and sternum dark reddish brown with white setae. Coxae and legs lighter brown, with darker annulations on tibiae and metatarsi. Coxae I with posterior extensions (Fig. 13L). Abdomen mottled brown and beige, with a bright white patch on each lateral side, with tufts of white setae (as in Fig. 22A from *E. zirafy* sp. n.). Pedipalpal bulb with a small membraneous sac on the retrolateral side, with the base of the conductor small and triangular (labeled “c” in Fig. 13D, F–G), and with the remainder of the conductor wrapping around the embolus and with four long processes (Fig. 13D–F). The embolus is thick and contains several processes.

Female (based on CASENT9001207, from Réserve Spéciale d’Ambohitantely, Madagascar). Total length 1.89, carapace 0.74 long, 0.72 wide. Abdomen 1.09 long, 1.98 high. Carapace tilt angle 63.0°, tilt height (CtH) 1.85, constriction 0.36, head length 0.92, neck length 0.97. CtH divided by carapace length 2.50. Cephalon as in male. Chelicerae 1.73 long, and with small spine 0.84 from base of chelicerae. Femur I 2.03 long. Sternum 0.49 long, 0.33 wide. Colors as in male, except abdomen lacking the bright white lateral patches (Fig. 13A). Female genitalia with a small and simple FSGP, and posterior bar highly reduced (Fig. 13B). Bursa lacking poreplates and instead having a sclerotized invagination on each lateral side of the anterior side of the bursa (Fig. 13C, arrows).

###### Variation.

Total length 1.76–1.94 (males; n=5), 1.89–2.15 (females; n=5); Carapace length 0.73–0.79 (males; n=5), 0.74–0.81 (females; n=5); Femur I 2.54–2.71 times the length of carapace in males (n=5) and 2.45–2.75 times the length of carapace in females (n=5). CtH divided by carapace length 2.20–2.39 in males (n=5), 2.37–2.54 in females (n=5). Average femur I length 2.09 (males; n=5), 2.05 (females; n=5).

###### Natural history.

Specimens were collected at 1410 m in elevation in montane rainforest by beating vegetation.

###### Distribution.

Known only from central-eastern Madagascar (Fig. 31).

##### Eriauchenius lukemacaulayi sp. n.

Taxon classificationAnimaliaAraneaeArchaeidae

http://zoobank.org/86EE57CA-4733-4A3C-9D95-118DADDEE746

Figs 14 , 32

###### Type material.

Male holotype: Madagascar, Fianarantsoa, Parc National Andrigitra, 34 km S Ambalavao, 22°09'24.9"S, 46°57'08.7"E, 1830 m, 8–9 Jan 2009, primary montane rainforest, general collecting day, beating vegetation, H. Wood (deposited in USNM: USNMENT01377191).

###### Other material examined.

Female paratype, same as holotype except general collecting day (USNMENT01377192); 1F, 1 eggcase, same as holotype except general collecting day, 1.5 feet above ground in a log leaning against tree (USNMENT01377193); 2F, same as holotype except hand collected in litter ~6 feet off ground in the leaf bases of *Pandanus* (USNMENT01377194, USNMENT01377185); 1F, Fianarantsoa, Parc National Andrigitra, 34 km S Ambalavao, 22°08'48.9"S, 46°57'03.4"E, 1580 m, 7 Jan 2009, primary montane rainforest, hand collected under dead log, H.Wood (USNMENT01377186).

###### Etymology.

The specific name is a patronym to honor Dr. Luke Macaulay for his help collecting palpimanoid spiders.

###### Diagnosis.

Male is distinguished from other “bourgini group” species except *E. milloti* sp. n. and *E. pauliani* (and presumably *E. milajaneae* sp. n.) by the presence of a lateral process on the conductor (Fig. 14G–I, asterisk), which is narrow in *E. lukemacaulayi* and *E. milloti* (Fig. 14H). Males are distinguished from *E. milloti* sp. n. by lacking the quarter-moon-like-shape of the palpal bulb in *E. milloti* (Fig. 17D–L). *Eriauchenius lukemacaulayi* sp. n., *E. milajaneae* sp. n., and *E. milloti* sp. n. (and presumably *E. pauliani*) females are distinguished from other “bourgini group” species by the enlargement of the posterior bar. While this bar is present in other “bourgini group” species, in *E. lukemacaulayi* sp. n., *E. milajaneae* sp. n., and *E. milloti* sp. n. this bar is large and curved into a u-shape. In *E. lukemacaulayi* sp. n., either end of this bar extends anterior just past the bottom of the “wings” (Fig. 14B), whereas in *E. milajaneae* sp. n. and *E. milloti* sp. n. the curved bar goes at least to the middle of the “wings” (Figs 16B, 17B). *Eriauchenius lukemacaulayi* sp. n. females are also distinguished by the rounded posterior elongation of the FSGP that curves to the dorsal (Fig. 14F, arrow).

###### Description.

Male holotype (USNMENT01377191, from Parc National Andrigitra, Madagascar). Total length 2.38, carapace 1.05 long, 0.97 wide. Abdomen 1.30 long, 1.47 high. Carapace tilt angle 63.59°, tilt height (CtH) 2.29, constriction 0.55, head length 1.03, neck length 1.04. CtH divided by carapace length 2.18. Cephalon with AME on a large bulge and 4 post-ocular spines on the crown of the cephalon, not on protrusions, and with 1 small spine between the LE and median eyes (on each side, for a total of 2). Chelicerae 1.99 long, and with a spine 0.27 from base of chelicerae and projecting perpendicular to the cheliceral cuticle. Femur I 2.56 long. Sternum 0.66 long, 0.44 wide. Carapace, chelicerae, sternum, and legs reddish brown with white setae. All legs with parts that are lighter brown and parts with darker annulations. Abdomen dark brown mottled with yellowish white spots and interspersed with white setae. Pedipalpal bulb with a membranous sac above the base of the embolus on the retrolateral side, with a greatly exposed embolus that is encircled by the conductor as conductor tapers off (Fig. 14D–I). Conductor with a constriction, followed by a narrow, curved process (Fig. 14G–I, asterisk) and a bulge (Fig. 14H, arrow). Embolus broad and dark (Fig. 14G–I).

Female paratype (USNMENT01377192). Total length 2.42, carapace 1.00 long, 0.93 wide. Abdomen 1.34 long, 1.56 high. Carapace tilt angle 65.6°, tilt height (CtH) 2.18, constriction 0.53, head length 0.97, neck length 1.04. CtH divided by carapace length 2.18. Cephalon as in male. Chelicerae 2.04 long, and with short spine 0.37 from base of chelicerae and projecting downward (Fig. 14A). Femur I 2.40 long. Sternum 0.63 long, 0.44 wide. Colours as in male, expect anterior of abdomen dark brown with yellowish spots and posterior of abdomen mostly yellowish white with some white patches (Fig. 14A). Female genitalia FSGP with a posterior elongation that curves dorsally (Fig. 14F, arrow), and with lateral bulges at the base of each “wing” (Fig. 14B), with a large U-shaped posterior bar that extends just past the posterior portion of the “wings” (Fig. 14B), with poreplates in one group on each lateral side of the bursa anterior (Fig. 14C).

###### Variation.

Total length 2.17–3.09 (females; n=5); Carapace length 1.01–1.06 (females; n=5); Femur I 2.37–2.55 times the length of carapace in females (n=5). CtH divided by carapace length 2.09–2.25 in females (n=5). Average femur I length 2.48 (females; n= 5).

###### Natural history.

Specimens were collected in leaf litter, in and under logs and through beating vegetation in primary rainforest from 1580–1830 m in elevation.

###### Distribution.

Known only from Parc National Andrigitra in southeast Madagascar (Fig. 32).

##### Eriauchenius mahariraensis

Taxon classificationAnimaliaAraneaeArchaeidae

(Lotz, 2003) comb. n.

http://zoobank.org/9C1C1CD8-B13D-41EF-ABCC-8AD9468E8B4F

Figs 15 , 32

Afrarchaea mahariraensis Lotz, 2003: 237, figs 5A, 8A–B.

###### Type material.

Female holotype: as *Afrachaea mahariraensis* Lotz, 2003, from MADAGASCAR, Ranomafana N.P., Maharira, trail, 10 Apr 1992, sifting, Emile for Kariko/Roth (examined, deposited in MCZ).

###### Other material examined.

MADAGASCAR: male paratype, Fianarantsoa, Parc National Ranomafana, Vohiparara, 3.6 km W Ranomafana, 21°14.243’S, 47°23.842’E, 1150 m, 13–14 Jan 2009, primary montane rainforest, sifting dead ferns on ground, C. Griswold, A. Saucedo and H. Wood (USNMENT01377173)

###### Diagnosis.

Males and females are distinguished from other *Eriauchenius*, except *E. ratsirarsoni* and *E. sama* sp. n., by the presence of 4 small spines on the apex of the cephalon, and having the cheliceral spine pointing perpendicular (as in Fig. 11L). In the holotype female the cheliceral spine is broken off, but the socket shape suggests the spine would be at a perpendicular orientation as it is in the paratype male. *Eriauchenius mahariraensis* is also distinguished from other *Eriauchenius* males, by having membranous tissue close to the conductor tip (Fig. 15E–L), and other *Eriauchenius* females by having a very reduced FSGP with the width divided by height greater than 2 (Fig. 15B).

###### Description.

Female holotype (MCZ, from Parc National Ranomafana, Madagascar). Total length 1.91, carapace 0.74 long, 0.69 wide. Abdomen 1.06 long, 1.24 high. Carapace tilt angle 64.4°, tilt height (CtH) 1.32, constriction 0.44, head length 0.68, neck length 0.64. CtH divided by carapace length 1.77. Cephalon with AME on a small bulge. Cephalon with 4 small post-ocular spines (one is missing or broken off) on the crown of the cephalon, not on protrusions, and 1 spine between the LE and AME (on each lateral side, for a total of 2, but broken off on the right side). Chelicerae 1.33 long, and with a spine 0.24 from base of chelicerae, however, the spines are broken off and only the socket remains; the socket shape suggests the spines would projecting perpendicular to the cheliceral cuticle. Femur I 1.46 long. Sternum 0.51 long, 0.35 wide. Carapace, chelicerae, sternum, and legs reddish brown with white setae; patellas lighter brown. Abdomen anterior dark brown with light circular patches and posterior light tan; abdomen with white and brown setae (Fig. 15A). Female genitalia with a small and simple FSGP, with “wings” highly reduced; posterior bar is present, but the dissection was such that it remains attached to the abdomen and is not visible in Fig. 15B–C; with poreplates in one small group on each lateral side of the bursa anterior, and a small sclerotized piece on the ventral side of the poreplates (Fig. 15C, arrow).

Male paratype (USNMENT01377173). Total length 1.64, carapace 0.78 long, 0.68 wide. Abdomen 0.83 long, 0.97 high. Carapace tilt angle 66.7°, tilt height (CtH) 1.31, constriction 0.44, head length 0.72, neck length 0.61. CtH divided by carapace length 1.69. Cephalon with AME on a small bulge, and with 4 small post-ocular spines (one is missing or broken off) on the crown of the cephalon, and 1 spine between the LE and AME (on each side, for a total of 2). Chelicerae 1.23 long, and with small spine 0.17 from base of chelicerae (Fig. 15A). Femur I 1.63 long. Sternum 0.49 long, 0.32 wide. Cephalothorax colors as in female. Abdomen mostly light tan, but mottled with dark brown areas that have light circular patches; with white and brown setae; posterior of abdomen with bright white areas. Pedipalpal bulb with a small membraneous sac above the embolus base, with a greatly exposed broad and dark embolus that is encircled by the conductor (Fig. 15D–L). Conductor with a membranous area at the distal end that swirls around the embolus (Fig. 15D–L).

###### Variation.

no other known material.

###### Natural history.

Male specimen was collected at 1150 m in elevation. Both specimens were collected in montane rainforest by general sifting, or sifting dead ferns on the ground.

###### Distribution.

Known only from Parc National Ranomafana in central-eastern Madagascar (Fig. 32).

###### Nomenclatural remarks.

*E. mahariraensis* and *E. sama* sp. n. both occur at Parc National Ranomafana. Males and females were associated based on body size and carapace shape.

##### Eriauchenius milajaneae sp. n.

Taxon classificationAnimaliaAraneaeArchaeidae

http://zoobank.org/FC344590-1068-4C17-8B7A-AE6919AEB5D7

Figs 16 , 31

###### Type material.

Female holotype: MADAGASCAR, Toliara, Parc National Andohahela, Col du Sedro, 3.8 km 113° ESE Mahamavo, 37.6 km 341° NNW Tolagnaro, 24°45'50"S, 46°45'6"E, 900 m, 21–25 Jan 2002, montane rainforest, EB17 beating low vegetation, B.L. Fisher et al. (CASENT 9009881).

###### Other material examined.

MADAGASCAR: 1F,1Juv, same locality and data as holotype, general collecting at night (CASENT9009481).

###### Etymology.

The specific name is a patronym for Mila Jane Macaulay in the hope that one day she will go to Andohahela to find this spider.

###### Diagnosis.

Females of *E. milajaneae* sp. n., *E. milloti* sp. n., and *E. lukemacaulayi* sp. n. (and presumably *E. pauliani*, although the females are unknown) are distinguished from other “bourgini group” species by the enlargement of the U-shaped posterior bar of the internal genitalia. While the PB is present in other “bourgini group” species, in *E. milajaneae* sp. n., *E. milloti* sp. n., and *E. lukemacaulayi* sp. n., this bar is very large and curved into a u-shape (Figs 14B, 16B, 17B). In *E. milajaneae* sp. n. and *E. milloti* sp. n., each curved end of the PB extends at least to the middle of the “wings”. *Eriauchenius milajaneae* sp. n. is also distinguished by the rectangular shape of the posterior of the FSGP (Fig. 16B, arrow), and by the long neck that does not have a strong constriction, so that the “head” and “neck” are not clearly separated (Fig. 16A).

###### Description.

Female holotype (CASENT 9009881, Parc National Andohahela, Madagascar). Total length 2.33, carapace 0.94 long, 0.81 wide. Abdomen 1.35 long, 1.48 high. Carapace tilt angle 68.73°, tilt height 2.32, constriction 0.43, head length 0.97, neck length 1.23. CtH divided by carapace length 2.47. Cephalon with AME on a large bulge, with 4 small post-ocular spines at the crown of the cephalon, not on protrusions. Chelicerae 2.03 long, and with short spine 0.28 from base of chelicerae that projects downward (Fig. 16A). Femur I 2.84 long. Sternum 0.60 long, 0.38 wide. Carapace, chelicerae, and sternum reddish brown with white setae. All legs light brown, with darker annulations. Abdomen beige, mottled with brown and with tufts of white setae (Fig. 16A). Female genitalia FSGP with a broad, rectangular posterior elongation (Fig. 16B, arrow), PB large and U-shaped and extends about halfway past the “wings” (Fig. 16B). Poreplates in one group on each lateral side of the bursa anterior (Fig. 16C).

Male: unknown.

###### Variation.

Total length 2.33–2.38 (females; n=2); Carapace length 0.94–0.95 (females; n=2); Femur I 2.99–3.02 times the length of carapace in females (n=2). CtH divided by carapace length 2.44–2.47 in females (n=2). Average femur I length 2.84 (females; n=2).

###### Natural history.

The two known adult specimens were caught in montane rainforest through beating vegetation and general collection at night from 900 m in elevation.

###### Distribution.

Known only from the type locality in Parc National Andohahela in southeast Madagascar (Fig. 31).

##### Eriauchenius milloti sp. n.

Taxon classificationAnimaliaAraneaeArchaeidae

http://zoobank.org/FE5598D8-5BDD-4F04-949F-9AA88BF01F4D

Figs 17 , 32

###### Type material.

Male holotype: MADAGASCAR, Toliara, Parc National Andohahela, Parcelle I, Manangotry, off Rte. 118, 34 km N Taolagnaro, 24°44'35.0"S, 46°51'23.3"E, 670 m, 23 Dec 2008 – 3 Jan 2009, primary montane rainforest, general collecting day and night, turning over dead palm frond bases on the ground, F. Alvarez-Padilla & H. Wood (deposited in USNM: USNMENT01377187).

###### Other material examined.

Female paratype, 1 eggcase, same data as holotype (USNMENT01377188); 3M,11F,1 eggcase, same as previous (USNMENT01377189, USNMENT01377180, USNMENT01377181, USNMENT01377182, USNMENT01377183, USNMENT01377179, USNMENT01377171, USNMENT01377172); 2M3F, same as previous except turning over dead palm frond bases on the ground, F. Alvarez-Padilla & H. Wood (USNMENT01377176, USNMENT01377177); 1Juv, same as previous except general collecting day and night, beating vegetation (USNMENT01377178); 1M, Toliara, Foret Classee Tsikongambarika I, Ivolo forest, Andily, ca. Fort Dauphin, 24°56'13.5"S, 46°55'58.4"E, 20 m, 13 Mar 2003, rainforest, Ludd tree trunks/litter, D. Andriamalala, D. Silva et al. (CASENT9015862); 1Juv, Toliara, Foret Classee Tsitongambarika, Cascade trail hike, 7.5 km NW Taolagnaro, 24°59'11.9"S, 46°55'34.7"E, 100 m, 24 Dec 2008, primary montane rainforest, general collecting day and night, F. Alvarez-Padilla & H. Wood (USNMENT01377184); 1Juv, Toliara, Parc National Andohahela, Parcelle I, Manangotry, off Rte. 118, 32 km N Taolagnaro, 24°45'50.1"S, 46°51'23.5"E, 100 m, 25 Dec 2008, rainforest near stream surrounded by disturbed slash/burn forest, sifting and mini-winkler extraction of leaf litter, F. Alvarez-Padilla & H. Wood (USNMENT01377175).

###### Etymology.

The specific name is a patronym to honor Dr. Jacques Millot for his work describing Madagascan archaeids.

###### Diagnosis.

Males distinguished from other “bourgini group” species except *E. pauliani* and *E. lukemacaulayi* sp. n. (and presumably *E. milajaneae* sp. n., although the males are unknown) by the presence of a lateral process on the conductor, which is narrow in *E. lukemacaulayi* sp. n. and *E. milloti* (Figs 14E, G–I, 17D–I, asterisk), but is broader in *E. pauliani* (Fig. 18C–D, F–H, asterisk). *E. milloti* sp. n. is distinguished from *E. lukemacaulayi* sp. n. by the quarter-moon-shape of the pedipalpal bulbs (Fig. 17D–F, J–L). Females of *E. lukemacaulayi* sp. n., *E. milajaneae* sp. n., and *E. milloti* sp. n. (and presumably *E. pauliani*) are distinguished from other “bourgini group” females by the enlarged, extended U-shaped PB (Figs 14B, 16B, 17B). The PB is smaller and thiner in other “bourgini group” females. In *E. milloti* sp. n. the curved ends of the PB extend all the way to the anterior edge of the FSGP (Fig. 17B), whereas in *E. milajaneae* sp. n. and *E. lukemacaulayi* sp. n. the curved ends do not go past the anterior of the “wings” (Figs 14B, 16B).

###### Description.

Male holotype (USNMENT01377187, from Parc National Andohahela, Madagascar). Total length 2.18, carapace 0.91 long, 0.85 wide. Abdomen 1.23 long, 1.31 high. Carapace tilt angle 69.58°, tilt height (CtH) 1.89, constriction 0.47, head length 0.81, neck length 0.92. CtH divided by carapace length 2.08. Cephalon with AME on large bulge. Cephalon with 4 short post-ocular spines at the crown, not on protrusions. Chelicerae 1.81 long, and with short spine 0.30 from base of chelicerae and projecting downward (Fig. 17A). Femur I 2.53 long. Sternum 0.59 long, 0.38 wide. Carapace, chelicerae, and sternum reddish brown with white setae, although chelicerae lighter brown in middle. All legs with parts that are lighter brown and parts with darker annulations. Abdomen yellowish white, with scattered small and large dark brown areas with yellowish spots, and with tufts of white setae (Fig. 17A). Pedipalpal bulb with a membranous sac above the base of the embolus, with a greatly exposed broad and dark embolus that is encircled by the conductor (Fig. 17D–L). Conductor with a small narrow process (Fig. 17D–I, asterisk) followed by a constriction (Fig. 17H, arrow) that is then followed by a large bulge on the retrolateral side.

Female paratype (USNMENT01377188). Total length 2.33, carapace 0.97 long, 0.88 wide. Abdomen 1.33 long, 1.51 high. Carapace tilt angle 62.94°, tilt height (CtH) 1.99, constriction 0.48, head length 0.87, neck length 0.95. CtH divided by carapace length 2.05. Cephalon as in male. Chelicerae 1.89 long, and with short spine 0.33 from base of chelicerae and projecting downward. Femur I 2.48 long. Sternum 0.64 long, 0.41 wide. Colors as in male, although abdomen with a greater degree of dark brown. Female genitalia FSGP with a rounded posterior elongation (Fig. 17B); PB large and U-shaped, extending to the anterior edge of the FSGP (Fig. 17B); poreplates in one group on each lateral side of the bursa anterior (Fig. 17C).

###### Variation.

Total length 1.78–1.89 (males; n=5), 1.94–1.99 (females; n=5); Carapace length 0.88–0.91 (males; n=5), 0.94–0.99 (females; n=5); Femur I 2.73–2.82 times the length of carapace in males (n=5), 2.55–2.64 in females (n=5); CtH divided by carapace length 1.98–2.08 in males (n=5), 1.99–2.08 in females (n=5). Average femur I length 2.49 in both males (n=5) and females (n= 5).

###### Natural history.

In primary rainforest from 20–640 m in elevation. Collected day and night, turning over dead palm frond bases on the ground, in litter, and on tree trunks.

###### Distribution.

Known only from Parc National Andohahela and surroundings in the southeastern part of Madagascar (Fig. 32).

##### Eriauchenius pauliani

Taxon classificationAnimaliaAraneaeArchaeidae

(Legendre, 1970)

Figs 18 , 32

Archaea pauliani Legendre, 1970: 21, figs 6–7, plate 3, fig. 4.

###### Type material.

Male holotype: as *Archaea pauliani* Legendre, 1970: Madagascar, Andohahelo (Fort Dauphin), 1800 m., Jan /1954, R. Paulian (examined, deposited in MNHN; MNHN 13/1970).

###### Other material examined.

No other material examined.

###### Diagnosis.

Male is distinguished from other “bourgini group” species by the distinctly shaped cephalon, that is triangular with a post-ocular pointed “head” (Fig. 18A), and also by the complete fusion of the sternum and carapace. Male is distinguished from other “bourgini group” species except *E. milloti* sp. n. and *E. lukemacaulayi* sp. n. (and presumably *E. milajaneae* sp. n., although the male is unknown) by the presence of a lateral process on the conductor (Figs 14G–I, 17D–I, 18F–G, asterisk), which in only *E. pauliani* is as wide as long.

###### Description.

Male holotype (MNHN 13/1970, from Andohahelo, Fort Dauphin, Madagascar). Total length 2.39, carapace 0.97 long, 0.82 wide. Abdomen 1.30 long, 1.40 high. Carapace tilt angle 62.38°, tilt height (CtH) 2.57, constriction 0.54, head length 1.22, neck length 1.29 (Fig. 2). CtH divided by carapace length 2.65. Cephalon with AME on large bulge, and with postocular pointed head (Fig. 18A–B). Cephalon with 4 small post-ocular spines at the crown, not on protrusions. Chelicerae 2.07 long, and with a short spine 0.31 from base of chelicerae that projects perpendicular to the cheliceral cuticle. Femur I 2.99 long. Sternum 0.41 long, 0.65 wide, but completely fused to the carapace; measurements were taken based on approximations of where the original sternum edge would have been. Carapace and sternum reddish brown with white setae, chelicera light reddish brown. Legs beige with reddish brown annulations on tibia and metatarsus. Abdomen beige, mottled with brown and with tufts of white setae (Fig. 18A). Pedipalpal bulb with a membranous sac above the base of the embolus, with a greatly exposed embolus that is encircled by the conductor. Conductor with basal part an uneven ridge shape (see the basal “c” in Fig. 18F–G), with distal part that is narrow as it encircles the embolus, with a large, broad, bulge before tapering off. Conductor with lateral process that is as wide as long (Fig. 18F–G, asterisk). Embolus broad but tapering at tip to a long, fine wire (Fig. 18I); the embolus tip is broken off of the left pedipalp (Fig. 18G, arrow), but is entire in the right pedipalp (Fig. 18I).

Female: The female paratype at MNHN was not available for examination.

###### Variation.

No other known material.

###### Natural history.

Specimens collected at 1800 m elevation.

###### Distribution.

Known only from the type locality, Andohahelo, in southeast Madagascar (Fig. 32). The distribution point in Fig. 32 is an approximation since the holotype label does not have latitude/longitude data.

###### Comments.

In *E. pauliani* the sternum is fused to the carapace, so the sternum measurements were based on an approximation of where the sternum border would be if this fusion was absent. In the type specimen the embolus tip is broken off in the left pedipalp (Fig. 18G – arrow), however the right pedipalp shows that the embolus tip is curved and much longer (Fig. 18I).

##### Eriauchenius ratsirarsoni

Taxon classificationAnimaliaAraneaeArchaeidae

(Lotz, 2003)

Figs 19 , 31

Archaea ratsirarsoni Lotz, 2003: 226, fig. 2A–C.

###### Type material.

Male holotype: as *Archaea ratsirarsoni* (Lotz, 2003), from MADAGASCAR, Toamasina, Forêt Clasée Sandranantitra, 18°2.9'S, 49°5.5'E, 450 m, 18–21 Jan 1999, rainforest, sifted litter, H.J. Ratsirarson (examined, deposited in CAS; CASENT9012339).

###### Other material examined.

MADAGASCAR, Toamasina: female paratype and 1juv, Ambatovy, 12.4 km NE Moramanga, 18°50'22"S, 48°18'30"E, 1080 m, 4–7 Mar 2007, montane rainforest, sifted litter, B.L. Fisher et al. (CASENT9028378); 1F,1juv, Parc National Mantadia, 18°47.5'S, 48°25.6'E, 895 m, 28 Nov – 1 Dec 1998, rainforest, sifted litter, H.J. Ratsirarson (CASENT9012338); 1M, Station Forestier Analamazaotra, administered by Mitsinjo, 0.75 km N Andasibe, 18°55.783’S, 48°24.696’E, 964m, 2 Feb 2009, primary montane rainforest, sifting litter around logs, dead fern fronds, and at base of traveler’s palm, H. Wood (USNMENT01377174); 1M,1F, Corridor Forestier Analamay-Mantadia, Ambohibolakely, 18°45'41"S, 48°21'52"E, 983m, 23-28 Nov 2012, rainforest, sifting litter, B.L. Fisher et al. (CASENT9062876).

###### Diagnosis.

Males and females are distinguished from other *Eriauchenius*, except *E. mahariraensis* and *E. sama* sp. n., by the presence of 4 small spines on the cephalon crown, and the cheliceral spine pointing perpendicular. Males of *E. ratsirarsoni* are distinguished from *E. sama* sp. n. and *E. mahariraensis* by lacking membranous tissue close to the conductor tip (Fig. 15D–L) and having a long conductor and conductor process that extends past the embolus tip (Fig. 19F, I). Females are distinguished by the presence of two lateral bulges on the FSGP and translucent “wings” (Fig. 19B, arrows).

###### Description.

Male holotype (CASENT9012339, from Forêt Clasée Sandranantitra, Madagascar). Total length 1.67, carapace 0.80 long, 0.74 wide. Abdomen 0.81 long, 0.87 high. Carapace tilt angle 67.5°, tilt height (CtH) 1.35, constriction 0.46, head length 0.75, neck length 0.64. CtH divided by carapace length 1.69. Cephalon with AME on a very small bulge, and with 4 small post-ocular spines (anterior two are broken off, rudimentary or missing) on the crown of the cephalon, and 1 spine between the LE and median eyes (on each lateral side, for a total of 2; broken off on the right side). Chelicerae 1.34 long, and with a long spine 0.20 from base of chelicerae that projects perpendicular. Femur I 1.58 long. Sternum 0.55 long, 0.37 wide. Carapace, chelicerae, sternum, and legs reddish brown with white setae; patellas I–IV, legs II–III, tarsi and metatarsi I–IV pale yellow. Abdomen mostly dark brown with light circular patches, but with several light-yellow patches; abdomen with white and brown setae. Pedipalpal bulb with a membraneous sac above the base of the embolus, with a greatly exposed embolus that is encircled by the conductor (Fig. 19D–L). Conductor swirls around the broad, dark embolus and has a large, long process on the prolateral side (Fig. 19D–I, c1); conductor and process extend past the tip of the embolus (Fig. 19F, I).

Female paratype (CASENT9028378). Total length 1.89, carapace 0.86 long, 0.76 wide. Abdomen 0.87 long, 1.07 high. Carapace tilt angle 69.8°, tilt height (CtH) 1.44, constriction 0.54, head length 0.73, neck length 0.67. CtH divided by carapace length 1.68. Cephalon with AME on a small bulge, and with 4 small spines (anterior pair is rudimentary) on the crown of the cephalon, and missing spine between the LE and median eyes. Chelicerae 1.41 long, and with small spine 0.19 from base of chelicerae projecting perpendicular. Femur I 1.44 long. Sternum 0.56 long, 0.39 wide. Carapace and sternum orangeish brown; legs and chelicerae pale yellow. Abdomen mostly light yellow but with several dark brown areas with light circular patches; abdomen with white and brown setae. Female genitalia FSGP with two large lateral bulges (Fig. 19B, arrows), and nearly translucent “wings”, with PB present (Fig. 19B); one group of poreplates on each side of the bursa anterior; poreplates heavily sclerotized and raised, forming a bulge on the bursa (Fig. 19C).

###### Variation.

Total length 1.67–1.70 (males; n=3), 1.70–2.47 (females; n=3); Carapace length 0.75–0.80 (males; n=3), 0.82–0.87 (females; n=3); Femur I 1.82–1.98 times the length of carapace in males (n=3), 1.63–1.75 in females (n=3); CtH divided by carapace length 1.65–1.69 in males (n=3), 1.68–1.70 in females (n=3). Average femur I length 1.47 in males (n=3) and 1.43 in females (n= 3). In all males the anterior pair of spines on the cephalon are either rudimentary or broken off; in females, one with anterior pair rudimentary, one with all 4 spines present, one with one anterior spine present and the other absent.

###### Natural history.

In montane and primary rainforest from 450–1080 m in elevation, collected by sifting litter.

###### Distribution.

Known only from central-eastern Madagascar (Fig. 31).

###### Nomenclatural remarks.

One male specimen (CASENT9062876) has a pedipalpal bulb that is more heavily sclerotized than the other specimens, and also has a blunt conductor tip (Fig. 19K–L, arrows) rather than the tapering tips in the other two specimens (Fig. 19J, arrow). All three male specimens occur in close proximity so this is likely not a case of geographic variation. This specimen may be a new species and future molecular work as well as additional collection of specimens from more localities can resolve this issue.

##### Eriauchenius rixi sp. n.

Taxon classificationAnimaliaAraneaeArchaeidae

http://zoobank.org/C0C320E4-C72E-466F-8A9F-B5B55B823B30

Figs 20 , 32

###### Type material.

Male holotype: Madagascar, Fianarantsoa, Parc National Ranomafana, Vohiparara, Piste Touristique, 21°13.6'S, 47°24.0'E, 1000 m, 19 Apr 1998, C. Griswold, D. Kavanaugh, N. Penny, M. Raherilalao, E. Rajeriarison, J. Ranorianarisoa, J. Schweikert, D. Ubick (deposited in CAS; CASENT9012013).

###### Other material examined.

Female paratype, same data as holotype, except 12 and 14 Apr 1998 (CASENT9012346).

###### Etymology.

The specific name is a patronym to honor Dr. Michael Rix for his work describing Australian archaeids and examining their biogeographic patterns and evolutionary relationships.

###### Diagnosis.

Males are distinguished from other “bourgini group” species by having a sharp process at the base of the pedipalpal tegulum (Fig. 20G–I, arrows). Females are distinguished from other “bourgini group” species by having the posterior bar curved towards the dorsal rather than the anterior (Fig. 20C), by having large broad “wings” and a narrow posterior elongation that is blunt at the end (Fig. 20C, arrow).

###### Description.

Male holotype (CASENT9012013, from Parc National Ranomafana, Madagascar). Total length 2.01, carapace 0.88 long, 0.79 wide. Abdomen 1.05 long, 1.23 high. Carapace tilt angle 64.8°, tilt height (CtH) 1.51, constriction 0.43, head length 0.80, neck length 0.71. CtH divided by carapace length 1.72. Cephalon with AME on large bulges, and with 4 short post-ocular spines on the crown, not on protrusions, and 1 short spine between the LE and median eyes (on each side, for a total of 2). Chelicerae 1.44 long, and with a short spine 0.34 from base of chelicerae. Femur I 1.99 long. Sternum 0.56 long, 0.34 wide. Carapace, chelicerae, and sternum dark reddish brown with white setae. Coxae and legs yellowish brown, with darker annulations on tibiae and metatarsi. Abdomen mottled brown and beige, with tufts of white setae (Fig. 20A). Pedipalpal tegulum with a sharp process (Fig. 20G–I, arrow), bulb with a membraneous sac on the retrolateral side, with conductor encircling the broad, thick embolus (Fig. 20D–L). SC present and long and thin (Fig. 20D–L). MA present and a dark horn-like process (Fig. 20G, I, J, L).

Female paratype (CASENT9012346). Total length 2.46, carapace 0.95 long, 0.86 wide. Abdomen 1.43 long, 1.82 wide. Carapace tilt angle 63.4°, tilt height (CtH) 1.82, constriction 0.50, head length 0.91, neck length 0.84. CtH divided by carapace length 1.92. Cephalon as in male. Chelicerae 1.62 long, and with short spine 0.37 from base of chelicerae. Femur I 2.06 long. Sternum 0.61 long, 0.37 wide. Colors as in male. Female genitalia FSGP with broad wings, a narrow, blunt posterior elongation (Fig. 20B, arrow); genitalia with a PB, and with poreplates in one group one each side of the bursa anterior (Fig. 20C).

###### Variation.

no other specimens known.

###### Natural history.

Specimens were collected at 1000 m in elevation, likely in rainforest, although not specified on the label.

###### Distribution.

Known only from Parc National Ranomafana in eastern Madagascar (Fig. 32).

##### Eriauchenius sama sp. n.

Taxon classificationAnimaliaAraneaeArchaeidae

http://zoobank.org/6F12A04B-D235-4613-A269-72FA930A99F4

Figs 21 , 31

###### Type material.

Male holotype: MADAGASCAR, Fianarantsoa, Parc National Ranomafana, Talatakely, 21°14.9'S, 47°25.6'E, 19–30 Apr 1998, C. Griswold, D. Kavanaugh, N. Penny, M. Raherilalao, J. Ranorianarisoa, J. Schweikert, D. Ubick (deposited in CAS: CASENT9012014).

###### Other material examined.

Female paratype, MADAGASCAR, Fianarantsoa, 29 km SSW Ambositra, Ankazomivady, 20°46.6'S, 47°09.9'E, 1700 m, 7 Jan 1998, forest, sifted leaf litter, B.L. Fisher (CASENT9012341).

###### Diagnosis.

Males and females are distinguished from other “bourgini group” species, except *E. ratsirarsoni* and *E. mahariraensis*, by having 4 spines on the cephalon, and the cheliceral spine pointing perpendicular. Males of *E. sama* sp. n. are also distinguished from other “bourgini group” species, by having a long process on the conductor that extends to the embolus tip (Fig. 21D–I, sclerite “c1”), and having the tip of the conductor point curving in a different direction than the more basal portion (Fig. 21G–I, arrow). Females are distinguished by the reduced FSGP, but the width divided by height is less than 2 (Fig. 21B).

###### Etymology.

The specific name is a noun in apposition; ‘sama’ means ‘pelican’ in Malagasy.

###### Description.

Male holotype (CASENT9012014, from Parc National Ranomafana, Madagascar). Total length 2.10, carapace 0.95 long, 0.85 wide. Abdomen 1.10 long, 1.26 high. Carapace tilt angle 72.5°, tilt height (CtH) 1.72, constriction 0.52, head length 0.82, neck length 0.81. CtH divided by carapace length 1.81. Cephalon with AME on a small bulge, and with 4 small post-ocular spines (although it is difficult to tell as the anterior pair is missing, rudimentary or broken off) on the crown of the cephalon, and 1 spine between the LE and AME (on each side, for a total of 2). Chelicerae 1.59 long, and with a spine 0.25 from base of chelicerae that projects perpendicular. Femur I 2.03 long. Sternum 0.63 long, 0.40 wide. Carapace, chelicerae, sternum, and legs reddish brown with white setae; patellas, tibias, and metatarsi light tan. Abdomen mottled with dark brown and light yellow patches, with light circular patches on the dark brown portions, with white setae. Pedipalpal bulb with a large membraneous sac above the base of the embolus, with a greatly exposed embolus that is encircled by the conductor (Fig. 21D–L). Conductor with a large bulge and a long process on the prolateral side that extends to the tip of the embolus (Fig. 21D–I, “c1” sclerite). In ventral view the left conductor curves in a clockwise direction, but the tip changes direction (Fig. 21H–I, arrow). Embolus broad and dark (Fig. 21G).

Female paratype (CASENT9012341). Total length 2.24, carapace 0.87 long, 0.80 wide. Abdomen 1.18 long, 1.48 high. Carapace tilt angle 67.5°, tilt height (CtH) 1.50, constriction 0.50, head length 0.78, neck length 0.73. CtH divided by carapace length 1.73. Cephalon with AME on a small bulge, and with 4 small post-ocular spines (one is broken off or missing) on the crown of the cephalon, and 1 spine between the LE and AME (on each side, for a total of 2). Chelicerae 1.54 long, and with small spine 0.29 from base of chelicerae. Femur I 1.82 long. Sternum 0.60 long, 0.40 wide. Cephalothorax colors as in male. Abdomen anterior light tan, and posterior dark brown with light circular patches; abdomen with white and brown setae. Female genitalia FSGP small and simple, with “wings” reduced (Fig. 21B); PB present; with poreplates in one group on each side of the bursa anterior (Fig. 21C).

###### Variation.

no other known material.

###### Natural history.

Female specimen was collected at 1700 m in elevation in forest by sifting litter.

###### Distribution.

Known only from central-eastern Madagascar (Fig. 31).

Nomenclatural remarks: The male holotype and female paratype of *E. sama* sp. n. occur in different areas. The male and female were associated based on body size and carapace shape. Future molecular work as well as additional collection of specimens from more localities can help resolve this issue.

##### Eriauchenius zirafy sp. n.

Taxon classificationAnimaliaAraneaeArchaeidae

http://zoobank.org/82260DF8-9503-4817-8BC7-CD808F8F891E

Figs 22 , 31

###### Type material.

Male holotype: MADAGASCAR, Toamasina, Parc National Masoala, 2 hour hike from Tompolo, 39 km SE Maroantsetra, 15°41'35.5"S, 49°58'22.5"E, 450 m, 15–17 Dec 2008, primary montane rainforest, general collecting day, beating vegetation, F. Alvarez-Padilla & H. Wood (deposited in USNM: USNMENT01377199).

###### Other material examined.

MADGASCAR: Female paratype, Toamasina, Mikira forest, 2.5 hour hike from Andaparaty, 29 km N Maroantsetra, 15°12'2.95"S, 49°36'55.0"E, 195m, 10–12 Dec 2008, primary montane rainforest, beating vegetation: 5–10 feet above ground, F. Alvarez-Padilla & H. Wood (USNMENT01377190); 1M, same data as holotype (CASENT9028315); 1M,1J, Toamasina, Parc National Masoala, Ambohitsitondroina, Mt., Ambanizana, 15°34'9.9"S, 50°00'12.3"E, 650–700 m, 26–27 Feb 2003, rainforest, general collecting night, D. Andriamalala, D. Silva, et al. DSD0007 (CASENT9015571).

###### Etymology.

The specific name is a noun in apposition; ‘zirafy’ means ‘giraffe’ in Malagasy.

###### Diagnosis.

*E. bourgini* and *E. zirafy* sp. n. are distinguished from other “bourgini group” species by having two large protrusions on the crown of the cephalon (Fig. 22A). Females of *E. bourgini* and *E. zirafy* sp. n. are distinguished by the presence of two sclerotized invaginations on the bursa (Fig. 22C, arrows). Males of *E. bourgini* and *E. zirafy* sp. n. are distinguished by having a conductor with 4 processes in *E. bourgini* (Fig. 13D–K) and 5 in *E. zirafy* sp. n (Fig. 22D–K). *Eriauchenius zirafy* sp. n. is distinguished from *E. bourgini* by lacking posterior extensions on coxae I (Fig. 13L), and by having a cluster of small bumps and pores on the conductor base of the male pedipalp (Fig. 22G–I,L, arrow).

###### Description.

Male holotype (USNMENT01377199, from Parc National Masoala, Madagascar). Total length 1.79, carapace 0.75 long, 0.70 wide. Abdomen 0.97 long, 0.81 high. Carapace tilt angle 59.5°, tilt height (CtH) 1.72, constriction 0.32, head length 0.86, neck length 0.89 (Fig. 2). CtH divided by carapace length 2.29. Cephalon with AME on large bulge. Cephalon with 4 small post-ocular spines on the crown of the cephalon, with the posterior pair on large protrusions and the anterior pair not on protrusions, and 1 small spine between the LE and AME (on each side, for a total of 2). Chelicerae 1.50 long, and with a short spine 0.68 from base of chelicerae, projecting downward. Femur I 1.89 long. Sternum 0.50 long, 0.32 wide. Carapace, chelicerae, and sternum dark reddish brown with white setae. Coxae and legs lighter brown, with darker annulations on tibiae and metatarsi. Abdomen mottled brown and beige, with a bright white patch on each lateral side, with tufts of white setae (Fig. 22A). Pedipalpal bulb with a small membranous sac above the embolus base, with the base of the conductor small and triangular with a cluster of small bumps and pores (Fig. 22G–I,L, arrow), and with the remainder of the conductor with 4 long processes as it wraps around the embolus, although one process is bifurcating (Fig. 22D,G–I, c3 and c4) for a total of five processes (Fig. 22D–I, c1–c5). The embolus is thick, contains two processes, and has membranous parts.

Female paratype (USNMENT01377190). This specimen is damaged due to fungus, rendering measurements and color descriptions of the abdomen impossible. Carapace 0.75 long, 0.70 wide. Carapace tilt angle 65.4°, tilt height (CtH) 1.72, constriction 0.31, head length 0.81, neck length 0.92. CtH divided by carapace length 2.29. Cephalon as in male. Chelicerae 1.66 long, and with small spine 0.73 from base of chelicerae. Femur I 1.90 long. Sternum 0.51 long, 0.33 wide. Carapace colors as in male. Female genitalia FSGP small and simple; unknown whether PB is present or absent due to specimen damage; bursa lacking poreplates and instead having a sclerotized invagination on each side of the bursa anterior (Fig. 22C, arrows).

###### Variation.

Total length 1.79–1.80 (males; n=3); Carapace length 0.72–0.75 (males; n=3); Femur I 2.54–2.72 times the length of carapace in males (n=3) and 2.54 times the length of the carapace in females (n=1). CtH divided by carapace length 2.29–2.44 in males (n=3). Average femur I length 1.96 (males; n=3).

###### Natural history.

Specimens were collected from 195–700 m in elevation in rainforest by beating vegetation and general collecting.

###### Distribution.

Known only from areas around Parc National Masoala in northeastern Madagascar (Fig. 31).

##### Genus Madagascarchaea gen. n.

Taxon classificationAnimaliaAraneaeArchaeidae

http://zoobank.org/041CF907-A633-440F-A0DE-EA875E1265DF

###### Type species.

*Archaea gracilicollis* Millot, 1948

###### Etymology.

The name refers to the Madagascan distribution and is feminine in gender.

###### Diagnosis.

Distinguished from all other archaeids by the presence of six protrusions, each with a small spine, on the crown of the cephalon, and by the presence of a retrolateral apophysis on the male pedipalpal patella.

###### Description.

See Wood (2008) for a complete description.

###### Included species.

14 described species *M. ambre* (Wood, 2008), *M. anabohazo* (Wood, 2008), *M. borimontsina* (Wood, 2008), *M. gracilicollis* (Millot, 1948), *M. griswoldi* (Wood, 2008), *M. halambohitra* (Wood, 2008), *M. jeanneli* (Millot, 1948), *M. lavatenda* (Wood, 2008), *M. legendrei* (Platnick, 1991), *M. namoroka* (Wood, 2008), *M. spiceri* (Wood, 2008), *M. tsingyensis* (Lotz, 2003), *M. vadoni* (Millot, 1948), *M. voronakely* (Wood, 2008), and 4 new species described here: *Madagascarchaea fohy* sp. n., *Madagascarchaea lotzi* sp. n., *Madagascarchaea moramora* sp. n., *Madagascarchaea rabesahala* sp. n

###### Distribution.

Madagascar

###### Discussion.

We elevate the “gracilicollis group” (Wood 2008) to genus level based on phylogenetic analysis that strongly supports two monophyletic clades, *Eriauchenius* and *Madagascarchaea* gen. n., that have diversified on Madagascar (Fig. 1) (Wood et al. 2015; Wood et al. 2012). These two genera are not sister clades, but instead *Afrarchaea* is sister to *Eriauchenius* in Wood et al (2015), and *Afrarchaea* is sister to *Madagascarchaea* gen. n. in Wood et al (2012). *Eriauchenius* and *Afrarchaea* are never recovered as sister clades.

**Figure 23.**
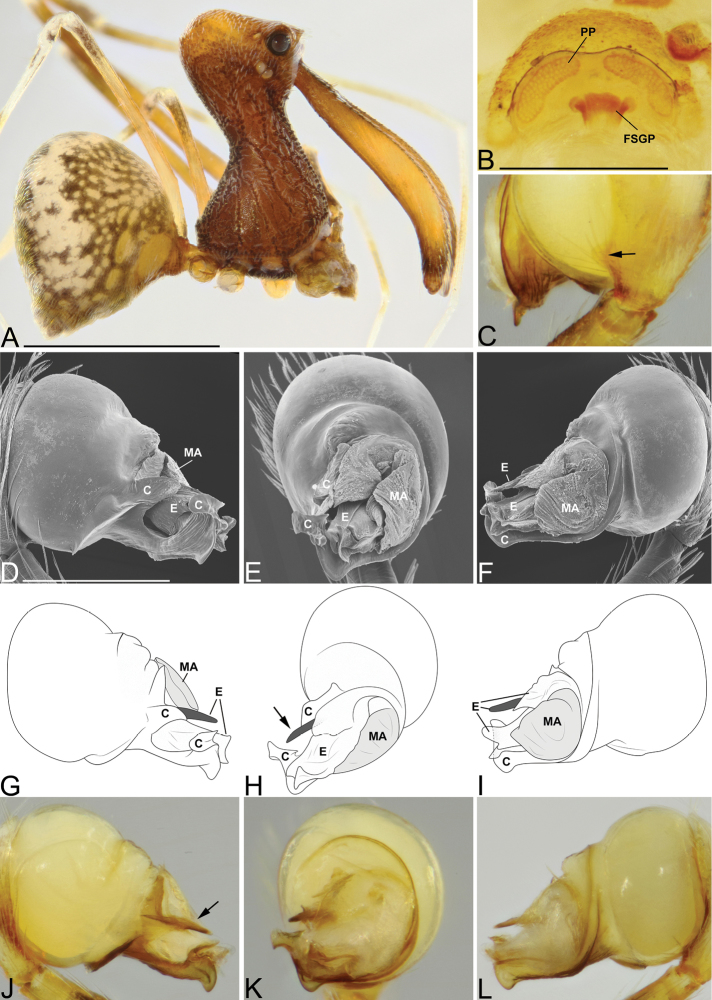
*Madagascarchaea fohy* sp. n. **A** male (holotype, CASENT9015766) habitus, lateral view **B** female (CASENT9015766) internal genitalia, dorsal view **C–L** male pedipalpal bulbs (holotype, CASENT9015766) **D–F** right bulb, image reversed **C, G–L** left bulb **C** cymbium close-up, dorsal view, arrow showing the retrolateral cymbium protrusion that has setae **D, G, J** prolateral view, arrow showing rod-like piece on embolus **E, H, K** ventral view, arrow showing rod-like piece on embolus **F, I, L** retrolateral view. Scale bars: 1 mm (**A**); 0.25 mm (**B, D**).

**Figure 24.**
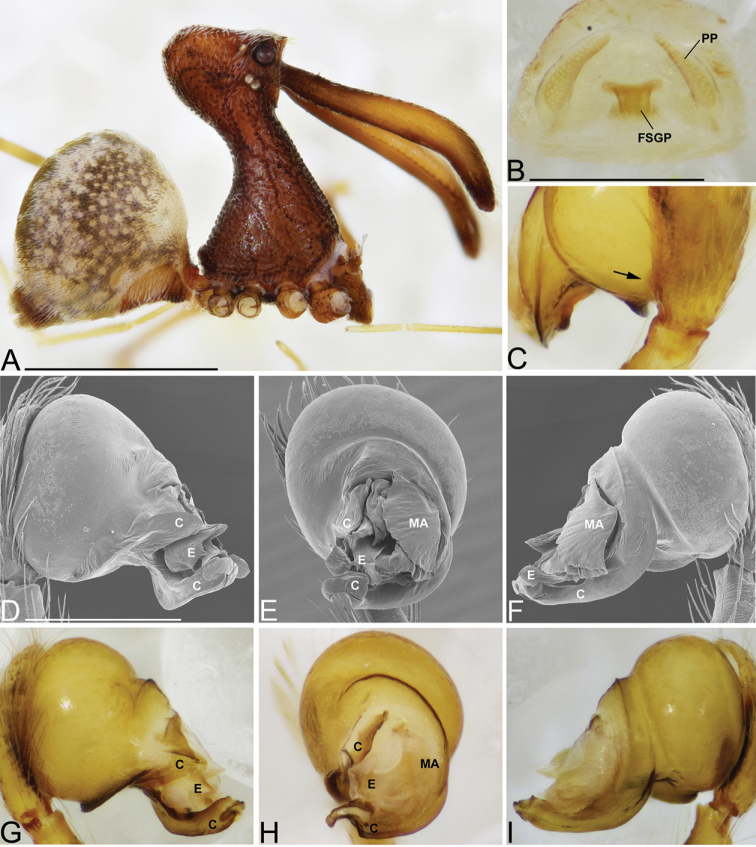
*Eriauchenius legendrei* (Platnick, 1991). **A** male (USNMENT01377217) habitus, lateral view **B** female (CASENT9012333) internal genitalia, dorsal view. **C–I** male pedipalpal bulbs **D–F** left bulb (CASENT9012333) **C** left bulb (CASENT9010074) **G–I** left bulb (USNMENT01377217): **C** cymbium close-up, dorsal view, arrow showing the small retrolateral cymbium protrusion that lacks setae **D, G** prolateral view **E, H** ventral view **F, I** retrolateral view. Scale bars: 1 mm (**A**); 0.25 mm (**B, D**).

**Figure 25.**
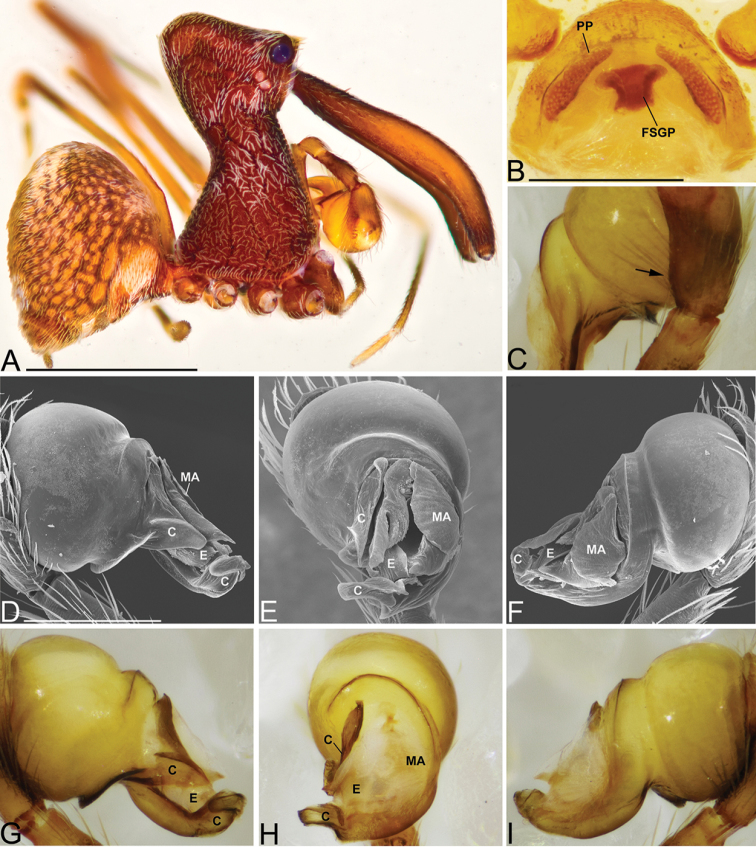
*Madagascarchaea rabesahala* sp. n. **A** male (holotype, CASENT9004011) habitus, lateral view, image reversed **B** female (CASENT9004086) internal genitalia, dorsal view **C–I** male pedipalpal bulbs **D–F** right bulb (CASENT9004086), image reversed **C, G–I** left bulb (CASENT9004086): **C** cymbium close-up, dorsal view, arrow showing the absence of a retrolateral cymbium protrusion **D, G** prolateral view **E, H** ventral view **F, I** retrolateral view. Scale bars: 1 mm (**A**); 0.25 mm (**B, D**).

*Madagascarchaea* gen. n. contains two main clades, the “vadoni group” and the “jeanneli group” (Fig. 1). The “vadoni group” contains *M. borimontsina*, *M. legendrei*, *M. vadoni*, *M. fohy* sp. n., and *M. rabesahala* sp. n., and these species share the following traits: a retrolateral apophysis on the male pedipalpal femur (compare fig. 12A with fig. 12B in Wood 2008) and a highly reduced FSGP that lacks “wings” (Figs 23B, 24B, 25B). The female genitalia of “vadoni group” species do not have interspecific variation. Instead, somatic traits are more useful for identifying “vadoni group” species. We split what was previously considered *M. legendrei* into two species: *M. legendrei* and *M. rabesahala* sp. n. For clarity, we describe both *M. legendrei* and *M. rabesahala* sp. n. here. In addition, there is a single female specimen (CASENT9046596), denoted as “*Madagascarchaea* sp. 1” in Fig. 1 that may be a new species, however, since this is the only known specimen and females are difficult to diagnose, we chose not to describe it (collection information: Madagascar, Fianarantsoa, Réserve Forestiére d’Agnalazaha, Mahabo, 42.9 km 215° SW Farafangana, 23°11'38"S, 47°43'23"E, 20 m, 19 Apr 2006, littoral rainforest, maxi winkler litter extraction, B.L. Fisher et al.).

**Figure 26.**
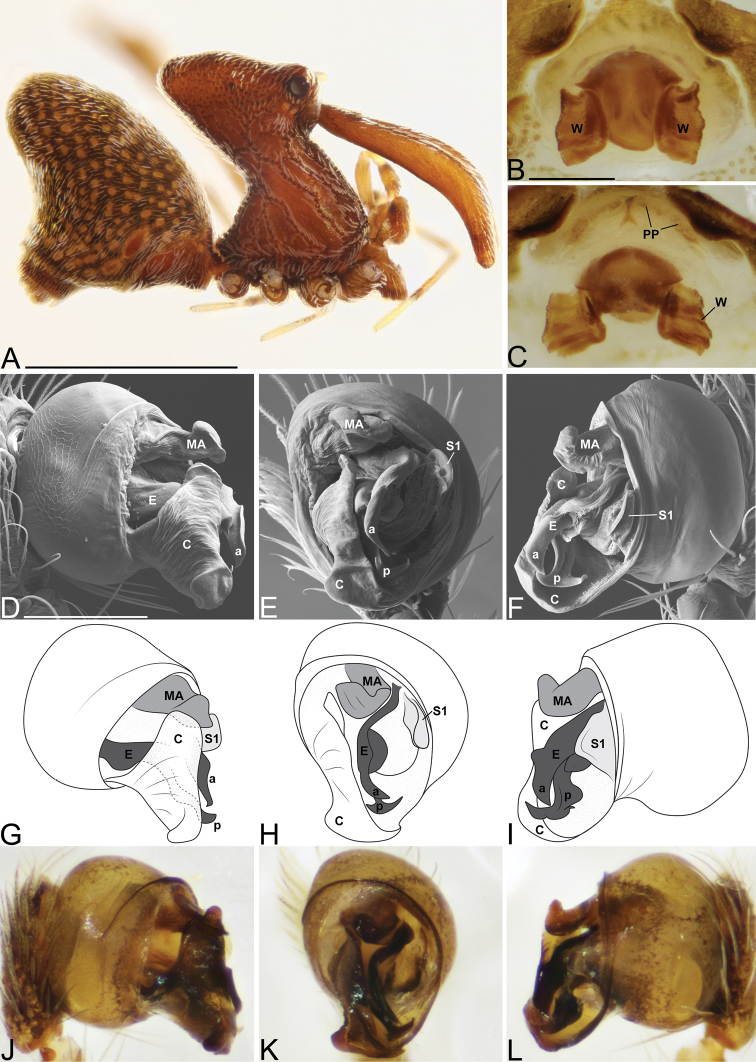
*Madagascarchaea jeanneli* (Millot, 1948). **A** male (CASENT9004085) habitus, lateral view, image reversed **B–C** female (CASENT9012336) internal genitalia **B** dorsal view **C** anterior view. **D–L** male pedipalpal bulbs **D–I** left bulb (CASENT9004085) **J–L** left bulb (CASENT9005233): **D, G, J** prolateral view **E, H, K** ventral view **F, I, L** retrolateral view. Scale bars: 1 mm (**A**); 0.125 mm (**B, D**).

**Figure 27.**
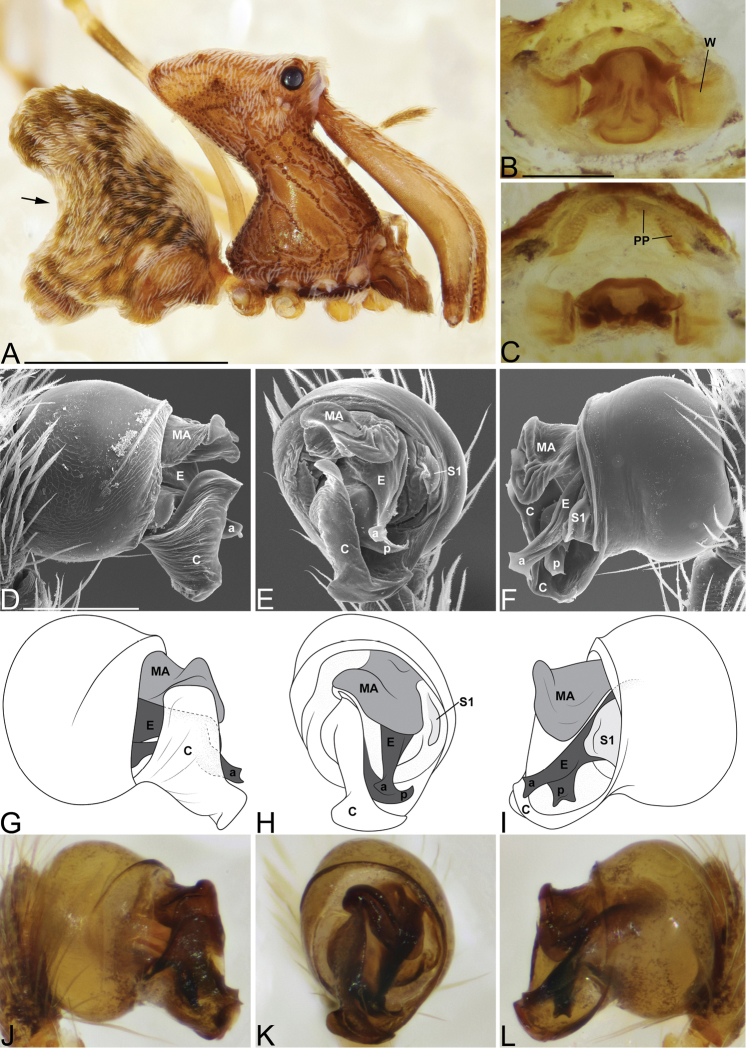
*Madagascarchaea lotzi* sp. n. **A** male (CASENT9018921) habitus, lateral view, arrow showing posterior invagination of abdomen **B–C** female (CASENT9012008) internal genitalia **B** dorsal view **C** anterior view **D–L** male pedipalpal bulbs **D–F** right bulb (CASENT9012004), image reversed, **G–L** left bulb (CASENT9012330) **D, G, J** prolateral view **E, H, K** ventral view **F, I, L** retrolateral view. Scale bars: 1 mm (**A**); 0.125 mm (**B, D**).

**Figure 28.**
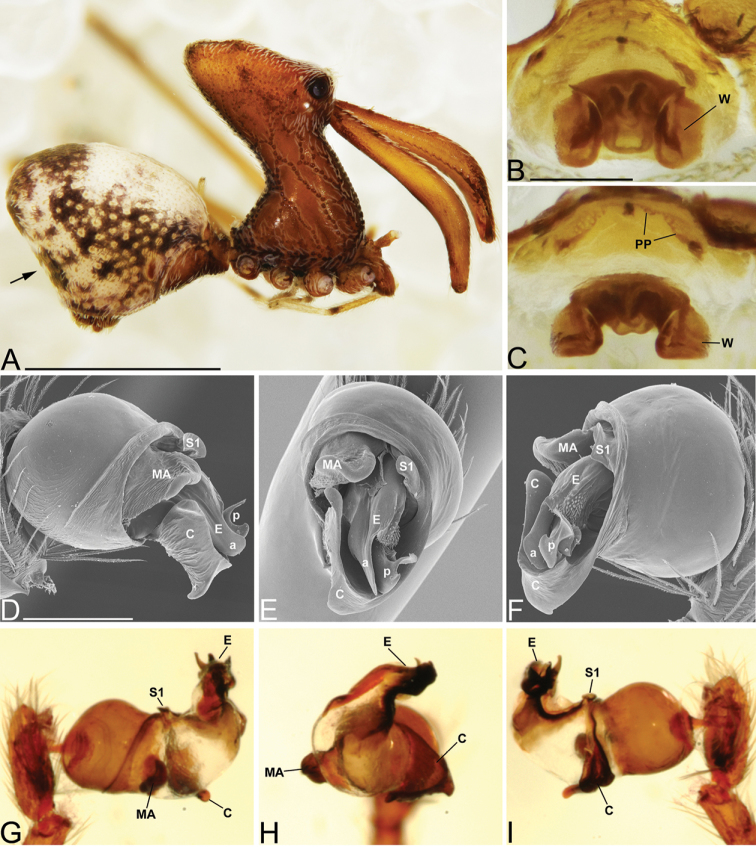
*Madagascarchaea moramora* sp. n. **A** male (holotype, USNMENT01377197) habitus, lateral view, image reversed, arrow showing abdomen posterior is straight. **B–C** female (USNMENT01377198) internal genitalia **B** dorsal view **C** anterior view **D–I** male pedipalpal bulbs (holotype, USNMENT01377197): **D–F** left bulb, **G–I** right bulb, expanded, image reversed **D, G** prolateral view **E, H** ventral view; **F, I** retrolateral view. Scale bars: 1 mm (**A**); 0.125 mm (**B, D**).

*M. jeanneli*, *M. ambre*, *M. lotzi* sp. n., and *M. moramora* sp. n. belong to the “jeanneli group” and are part of a species complex distinguished from other *Madagascarchaea* by having an elongated and pointy head (Figs 26A, 27A, 28A), a triangular abdomen that is either straight across the posterior (Fig. 28A) or invaginated (Figs 26A, 27A), and a conductor that is a large triangular piece in the prolateral view (Figs 26D, 27D). The current study splits the *M. jeanneli* of Wood (2008) into several different species: *M. jeanneli*, *M. lotzi* sp. n., and *M. moramora* sp. n. For clarity, we also redescribe *M. jeanneli* here. *M. ambre*, is considered a part of the “jeanneli-complex” but is not redescribed here, see Wood (2008) for a complete description. These four species form a monophyletic group (Fig. 1), although *M. jeanneli* is not included in the phylogeny. Females cannot be distinguished. For the males, embolus shape is the main morphological difference among species.

###### *Madagascarchaea* gen. n.: to supplement “Gracilicollis group” key

When using the identification key from Wood (2008), if a specimen is identified as either *M. jeanneli* or *M. ambre*, then use the following key to separate *M. jeanneli*, *M. ambre*, *M. lotzi* sp. n., and *M. moramora* sp. n.; females of these four species are indistinguishable:

**Table:** 

1	Embolus bifurcation shallow, both parts of bifurcation roughly equal thickness (Fig. 27E–F, G–H, “a” and “p”); posterior of abdomen usually invaginated in both males and females (Fig. 27H, arrow)	***M. lotzi***
–	Embolus with a deeper bifurcation, both parts of bifurcation different thickness (Figs 26F, I, L, 28E–F, compare shape of “a” with shape of “p”); abdomen invaginated or straight across the posterior in both sexes (Fig. 28A, arrow)	**2**
2	Anterior portion of embolus bifurcation thinner than the posterior part, and jutting out past the conductor in the ventral view (see fig. 21 in Wood 2008); usually abdomen straight across the posterior in both sexes (as in Fig. 28A, arrow; also see fig. 1C in Wood 2008)	***M. ambre***
–	Anterior portion of embolus bifurcation broad, posterior portion not extending past conductor, and posterior portion with a bifurcation (Fig. 26E–F,H–I)	**3**
3	Anterior portion of embolus bifurcation thick and blunt at tip (Fig. 28D–F, “a”), posterior portion with a bifurcation at tip with each piece of unequal width (Fig. 28D–F, “p”); posterior part of abdomen usually straight in both sexes (Fig. 28A, arrow)	***M. moramora***
–	Anterior portion of embolus bifurcation tapering off to a point (Fig. 26D–L, “a”), posterior portion with a bifurcation at the tip (Fig. 26E–F, H–I); posterior part of abdomen usually invaginated in both sexes (Fig. 26A)	***M. jeanneli***

When using the identification key from Wood (2008), if a specimen is identified as *M. legendrei*, then use the following key to separate species of *M. legendrei*, *M. fohy* sp. n., and *M. rabesahala* sp. n.:

**Table:** 

1	In males and females, cephalon crown rounded (Figs 23A, 24A)	**2**
–	Cephalon crown not as round in both sexes (Fig. 25A)	***M. rabesahala***
2	In males, pedipalpal bulb, with a sclerotized rod-shaped piece on embolus (Fig. 23H, J, arrow); presence of a cymbium process that has setae on the retrolateral side of the cymbium (Fig. 23C); females of *M. fohy* and *M. legendrei* are indistinguishable	***M. fohy***
–	Embolus lacking heavily sclerotized rod-shaped piece (Figs 24G–I, 25G–I); retrolateral process on cymbium present but lacking setae (Fig. 24C)	***M. legendrei***

#### “Vadoni group” new species and a redescription of *M. legendrei*

##### Madagascarchaea fohy sp. n.

Taxon classificationAnimaliaAraneaeArchaeidae

http://zoobank.org/6ACB2E06-A02F-46E2-879E-D88D40F9C556

Figs 23 , 33

###### Type material.

Male holotype: MADAGASCAR, Toamasina, Ivoloina Parque Zoologique, 12 km from Tamatave, 18°03'21.6"S, 49°21'32.5"E, 26m, 19 Feb 2003, disturbed rainforest, general collecting night, D. Andriamalala, D. Silva, et al. (deposited in CAS; CASENT9015766).

###### Other material examined.

MADGASCAR: Female paratype, same data as holotype (CASENT9015766); 1F,1juv, Toamasina, Parc National Masoala, Ambohitsitondroina Mt., Ambanizana, 15°34'19.5"S, 50°00'25"E, 900–950 m, 27 Feb 2003, rainforest, general collecting, D. Andriamalala, D. Silva, et al. (CASENT9015493).

###### Etymology.

The specific name is a noun in apposition; ‘fohy’ means ‘shorty’ in Malagasy.

###### Diagnosis.

Distinguished from all other archaeids, except *M. vadoni*, *M. legendrei*, *M. rabesahala* sp. n., and *M. borimontsina* (likely, because the male is unknown) by having a retrolateral apophysis on the distal side of both the palpal femur and patella (fig. 12B from Wood 2008), and because the FSGP is highly reduced and lacks “wings” (Figs 23B, 24B, 25B). *M. fohy* sp. n. is distinguished from *M. vadoni*, *M. legendrei*, and *M. rabesahala* sp. n. by having a heavily sclerotized rod-shaped sclerite on the palpal bulb (Fig. 23H, J, arrow), and by the presence of a retrolateral protrusion on the cymbium that has setae (Fig. 23C, arrow). *M. fohy* sp. n. is further distinguished from *M. vadoni* by having 6 spines that are not on protrusions on the cephalic crown, opposed to the small protrusions seen in *M. vadoni* (Wood, 2008: fig. 18A, C); from *M. borimontsina* by having less than 6 true teeth on the cheliceral retromargin (Wood 2008), by lacking a point on the AME bulge (Wood 2008: fig. 18D), and by lacking a cheliceral swelling on the posterior-basal side of the chelicerae (Wood 2008: fig. 18D).

###### Description.

Male holotype (CASENT9015766, from Ivoloina Parque Zoologique, Madagascar). Total length 1.61, carapace 0.68 long, 0.55 wide. Abdomen 0.85 long, 0.93 high. Carapace tilt angle 58.1°, tilt height (CtH) 1.29, constriction 0.33, head length 0.56, neck length 0.70. CtH divided by carapace length 1.91. Cephalon with AME on large bulge, and with 6 small post-ocular spines on the crown, not on protrusions, and 1 small spine between the LE and median eyes (on each side, for a total of 2). Chelicerae 1.40 long, and with a small spine 0.27 from base of chelicerae that projects downward (Fig. 23A). Femur I 1.96 long. Sternum 0.45 long, 0.27 wide. Carapace, chelicerae, sternum and legs reddish brown with white setae. Legs with darker annulations on tibiae and metatarsi. Abdomen mottled with dark brown areas and lighter yellowish areas, interspersed with white setae (Fig. 23A). Pedipalpal bulb of the “vadoni group” form (Fig. 23C–L): pedipalpal bulbs elongated with conductor swirling around a mostly membraneous embolus; conductor base triangular (Fig. 23D, G, see basal “c”), with remainder of conductor elongate and cradling embolus, and with a curved tip; MA translucent and fans out (Fig. 23F, I). Cymbium with retrolateral protrusion that has setae (Fig. 23C, arrow). Embolus with heavily sclerotized rod-like piece (Fig. 23H, J, arrow).

Female paratype (CASENT9015766). Total length 1.97, carapace 0.75 long, 0.63 wide. Abdomen 1.10 long, 1.38 high. Carapace tilt angle 54.2°, tilt height (CtH) 1.55, constriction 0.41, head length 0.73, neck length 0.82. CtH divided by carapace length 2.07. Cephalon as in male. Chelicerae 1.55 long, and with a short spine 0.28 from base of chelicerae and projecting downward (Fig. 23A). Femur I 2.04 long. Sternum 0.50 long, 0.29 wide. Colors as in male. Genitalia of the “vadoni-group” form (Fig. 23B): with one group of poreplates on each side of the bursa anterior, and FSGP highly reduced, lacking “wings.”

###### Variation.

Total length 1.67–1.97 (females; n=2); Carapace length 0.71–0.75 (females; n=2); Left and right femur I missing in one female (CASENT9015493). Femur I 2.73 times the length of carapace in females (n=1); CtH divided by carapace length 1.92–2.07 in females (n=2).

###### Natural history.

Specimens were collected in disturbed and undisturbed rainforest from 26–950 m in elevation by general collecting.

###### Distribution.

Known only from northeastern and central-eastern Madagascar (Fig. 33).

##### Madagascarchaea legendrei

Taxon classificationAnimaliaAraneaeArchaeidae

(Platnick, 1991) comb. n.

Figs 24 , 33

Archaea legendrei Platnick, 1991: 137, figs 7–14.
Eriauchenius legendrei (Platnick, 1991): Wood 2008: 286, figs 2E,12B,18A,20A–D (new combination).

###### Type material.

Male holotype: as *Archaea legendrei* Platnick, 1991: Madagascar, Fianarantsoa, 7 km W Ranomafana, 1100 m, 1–7 Nov 1988, montane rainforest, pyrethrin fogging of dead leaves on fallen tree, W.E. Steiner (deposited in USNM, examined; USNMENT00879968)

###### Other material examined.

MADAGASCAR: Female paratype, same data as holotype (USNMENT00879968); Fianarantsoa, Parc National Ranomafana: 2M,8F, 4 eggcases, Vohiparara, Sahamalaotra forest, 41.1 km 54° NE Fianarantsoa, 21°14'19.9"S, 47°23'39.2"E, 1200 m, 26 Dec 2005 – 14 Jan 2006, montane rainforest, general collecting day, beating vegetation: in clumps of dead dry foliage, H. Wood, J. Miller, J.J. Rafonomezantsoa, E. Rajeriarison, V. Andriamananony (USNMENT01377217, USNMENT01377218, USNMENT01377219, USNMENT01377210, USNMENT01377211); 2M,10F,16Juvs, 5 eggcases, Talatekely forest, 42.3 km 58° NE Fianarantsoa, 21°15'28.0"S, 47°25'21.8"E, 1050 m, 24 Dec 2005 – 14 Jan 2006, montane rainforest, general collecting day and night, H. Wood, J. Miller, J.J. Rafonomezantsoa, E. Rajeriarison, V. Andriamananony (USNMENT01377212, USNMENT01377213, USNMENT01377214, USNMENT01377205); 2M,4F,3Juvs,1 eggcase, Vohiparara, 3.6 km W Ranomafana, 21°14.243’S, 47°23.842’E, 1150 m, 13–14 Jan 2009, primary montane rainforest, general collecting day and night, C. Griswold, A. Saucedo and H. Wood (USNMENT01377206, USNMENT01377207, USNMENT01377208, USNMENT01377209); 1F, Talatakely, 21°14.9’S, 47°25.6’E, 5–18 Apr 1998, C. Griswold, D. Kavanaugh, N. Penny, M. Raherilalao, J. Ranorianarisoa, J. Schweikert, D. Ubick (CASENT9012347); 1M, same data as previous except 19–30 Apr 1998 (CASENT9010075); 2M,4F,3Juv, Vatoharanana, 21°16.7’S, 47°26.1’E, 1200 m, 15 Apr 1998, primary forest, C. Griswold, D. Kavanaugh, N. Penny, M. Raherilalao, J. Ranorianarisoa, J. Schweikert, D. Ubick (CASENT9012349, CASENT9010076); 1F,1juv, 2.3 km N Vohiparara village, 21°12.8’S, 47°23.0’E, 1100 m, 18 Apr 1998, C. Griswold, D. Kavanaugh, N. Penny, M. Raherilalao, E. Rajeriarison, J. Ranorianarisoa, J. Schweikert, D. Ubick (CASENT9012345); 1F, same data as previous except 10-11 Apr 1998 (CASENT9012348); 1M,2F, Vohiparara, Piste Touristique, 21°13.6’S, 47°24.0’E, 1000 m, 23 Apr 1998, C. Griswold, D. Kavanaugh, N. Penny, M. Raherilalao, E. Rajeriarison, J. Ranorianarisoa, J. Schweikert, D. Ubick (CASENT9012333); 1M, Talatakely, 21°15’S, 47°26’E, 915–1000 m, 30 Oct - 20 Nov 1998, V.F. Lee, K.J. Ribardo (CASENT9010074); 1M, Vatoharanana River, 4.1 km 231° SW Ranomafana, 21°17'24"S, 47°26'00"E, 1100 m, 27–31 Mar 2003, montane rainforest, EB20 yellow pan trap, Griswold, Fisher et al (CASENT9018908); 1M,2F, Talatakely, 21°15’S, 47°25’E, 900 m, 5–7 Dec 1993, N. Scharff, S. Larcher, C. Griswold, and R. Andriamasimanana (CASENT9046586); 1M,1F,2juvs, Trail FF, 14 May 1992, sifting litter 1 hr sample, B. Roth (CASENT9012334); 1F,1juv, 7 km W Ranomafana, 1100 m, 23–28 Feb 1990, montane rainforest, W.E. Steiner (USNMENT00879971); 1F, 7 km W Ranomafana, 21°12’S, 47°27’E, 1000 m, 1–7 Mar 1990, montane rainforest, W.E. Steiner (USNMENT00879987).

###### Diagnosis.

Distinguished from all other archaeids, except *M. vadoni*, *M. rabesahala* sp. n., *M. fohy* sp. n., and *M. borimontsina* (presumably, because the male is unknown) by having a retrolateral apophysis on the distal side of the male pedipalpal femur and patella (see fig. 12B in Wood 2008), and by the shape of the FSGP that is highly reduced and lacks “wings” (Fig. 24B). *M. legendrei* is distinguished from *M. vadoni* by having a rounded cephalon with the cephalic spines not on protrusions (Fig. 24A), rather than the larger protrusions seen in *M. vadoni* (compare figs 18A and 18C in Wood 2008); from *M. borimontsina* by having less than 6 teeth on the cheliceral retromargin, by having a rounded bulge that the AME rests upon, and by lacking a bulge on the posterio-basal side of the chelicerae (see fig. 18D in Wood 2008); from *M. fohy* sp. n. by lacking a heavily sclerotized rod-shaped sclerite on the palpal bulb (Fig. 23H,J, arrow), and by lacking setae on the retrolateral cymbial protrusion (Figs 23C, 24C, arrow); and from *M. rabesahala* sp. n. by having a more rounded cephalon, and by having a comparatively smaller and less sclerotized triangular basal portion of the conductor (compare basal “c” in Figs 24G, 25G).

###### Description.

Male (based on CASENT9012333, from Parc National Ranomafana, Madagascar). Total length 1.67, carapace 0.74 long, 0.58 wide. Abdomen 0.90 long, 1.13 high. Carapace tilt angle 61.9°, tilt height (CtH) 1.42, constriction 0.28, head length 0.58, neck length 0.84. CtH divided by carapace length 1.92. Cephalon with AME on large bulge, and with 6 short post-ocular spines at the crown, not on protrusions, and 1 short spine between the LE and AME (on each side, for a total of 2; see fig. 18A in Wood 2008). Chelicerae 1.53 long, and with a small spine 0.34 from base of chelicerae and projecting downward (Fig. 24A). Femur I 2.35 long. Sternum 0.49 long, 0.29 wide. Carapace, chelicerae, sternum and legs reddish brown with white setae. Legs with darker annulations on tibiae and metatarsi. Abdomen dark brown with lighter circular patches throughout, with white setae (Fig. 24A). Pedipalpal bulb of the “vadoni group” form (Fig. 24D–I): pedipalpal bulbs elongated with conductor swirling around a mostly membraneous embolus; conductor base triangular (Fig. 24D,G), with remainder of conductor elongate and cradling embolus, and with a curved tip; MA translucent and fans out (Fig. 24E–F, H–I). Cymbium with small retrolateral protrusion that lacks setae (Fig. 24C, arrow).

Female (based on CASENT9012333, from Parc National Ranomafana, Madagascar). Total length 1.92, carapace 0.79 long, 0.63 wide. Abdomen 1.13 long, 1.40 high. Carapace tilt angle 54.4°, tilt height (CtH) 1.63, constriction 0.36, head length 0.71, neck length 0.90. CtH divided by carapace length 2.06. Cephalon as in male. Chelicerae 1.61 long, and with short spine 0.34 from base of chelicerae and projecting downward (Fig. 24A). Femur I 2.26 long. Sternum 0.53 long, 0.31 wide. Colors as in male. Genitalia of the “vadoni-group” form (Fig. 24B): with one group of poreplates on each side of the bursa anterior, and with FSGP highly reduced, lacking “wings.”

###### Variation.

Total length 1.46–1.67 (males; n=5), 1.81–2.02 (females; n=5); Carapace length 0.66–0.77 (males; n=5), 0.76–0.82 (females; n=5); Femur I 3.01–3.66 times the length of carapace in males (n=5), 2.71–3.05 in females (n=5); CtH divided by carapace length 1.93–2.24 in males (n=5), 2.01–2.21 in females (n=5); Average femur I length 2.32 in males (n=5), 2.29 in females (n= 5).

###### Natural history.

Specimens were collected in montane rainforest from 915–1200 m in elevation by beating clumps of dead, dry foliage, by sifting litter, in yellow pan traps, and by general collecting day and night.

###### Distribution.

Known only from Parc National Ranomafana in central eastern Madagascar (Fig. 33).

##### Madagascarchaea rabesahala sp. n.

Taxon classificationAnimaliaAraneaeArchaeidae

http://zoobank.org/90FD4DB2-9E20-448F-BB80-1B614B975321

Figs 25 , 33

###### Type material.

Male holotype: MADAGASCAR, Antananarivo, 3 km 41° NE Andranomay, 11.5 km 147° SSE Anjozorobe, 18°28'24"S, 47°57'36"E, 1300 m, 5–13 Dec 2000, montane rainforest, beating and sweeping, Fisher, Griswold et al. (deposited in CAS: CASENT9004011).

###### Other material examined.

MADAGASCAR: Female paratype, same data as holotype except beating low vegetation (CASENT9003843); 1M, together with the holotype (CASENT9004011); 3M,3F,1juv, same data as holotype except general collecting (CASENT9004086); 1F,1juv,1 eggcase, Toamasina, Station Forestier Analamazaotra, administered by Mitsinjo, 0.75 km N Andasibe, 18°55.783’S, 48°24.696’E, 964m, 2 Feb 2009, primary montane rainforest, sifting litter around logs, dead fern fronds, and at base of traveler’s palm, H. Wood (USNMENT01377200).

###### Etymology.

The specific name is a noun in apposition and commemorates Gisèle Rabesahala, a Malagasy activist and politician.

###### Diagnosis.

Distinguished from all other archaeids, except *M. vadoni*, *M. legendrei*, *M. fohy* sp. n., and *M. borimontsina* (presumably, because the male is unknown) by having a retrolateral apophysis on the distal side of the male pedipalpal femur and patella (see fig. 12B in Wood 2008), and because the FSGP is highly reduced and lacks “wings” (Fig. 25B). *M. rabesahala* is distinguished from *M. vadoni* by having the cephalic spines not on protrusions (Fig. 24A), rather than the protrusions seen in *M. vadoni* (see fig. 18C in Wood 2008); from *M. borimontsina* by having less than 6 teeth on the cheliceral retromargin, by having a rounded bulge that the AME rests upon, and by lacking a bulge on the posterio-basal side of the chelicerae (see fig. 18D in Wood 2008); from *M. fohy* sp. n. by lacking a heavily sclerotized rod-shaped sclerite on the palpal bulb (Fig. 23H,J, arrow), and by lacking a retrolateral cymbial protrusion (Fig. 25C, arrow); and from *M. legendrei* by having a cephalon that is not as perfectly rounded (compare cephalon shape in Figs 24A, 25A), and by having a larger and more sclerotized triangular basal portion of the conductor (compare basal “c” in Figs 24G, 25G).

###### Description.

Male holotype (CASENT9004011, from forest close to Andranomay, Madagascar). Total length 1.66, carapace 0.73 long, 0.61 wide. Abdomen 0.90 long, 1.07 high. Carapace tilt angle 62.4°, tilt height (CtH) 1.42, constriction 0.39, head length 0.63, neck length 0.71. CtH divided by carapace length 1.95. Cephalon with AME on large bulge, and with 6 short post-occular spines at the crown, not on protrusions, and lacking spine between the LE and AME. Chelicerae 1.44 long, and with a small spine 0.33 from base of chelicerae and projecting downward (Fig. 25A). Femur I 2.01 long. Sternum 0.46 long, 0.30 wide. Carapace, chelicerae, sternum and legs reddish brown with white setae. Legs with darker annulations on tibiae and metatarsi. Abdomen dark brown with lighter circular patches throughout, with white setae (Fig. 25A). Pedipalpal bulb of the “vadoni group” form (Fig. 25D–I): pedipalpal bulbs elongated with the conductor swirling around a mostly membraneous embolus; conductor base triangular (Fig. 25D), with remainder of conductor elongate and cradling embolus, and with a curved tip; MA translucent and fans out (Fig. 25E–F,H–I). Cymbium lacks retrolateral protrusion (Fig. 25C, arrow).

Female paratype (CASENT903843). Total length 1.91, carapace 0.80 long, 0.67 wide. Abdomen 1.11 long, 1.55 high. Carapace tilt angle 62.3°, tilt height (CtH) 1.55, constriction 0.42, head length 0.78, neck length 0.82. CtH divided by carapace length 1.94. Cephalon as in male, except spine between LE and AME present. Chelicerae 1.61 long, and with a small spine 0.34 from base of chelicerae and projecting downward (Fig. 25A). Femur I 1.97 long. Sternum 0.49 long, 0.32 wide. Colors as in male. Genitalia of the “vadoni-group” form (Fig. 25B): with one group of poreplates on each side of the bursa anterior; with FSGP highly reduced, lacking “wings.”

###### Variation.

Total length 1.56–1.66 (males; n=5), 1.91–2.07 (females; n=4); Carapace length 0.72–0.75 (males; n=5), 0.74–0.81 (females; n=4); Femur I 2.69–2.86 times the length of carapace in males (n=5), 2.38–2.69 in females (n=4); CtH divided by carapace length 1.93–2.12 in males (n=5), 1.94–2.15 in females (n=4); Average femur I length 2.03 in males (n=5), 1.96 in females (n= 4). The spine between the LE and AME is present is 3 males out of 5, and 4 females out of 4.

###### Natural history.

Specimens were collected in montane rainforest from 960–1300 m in elevation by beating low vegetation, by sifting litter, by beating and sweeping, and by general collecting.

###### Distribution.

Known only from central eastern Madagascar (Fig. 33).

#### The new ‘pointy-head’ “vadoni group” species and a redescription of *M. jeanneli*

##### Madagascarchaea jeanneli

Taxon classificationAnimaliaAraneaeArchaeidae

(Millot, 1948) comb. n.

Figs 26 , 34

Archaea jeanneli Millot, 1948: 12, figs 1A,2B,3F.
Eriauchenius jeanneli (Millot, 1948): Wood 2008: 286, figs 2E,12B,18A,20A–D (new combination)

###### Type material.

2 females, 1 juv: as *Archaea jeanneli* Millot, 1948: Madagascar, La Mandraka (deposited in MNHN, examined; MNHN 13/1970)

###### Other material examined.

MADAGASCAR: 2M,1F, Toamasina, Res. Analamazaotra, Parc National Andasibe, 23 road km E Moramanga, 18°56'38.2"S, 48°25'03.2"E, 960 m, 16–18 Jan 2003, rainforest, general collecting night, C. Griswold, D. Silva, and D. Andriamalala (CASENT9005233, CASENT9018315); 7M,4F,1juv, Antananarivo, 3 km 41° NE Andranomay, 11.5 km 147° SSE Anjozorobe, 18°28'24"S, 47°57'36"E, 1300 m, 5–13 Dec 2000, montane rainforest, general collecting, Fisher, Griswold et al. (CASENT9004085); 3M,4F,1juv, same data as previous except beating and sweeping (CASENT9004009); 6F, Toamasina, Parc National Perinet, nr Andasibe, 18°56’S, 48°24’E, 1000 m, 4–5 Nov 1993, J. Coddington, S. Larcher, C. Griswold, R. Andriamasimanana, and N. Scharff (CASENT9012329, CASENT9046592, CASENT9046601); 1M, Toamasina, Parc National Perinet, 18°55’S, 48°25’E, 1-3 Aug 1992, V. & B. Roth (CASENT9012003); 2F, Toamasina, Station Forestier Analamazaotra, administered by Mitsinjo, 0.75 km N Andasibe, 18°55.783’S, 48°24.696’ E, 964m, 31 Jan – 3 Feb 2009, primary montane rainforest, hand collected at night in vegetation, C. Griswold, A. Saucedo and H. Wood (USNMENT01377201, USNMENT01377202); 1F, Antananarivo, Réserve Spéciale d’Ambohitantely, Forêt d’Ambohitantely, 20.9 km 72° NE Ankazobe, 18°13'31"S, 47°17'13"E, 1410 m, 17–22 Apr 2001, montane rainforest, EB17 beating low vegetation, Fisher, Griswold et al. (CASENT9012336); 2M,2F,1juv, Antananarivo, 7 km SE Andasibe Parc National (=Perinet) 18°58’S, 48°27’E, 5 Sep 2001, montane forest, beating foliage, D. Ubick (CASENT9001265).

###### Diagnosis.

Distinguished from all *Madagascarchaea*, except other ‘pointy head’ species *M. ambre*, *M. lotzi*, and *M. moramora* sp. n., by having a conductor that is a concave triangular shape (Fig. 26D–E, J–H, J–K), and by having an abdomen that is invaginated across the back (Fig. 26A), rather than rounded (as in Fig. 23A). Typically *M. jeanneli* can be distinguished from *M. ambre* and *M. moramora* sp. n., by having an abdomen that is invaginated in the posterior (Fig. 26A), rather than straight (Fig. 28A). *M. jeanneli* is further distinguished from *M. moramora* by having the anterior portion of the embolus taper off to a point (Fig. 26G–I, “a”) rather than being broad and blunt (Fig. 28D–F, “a”), and from *M. ambre* by having the anterior piece of the embolus broad and curved (Fig. 26G–I, “a”) and not straight, narrow, and jutting out past the conductor in the retrolateral direction (see fig. 21 in Wood 2008). *M. jeanneli* is distinguished from *M. lotzi* sp. n. by having an embolus with a very deep bifurcation (Fig. 26I, L), and with the posterior portion of the embolus having an additional bifurcation at the tip (Fig. 26F, I, “p”).

###### Description.

Male (based on CASENT9012003, from Parc National Perinet, Madagascar). Total length 1.55, carapace 0.64 long, 0.47 wide. Abdomen 0.79 long, 0.91 high. Carapace tilt angle 50.2°, tilt height (CtH) 1.19, constriction 0.35, head length 0.68, neck length 0.59. CtH divided by carapace length 1.85. Cephalon with AME on large bulge, and with 6 short post-occular spines at the crown, not on protrusions, and 1 short spine between and posterior to the LE and median eyes (see fig. 18B in Wood 2008). Chelicerae 1.18 long, and with a small spine 0.30 from base of chelicerae and projecting downward (Fig. 26A). Femur I 1.96 long. Sternum 0.44 long, 0.25 wide. Carapace, chelicerae, and sternum reddish brown with white setae. Legs light brown with darker annulations throughout. Abdomen dark brown with lighter circular patches throughout, with white and brown setae (Fig. 26A). Posterior edge of abdomen invaginated (Fig. 27A, arrow). Conductor concave and triangular; MA dark, thick, and curves anteriorad; S1 a thin ridge; embolus dark, with deep bifurcation, anterior portion curved and tapering off and posterior portion with additional bifurcation at tip with each side tapering off (Fig. 26D–L).

Female (based on CASENT9012336, from Réserve Spéciale d’Ambohitantely, Madagascar). Total length 2.00, carapace 0.78 long, 0.59 wide. Abdomen 1.18 long, 1.49 high. Carapace tilt angle 60.0°, tilt height (CtH) 1.53, constriction 0.41, head length 0.94, neck length 0.73. CtH divided by carapace length 1.97. Cephalon as in male. Chelicerae 1.48 long, and with a short spine 0.36 from base of chelicerae and projecting downward. Femur I 2.31 long. Sternum 0.51 long, 0.28 wide. Colors as in male. Posterior edge of abdomen invaginated. Genitalic bursa divided down the middle by sclerotized piece on the anterior-ventral side, with a few clusters of poreplates on either side; FSGP with two strong points arising from either side of anterior edge, having ‘wings’, and lacking posterior elongation (Fig. 26B–C).

###### Variation.

Total length 1.54–1.83 (males; n=5), 1.82–2.00 (females; n=5); Carapace length 0.64–0.76 (males; n=5), 0.74–0.78 (females; n=5); Femur I 2.60–3.06 times the length of carapace in males (n=5), 2.88–3.12 in females (n=5); CtH divided by carapace length 1.72–1.85 in males (n=5), 1.75-2.01 in females (n=5); Average femur I length 2.01 in males (n=5), 2.23 in females (n= 5).

###### Natural history.

Specimens were collected in montane rainforest from 960–1300 m in elevation by beating low foliage, low vegetation, by beating and sweeping, by general collecting at night, and by hand collecting at night in vegetation.

###### Distribution.

Known only from central eastern Madagascar (Fig. 34).

###### Nomenclature remarks.

Previous work (Wood 2008) lumped into *M. jeanneli* what we are here calling several different species. The *M. jeanneli* syntypes include two females, and females of the “jeanneli complex “(*M. ambre*, *M. jeanneli*, *M. lotzi* sp. n., and *M. moramora* sp. n.) cannot be confidently distinguished. However, the type locality of “La Mandraka” is an area about 40 kilometers east of Antananarivo, and so we propose that the female syntypes are conspecific to the males found in this area. The male and female specimens that were described and illustrated as *M. jeanneli* in Wood 2008 are here being called *M. lotzi* n. sp.

##### Madagascarchaea lotzi sp. n.

Taxon classificationAnimaliaAraneaeArchaeidae

http://zoobank.org/A6786589-292C-495B-8AA6-76D4371B6EE8

Figs 27 , 34

Eriauchenius jeanneli (Millot, 1948): Wood 2008: 283, figs 2C, 14E, 17A, 18B, 22A–D (in part; specimens from Parc National Ranomafana)

###### Type material.

Male holotype: MADAGASCAR, Fianarantsoa, Parc National Ranomafana, 2.3 km N Vohiparara village, 21°12.8’S, 47°23.0’E, 1100 m, 18 Apr 1998, C. Griswold, D. Kavanaugh, N. Penny, M. Raherilalao, E. Rajeriarison, J. Ranorianarisoa, J. Schweikert, D. Ubick (deposited in CAS; CASENT9012000).

###### Other material examined.

MADAGASCAR, Fianarantsoa, Parc National Ranomafana: 3M, 7F, including female paratype, Talatakely, 21°15’S, 47°25’E, 900 m, 5–7 Dec 1993, N. Scharff, S. Larcher, C. Griswold, and R. Andriamasimanana (CASENT9012330, CASENT9046572); 1F, same data as holotype (CASENT9012007); 1F, Vohiparara, Piste Touristique, 21°13.6’S, 47°24.0’E, 1000 m, 23 Apr 1998, C. Griswold, D. Kavanaugh, N. Penny, M. Raherilalao, E. Rajeriarison, J. Ranorianarisoa, J. Schweikert, D. Ubick (CASENT9012009); 1F, same data as previous except 19 Apr 1998 (CASENT9012008); 1F,1juv, same data as previous except 12,14 Apr 1998 (CASENT9012002); 3M, 4F, 1juv, 21°12’S, 47°27’E, Apr-May 1992, B Roth (CASENT9012005); 3M, 1F, 6juv Vohiparara, Sahamalaotra forest, 41.1 km 54° NE Fianarantsoa, 21°14'19.9"S, 47°23'39.2"E, 1200 m, 26 Dec – 14 Jan 2006 montane rainforest, general collecting day, beating vegetation – specifically clumps of dead dry foliage, H. Wood, J. Miller, J.J. Rafonomezantsoa, E. Rajeriarison, V. Andriamananony (USNMENT01377203, USNMENT01377204, USNMENT01377195); 1M, Vatoharanana, 21°16.7’S, 47°26.1’E, 1200 m, 15 Apr 1998, primary forest, C. Griswold, D. Kavanaugh, N. Penny, M. Raherilalao, J. Ranorianarisoa, J. Schweikert, D. Ubick (CASENT9012006); 1F, Vatoharanana River, 4.1 km 231° SW Ranomafana, 21°17'24"S, 47°26'00"E, 1100 m, 27–31 Mar 2003, montane rainforest, general collecting, beating and puffing spiders, Griswold, Fisher et al. (CASENT9018921); 1M, Talatakely, 21°14.9’S, 47°25.6’E, 5–18 Apr 1998, at night, C. Griswold, D. Kavanaugh, N. Penny, M. Raherilalao, J. Ranorianarisoa, J. Schweikert, D. Ubick (CASENT9012004); 1F,1juv, Vohiparara, 3.6 km W Ranomafana, 21°14.243’S, 47°23.842’E, 1150 m, 13–14 Jan 2009, primary montane rainforest, beating vegetation, C. Griswold, A. Saucedo and H. Wood (USNMENT01377196); 1M, 7 km SW Ranomafana, 1200 m, 22 Oct 1988, W. Steiner, C. Kremen, R. Van Epps (USNMENT00879969); 1F, 7 km W Ranomafana, 1100 m, 1–7 Nov 1988, W.E. Steiner (USNMENT00879988).

###### Etymology.

The specific name is a patronym to honor Dr. Leon Lotz for his work in describing the South African and Madagascan archaeids.

###### Diagnosis.

Typically *M. lotzi* sp. n. can be distinguished from the northern species, *M. ambre* and *M. moramora* sp. n., by the presence of an invagination in the abdomen posteriorly (Fig. 27A, arrow). *M. lotzi* sp. n. is further distinguished from *M. moramora* sp. n. by having a bifurcation in the embolus that is more shallow (Fig. 27E–F, H–I) rather than the deeper bifurcation of *M. moramora* sp. n. (Fig. 28E, F), and from *M. ambre* by having the anterior portion of the embolus not narrow and jutting out past the conductor in the retrolateral direction (see fig. 21 in Wood 2008). *M. lotzi* sp. n. is distinguished from *M. jeanneli* by having an embolus with a more shallow bifurcation, and with the posterior portion of the embolus lacking a large bifurcation at the tip (Fig. 27E–F, H–I).

###### Description.

Male holotype (CASENT9012000, from Parc National Ranomafana, Madagascar). Total length 1.61, carapace 0.72 long, 0.51 wide. Abdomen 0.88 long, 1.00 high. Carapace tilt angle 57.0°, tilt height (CtH) 1.24, constriction 0.40, head length 0.79, neck length 0.62. CtH divided by carapace length 1.74. Cephalon with AME on large bulge, and with 6 short post-occular spines at the apex, not on protrusions, and 1 short spine between and posterior to LE and median eyes (see fig. 18B of Wood 2008; broken off on the left side). Chelicerae 1.19 long, and with a small spine 0.33 from base of chelicerae and projecting downward (Fig. 27A). Femur I 1.94 long. Sternum 0.47 long, 0.25 wide. Carapace, chelicerae, and sternum reddish brown with white setae. Legs light brown with darker annulations throughout. Abdomen dark brown with lighter circular patches throughout, with white and brown setae (Fig. 27A). Posterior edge of abdomen invaginated (Fig. 27A, arrow). Conductor concave and triangular; MA dark, thick, and curves anteriorad; S1 a thin ridge; Embolus dark, with bifurcation, with both anterior and posterior pieces of bifurcation flat and wavy (Fig. 27D–L).

Female paratype (CASENT9012330). Total length 1.96, carapace 0.75 long, 0.55 wide. Abdomen 1.13 long, 1.64 high. Carapace tilt angle 55.0°, tilt height (CtH) 1.35, constriction 0.37, head length 0.75, neck length 0.61. CtH divided by carapace length 1.80. Cephalon as in male. Chelicerae 1.31 long, and with a short spine 0.33 from base of chelicerae and projecting downward. Femur I 1.99 long. Sternum 0.49 long, 0.27 wide. Colors as in male. Posterior edge of abdomen invaginated. Genitalic bursa divided down middle by sclerotized piece on anterior-ventral side, with two main groups of poreplates on either side (Fig. 27C); FSGP with two strong points arising from either side of anterior edge, having ‘wings’ and lacking posterior elongation (Fig. 27B).

###### Variation.

Total length 1.53–1.61 (males; n=5), 1.70–1.96 (females; n=5); Carapace length 0.68–0.74 (males; n=5), 0.73–0.80 (females; n=5); Femur I 2.64–2.71 times the length of carapace in males (n=5), 2.66–2.89 in females (n=5); CtH divided by carapace length 1.64–1.74 in males (n=5), 1.62–2.80 in females (n=5); Average femur I length 1.90 in males (n=5), 2.07 in females (n= 5).

###### Natural history.

Specimens were collected in montane rainforest from 900–1200 m in elevation by general collecting, beating and puffing, general collecting at night, beating vegetation, and beating clumps of dead, dry foliage.

###### Distribution.

Known only from Parc National Ranomafana in central eastern Madagascar (Fig. 34).

##### Madagascarchaea moramora sp. n.

Taxon classificationAnimaliaAraneaeArchaeidae

http://zoobank.org/E3638C1C-D339-4E3D-A5C3-C1BEB7DC90FE

Figs 28 , 34

###### Type material.

Male holotype: MADAGASCAR, Toamasina, Mikira forest, 2.5 hour hike from Andaparaty, 29 km N Maroantsetra, 15°12'2.95"S, 49°36'55.0"E, 195m, 10–12 Dec 2008, primary montane rainforest, general collecting day, F. Alvarez-Padilla & H. Wood (deposited in USNM; USNMENT01377197).

###### Other material examined.

MADAGASCAR: female paratype, same data as holotype except beating vegetation 5–10 feet above ground (USNMENT01377198).

###### Etymology.

The specific name is a noun in apposition; ‘mora mora’ means ‘easy easy’ in Malagasy.

###### Diagnosis.

*M. moramora* sp. n. can be distinguished from the southern species, *M. jeanneli* and *M. lotzi* sp. n., by the abdomen being straight across the posterior (Fig. 28A, arrow) rather than invaginated (Figs 26A, 27A). *M. moramora* sp. n. is further distinguished from *M. lotzi* sp. n. by having a deep embolus bifurcation (Fig. 28E–F) compared to the shallow bifurcation of *M. lotzi* sp. n. (Fig. 27E–F, H–I), and from *M. ambre* by having the anterior portion of the embolus bifurcation not narrow and jutting out past the conductor in the retrolateral direction (see fig. 21 in Wood 2008). *M. moramora* sp. n. is distinguished from *M. jeanneli* by having the anterior portion of the embolus bifurcation broad and blunt (Fig. 28F), rather than tapering (Fig. 26F, I), and having the posterior portion with a bifurcation at the tip with one side narrower than the other (Fig. 28D–E), whereas in *M. jeanneli* the bifurcation at the tip is equal sized on each side (Fig. 26F, I).

###### Description.

Male holotype (USNMENT01377197, from Mikira Forest, Madagascar). Total length 1.53, carapace 0.67 long, 0.46 wide. Abdomen 0.83 long, 0.92 high. Carapace tilt angle 57.2°, tilt height (CtH) 1.25, constriction 0.35, head length 0.73, neck length 0.63. CtH divided by carapace length 1.86. Cephalon with AME on large bulge, and with 6 short post-occular spines at the apex, not on protrusions, and 1 short spine between and posterior to the LE and median eyes (see fig. 18B in Wood 2008). Chelicerae 1.21 long, and with a small spine 0.28 from base of chelicerae and projecting downward (Fig. 28A). Femur I 1.86 long. Sternum 0.44 long, 0.24 wide. Carapace, chelicerae, and sternum reddish brown with white setae. Legs light brown with darker annulations throughout. Abdomen mottled with dark brown areas and lighter whitish areas, anterior of abdomen with large white patch, abdomen interspersed with white and brown setae (Fig. 28A). Posterior edge of abdomen straight and not invaginated (Fig. 28A, arrow). Conductor concave and triangular; MA dark, thick, and curves anteriorad; S1 present as a thin ridge; Embolus dark, with a deep bifurcation, with anterior portion broad and blunt at the tip, and with posterior portion with bifurcation at the tip, of which both sides are curved but one side is more narrow (Fig. 28D–F).

Female paratype (USNMENT01377198). Total length 1.98, carapace 0.79 long, 0.52 wide. Abdomen 1.09 long, 1.35 high. Carapace tilt angle 48.2°, tilt height (CtH) 1.48, constriction 0.35, head length 0.82, neck length 0.70. CtH divided by carapace length 1.87. Cephalon as in male. Chelicerae 1.42 long, and with a short spine 0.38 from base of chelicerae and projecting downward. Femur I 12.25 long. Sternum 0.49 long, 0.26 wide. Colors as in male. Posterior edge of abdomen straight and not invaginated. Genitalic bursa divided down middle by a sclerotized piece on the anterior-ventral side, with several groups of poreplates on either side; FSGP with two strong points arising from either side of anterior edge, having ‘wings’, and lacking posterior elongation (Fig. 28B–C).

###### Variation.

no other specimens known.

###### Natural history.

Specimens were collected in montane rainforest at 925 m in elevation by general collecting in the day and by beating vegetation 5–10 feet above ground.

###### Distribution.

Known only from Mikira Forest in northeastern Madagascar (Fig. 34).

###### Nomenclature remarks.

We could not determine whether several female specimens were *M. ambre* or *M. moramora* sp. n. (Fig. 34). Locality information for these specimens is as follows, all MADAGASCAR: 1F, Antsiranana, Marojejy Reserve, 8.4 km NNW Manantenina, 14°26’S, 49°45’E, 700 m, 10–16 Nov 1993, C. Griswold, J. Coddington, N. Scharff, S. Larcher, R. Andriamasimanana (CASENT9012001); 1F, Toamasina, Parc National Masoala, Ambohitsitondroina Mt., Ambanizana, 15°34'9.9"S, 50°00'12.3"E, 700–750 m, 28 Feb 2003, rainforest, beating vegetation, D. Andriamalala, D. Silva, et al. (CASENT9015316); 2F, same as previous data except 600–650 m, 1-2 Mar 2003, general collecting night (CASENT9015372).

##### Genus Afrarchaea

Taxon classificationAnimaliaAraneaeArchaeidae

Forster & Platnick, 1984

Afrarchaea Forster & Platnick, 1984: 24.

###### Type species.

*Archaea godfreyi* Hewitt, 1919: 196, figs 1–2 (by original designation).

###### Diagnosis.

Distinguished from all extant genera by the presence of a prominent keel on the FSGP (see fig. 58 of Forster and Platnick (1984), fig. 3 of Lotz (2006), and figs 1c–d and 6b–c of Lotz (1996)). The keel likely has been lost in *A. royalensis* and *A. ngomensis* given the central phylogenetic placement of these two species (Fig. 1).

###### Description.

See Forster and Platnick (1984) for description.

###### Included species.

12 described species, *A. cornutus* (Lotz, 2003), *A. ansieae* Lotz, 2015, *A. bergae* Lotz, 1996, *A. entabeniensis* Lotz, 2003, *A. fernkloofensis* Lotz, 1996, *A. godfreyi* (Hewitt, 1919), *A. haddadi* Lotz, 2006, *A. harveyi* Lotz, 2003, *A. kranskopensis* Lotz, 1996, *A. lawrencei* Lotz, 1996, *A. ngomensis* Lotz, 1996, *A. royalensis* Lotz, 2006, *A. woodae* Lotz, 2006. Two species originally described as *Afrarchaea* have been transferred: *A. fisheri* Lotz, 2003 and *A. mahariraensis* Lotz, 2003, both to *Eriauchenus* (new combinations).

###### Distribution.

South Africa.

###### Discussion.

Phylogenetic analysis based on molecular data recovered a monophylectic *Afrarchaea* with strong branch support (Wood et al. 2015). This research supports the transfer of *A. cornutus* from *Eriauchenius* to *Afrarchaea* and the transfer of *E. mahariraensis* from *Afrarchaea* to *Eriauchenius* (Fig. 1). Furthermore, females of *A. cornutus* have a FSGP keel typical of *Afrarchaea* (see fig. 3 of Lotz 2006) and females of *E. mahariraensis* do not (Fig. 15B–C).

Regarding *Afrarchaea* distribution, Legendre (1970) reported on a male and female specimen (not examined for this study) collected at Manjakatompo, Madagascar, which he identified as *Afrarchaea godfreyi*. However, Legendre noted that these specimens were different from *A. godfreyi* in some details (i.e., the absence of prominent cephalic spines and the absence of abdominal sclerotization). These differences suggest that these specimens are not *A. godfreyi*. Unfortunately, in the CAS collections only a single juvenile specimen has been collected from Manjakatompo. This specimen also has reduced spines on the cephalon (only one rudimentary spine is present), which is again suggestive that this specimen is not *A. godfreyi*. Furthermore, many *Eriauchenius* species endemic to Madagascar have the short fat “neck” typical of most *Afrarchaea* (other than *A. cornutus* and *A. ansieae*) and also have reduced spination on the cephalon. The species *E. fisheri*, *E. goodmani* sp. n., *E. harveyi* sp. n., *E. mahariraensis*, *E. ratsirarsoni*, *E. sama* sp. n., and *E. wunderlichi* sp. n. all superfically resemble *Afrarchaea* in terms of carapace shape (Figs 9A, 10A, 11A, 12A, 15A). *E. mahariraensis* and *E. fisheri* were originally described as *Afrarchaea* based solely on carapace shape (Lotz 2003). Current research on archaeids shows that carapace shape has evolved in parallel, with similar morphs evolving repeatedly (Wood 2017; Wood et al. 2015; Wood et al. 2007), suggesting that carapace shape is not a good diagnostic trait for these genera. Phylogenetic analysis (Wood et al. 2015) and morphological examination of numerous African and Madagascan specimens suggests instead that the African and Madagascan species are short-range endemics that are restricted to either southern Africa or Madagascar, but not both (Fig. 1). Furthermore, after over 10 years of extensive collecting in Madagascar by CAS researchers, specimens of *Afrarchaea godfreyi* have never been found. For these reasons we propose that the distribution of *Afrarchaea godfreyi* be restricted to South Africa until evidence suggests otherwise.

**Figure 29.**
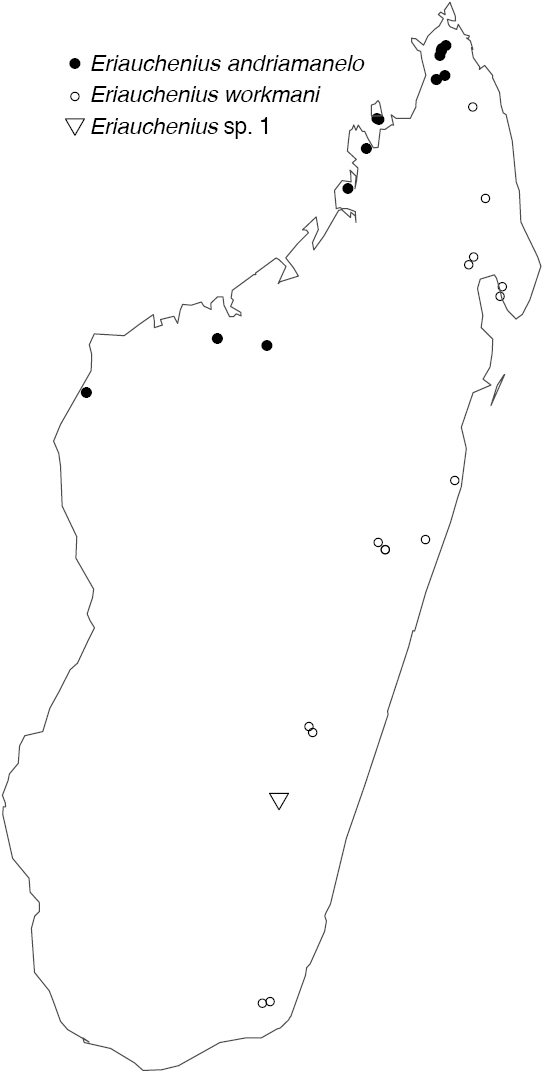
Distribution map for *Eriauchenius* species.

**Figure 30.**
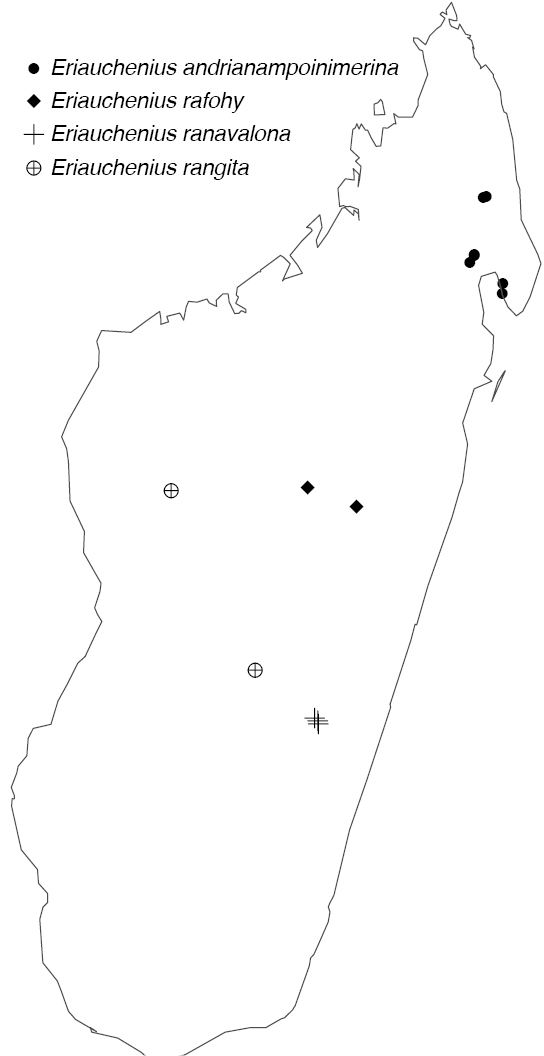
Distribution map for *Eriauchenius* species.

**Figure 31.**
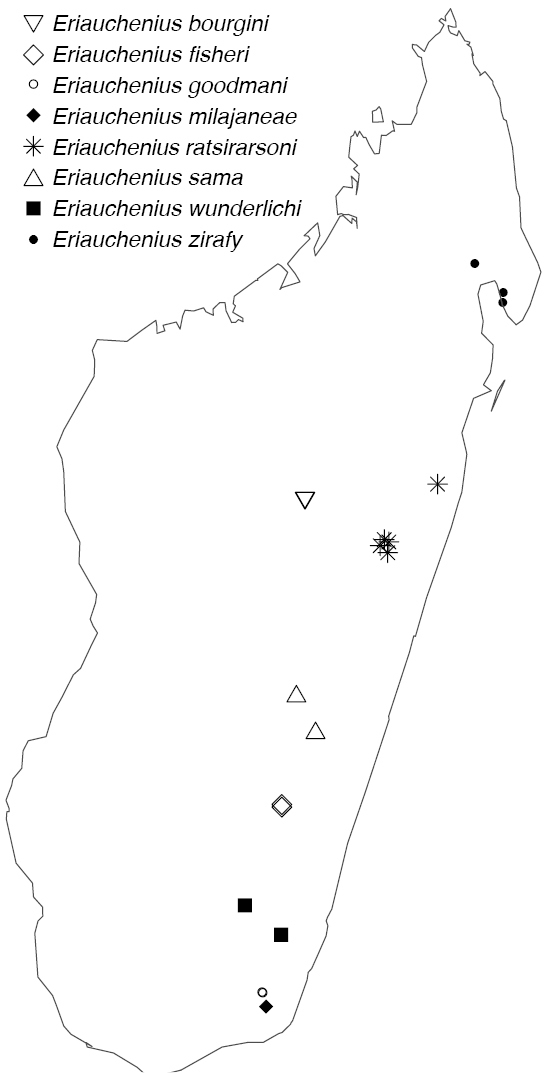
Distribution map for *Eriauchenius* species.

**Figure 32.**
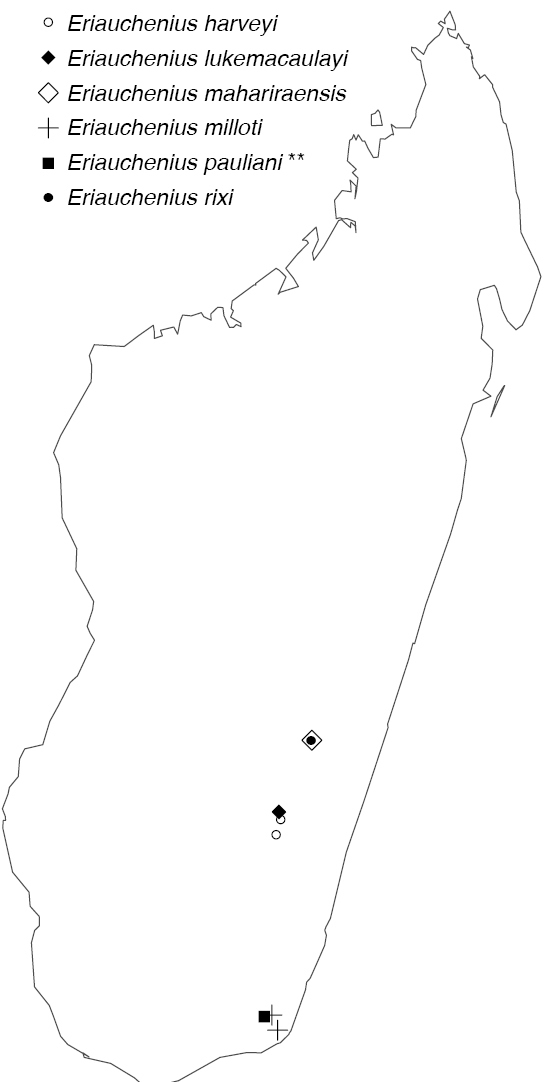
Distribution map for *Eriauchenius* species. ** The distribution for *E. pauliani* is an approximation as the holotype does not have latitude/longitude data.

**Figure 33.**
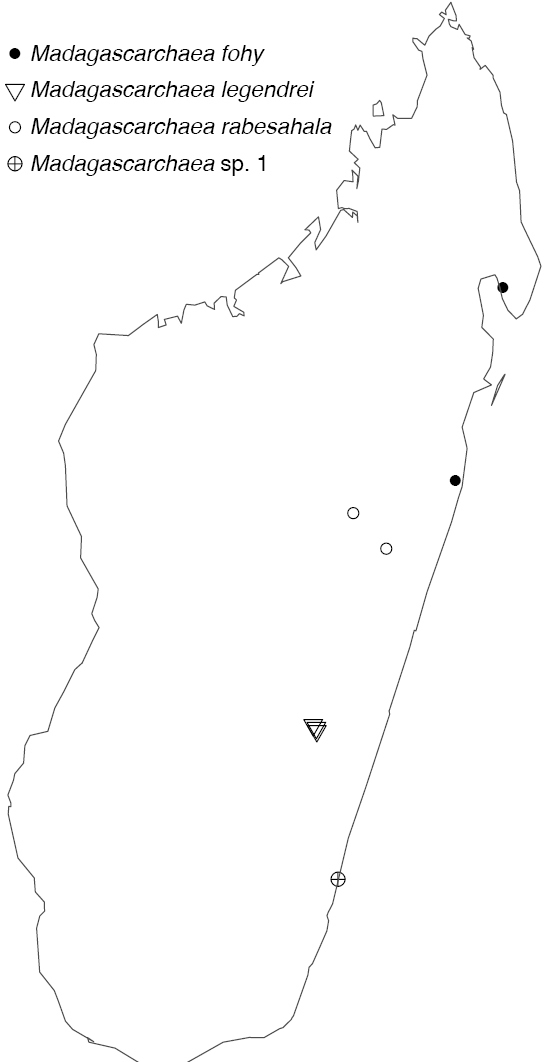
Distribution map for *Madagascarchaea* species.

**Figure 34.**
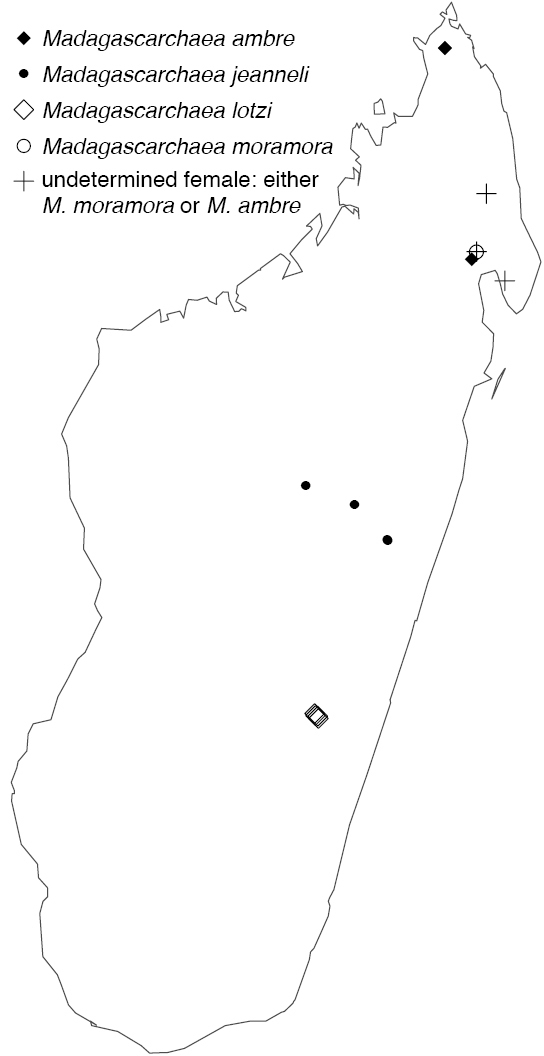
Distribution map for *Madagascarchaea* species.

## Supplementary Material

XML Treatment for Archaeidae

XML Treatment for Eriauchenius

XML Treatment for Eriauchenius andriamanelo

XML Treatment for Eriauchenius andrianampoinimerina

XML Treatment for Eriauchenius rafohy

XML Treatment for Eriauchenius ranavalona

XML Treatment for Eriauchenius rangita

XML Treatment for Eriauchenius workmani

XML Treatment for Eriauchenius fisheri

XML Treatment for Eriauchenius goodmani

XML Treatment for Eriauchenius harveyi

XML Treatment for Eriauchenius wunderlichi

XML Treatment for Eriauchenius bourgini

XML Treatment for Eriauchenius lukemacaulayi

XML Treatment for Eriauchenius mahariraensis

XML Treatment for Eriauchenius milajaneae

XML Treatment for Eriauchenius milloti

XML Treatment for Eriauchenius pauliani

XML Treatment for Eriauchenius ratsirarsoni

XML Treatment for Eriauchenius rixi

XML Treatment for Eriauchenius sama

XML Treatment for Eriauchenius zirafy

XML Treatment for Madagascarchaea

XML Treatment for Madagascarchaea fohy

XML Treatment for Madagascarchaea legendrei

XML Treatment for Madagascarchaea rabesahala

XML Treatment for Madagascarchaea jeanneli

XML Treatment for Madagascarchaea lotzi

XML Treatment for Madagascarchaea moramora

XML Treatment for Afrarchaea
